# Structure, mechanism and lipid-mediated remodeling of the mammalian Na^+^/H^+^ exchanger NHA2

**DOI:** 10.1038/s41594-022-00738-2

**Published:** 2022-02-16

**Authors:** Rei Matsuoka, Roman Fudim, Sukkyeong Jung, Chenou Zhang, Andre Bazzone, Yurie Chatzikyriakidou, Carol V. Robinson, Norimichi Nomura, So Iwata, Michael Landreh, Laura Orellana, Oliver Beckstein, David Drew

**Affiliations:** 1 Department of Biochemistry and Biophysics, Stockholm University, Stockholm, Sweden; 2 Center for Biological Physics, Department of Physics, Arizona State University, Tempe, AZ USA; 3 Nanion Technologies GmbH, Munich, Germany; 4 Department of Chemistry, University of Oxford, Oxford, UK; 5 Graduate School of Medicine, Kyoto University, Konoe-cho, Yoshida, Sakyo-ku, Kyoto, Japan; 6 Department of Microbiology, Tumor and Cell Biology, Karolinska Institutet, Stockholm, Sweden; 7 Department of Oncology–Pathology, Karolinska Institutet, Stockholm, Sweden

**Keywords:** Membrane structure and assembly, Membrane lipids, Supramolecular assembly, Cryoelectron microscopy

## Abstract

The Na^+^/H^+^ exchanger SLC9B2, also known as NHA2, correlates with the long-sought-after Na^+^/Li^+^ exchanger linked to the pathogenesis of diabetes mellitus and essential hypertension in humans. Despite the functional importance of NHA2, structural information and the molecular basis for its ion-exchange mechanism have been lacking. Here we report the cryo-EM structures of bison NHA2 in detergent and in nanodiscs, at 3.0 and 3.5 Å resolution, respectively. The bison NHA2 structure, together with solid-state membrane-based electrophysiology, establishes the molecular basis for electroneutral ion exchange. NHA2 consists of 14 transmembrane (TM) segments, rather than the 13 TMs previously observed in mammalian Na^+^/H^+^ exchangers (NHEs) and related bacterial antiporters. The additional N-terminal helix in NHA2 forms a unique homodimer interface with a large intracellular gap between the protomers, which closes in the presence of phosphoinositol lipids. We propose that the additional N-terminal helix has evolved as a lipid-mediated remodeling switch for the regulation of NHA2 activity.

## Abstract (web summary)

NHA2 exchanges sodium ions for protons across cell membranes, and its activity is linked to the pathogenesis of diabetes mellitus and essential hypertension in humans. Drew et al. report the cryo-EM structure of NHA2 in detergent and nanodiscs.

## Main

**Fig. 1:**
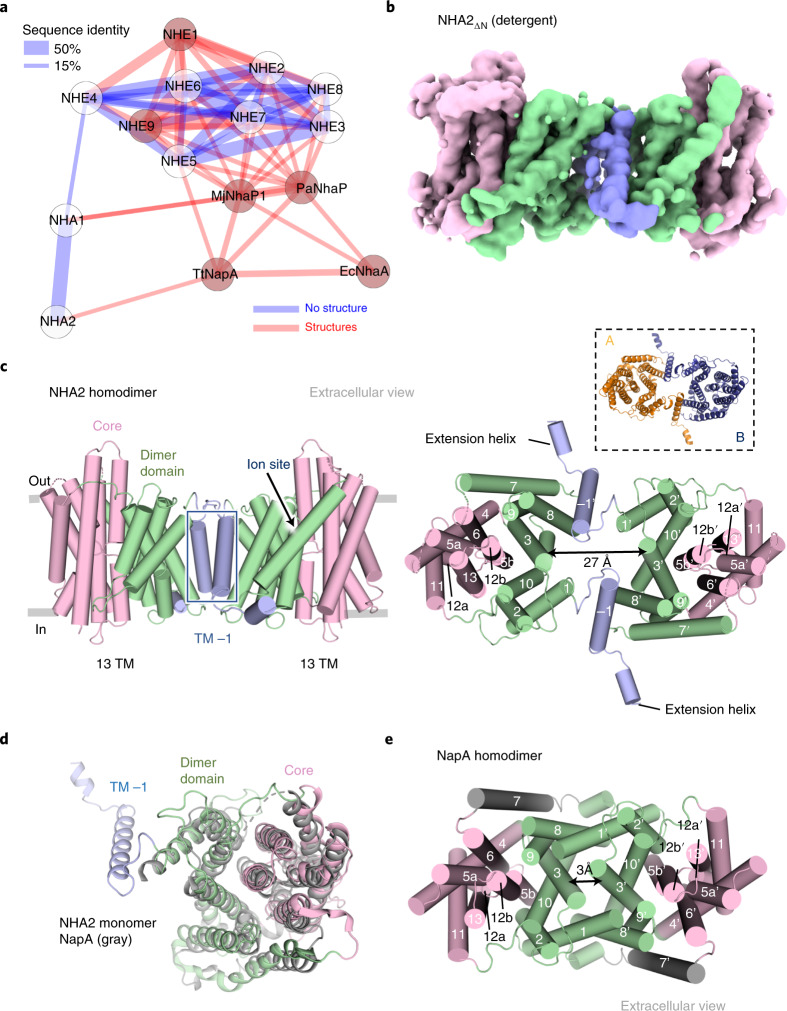
The cryo-EM structure of NHA2 reveals a domain-swapped homodimer. **a**, Phylogenetic tree of the canonical human NHE1–9 (SLC9A1–9) cluster compared to human NHA1 and NHA2 (SLC9B1–2) and bacterial members NapA (*Thermus thermophilus*), NhaP1 (*Methanococcus janashi*), NhaP (*Pyrococcus abyssi*) and NhaA (*Escherichia coli*). **b**, Cryo-EM density map of NHA2_ΔN_ in detergent, showing the 6-TM core ion transport domains (colored in pink), the dimer domain (colored in green) and the N-terminal domain-swapped helix, TM –1 (colored in blue). **c**, Cartoon representation of dimeric NHA2 from the side (left) and a top view from the extracellular side (right). The ion-translocation 6-TM domain (transport) is colored in pink, the dimerization domain in green and the N-terminal domain-swapped transmembrane helix (TM –1) in blue, with the respective transmembrane helices enumerated. Inset (dashed box): cartoon representation from the extracellular side, with each monomer colored individually. **d**, Cartoon representation of the 14-TM NHA2 monomer from the extracellular side and colored as in **c**, superimposed onto the 13-TM outward-facing structure of NapA (PDB 4BWZ) in gray. **e**, Cartoon representation of the NapA homodimer, colored as for NHA2 in **c** to highlight that, in the absence of the additional N-terminal helix TM –1, an extensive and more compact oligomer is formed compared to NHA2.

Intracellular salt, pH and cell volume are tightly regulated for cell survival^1^. The transmembrane exchange of protons (H^+^) for either sodium (Na^+^) or lithium (Li^+^) ions by Na^+^/H^+^ exchangers (NHEs) is central to this homeostatic process^1–3^. In mammals, there are 13 distinct NHE orthologs that belong to the cation:proton antiporter (CPA) superfamily, with differences in tissue and organellar localization, kinetics, regulation and substrate preferences^2–4^. NHE1 to NHE9 (solute carrier family 9 members A1–9) belong to the CPA1 clade, and are well known for their roles in human physiology, such as Na^+^ reabsorption in the kidney and in acid–base homeostasis^2,5–7^. By contrast, the mammalian CPA2 clade members NHA1 and NHA2 (SLC9 family 9 members B1 and B2) were more recently identified^4,8,9^ and share a closer evolutionary relationship to bacterial Na^+^/H^+^ antiporters^9^ (Fig. 1a). Based on tissue expression, genome location and phloretin sensitivity, human NHA2 was proposed to be the candidate gene for the Na^+^(Li^+^) countertransport activity associated with the development of essential hypertension and diabetes in humans^9–12^. Indeed, NHA2 aids in sodium reabsorption in the kidney^13,14^ and is a critical component of the with-no-lysine kinase 4-sodium-chloride cotransporter (WNK4-NCC) pathway in the regulation of blood pressure^14^. Furthermore, in vitro and in vivo studies show that NHA2 contributes to β-cell insulin secretion^15^. Because of its closer sequence similarity with bacterial antiporters, NHA2 was initially thought to partially localize to mitochondria^16^, but it was later established that its predominant localization is in endosomes and lysosomes^17^. NHA2 co-localizes with V-type H^+^-ATPase in all these intracellular organelles^17,18^ wherein cation extrusion is driven by an inwardly directed proton gradient^12^. Interestingly, NHA2 can also localize to the plasma membrane in specialized cells, such as in the apical membrane of kidney cells, which contains a plasma-membrane-located V-type H^+^-ATPase^12,13^, or to synaptic-like microvesicles^8,17^. Pairwise alignment shows that NHA2 harbors the conserved ion-binding aspartate residues that make up the well-described ‘DD-motif’^19,20^ found in electrogenic bacterial Na^+^/H^+^ antiporters, in which the proton-motive force energizes Na^+^ export. However, it is unclear whether NHA2 performs electroneutral (1:1) or electrogenic transport (2:1), as in the electrogenic bacterial homologs. The physiological direction of H^+^-driven Na^+^ efflux, however, agrees with the observation that in the fruit fly *Drosophila melanogaster*, NHA2 expression specifically protected against sodium-salt stress^18^. Na^+^/H^+^ exchangers form physiological homodimers^2,21^, with the monomers made up of a 6-transmembrane (TM) ion-translocation domain (or core domain) and a dimerization domain^22–27^. Recent structures of mammalian NHE1^25^ and NHE9^23^ were found to be highly similar to bacterial Na^+^/H^+^ antiporter structures with 13 TMs^22,24,26,28^. So far, the core domain has proven to be structurally similar, with most of the structural divergence in the dimerization domain instead^23^. Na^+^/H^+^ exchangers operate by an ‘elevator’ alternating-access mechanism^23–25,29,30^. In an elevator mechanism, the ion is transported by the core domain against the dimerization domain, which remains fixed due to oligomerization^30^. In this Article we aim to determine the structure of the evolutionary-divergent NHA2 homodimer and the molecular basis for its ion-exchange mechanism.

## Results

### Cryogenic electron microscopy structure of NHA2_ΔN_ in detergent

**Extended Data Fig. 1:**
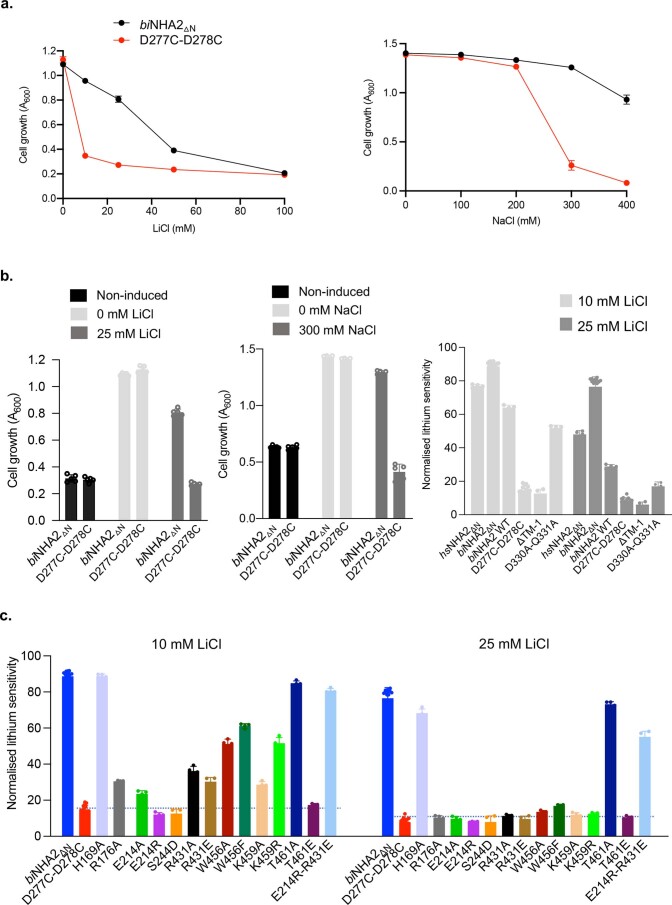
Functional complementation of salt-sensitive yeast strain AB11c by heterologous expression of *bison* NHA2 and mutants. **a**. AB11c strain was transformed with *bison* NHA2_ΔN_ or the corresponding construct with both Asp277 and Asp278 have been substituted with cysteine (D277C-D278C); *human* NHA2 numbering is D278, D279. Yeast were cultivated as outlined in Methods in -URA media supplemented with 2% galactose and either LiCl (left) or NaCl (right) and growth was determined by optical density of the culture at 600 nm (OD_600_) after 48 or 72 h at 30 °C, respectively. Errors bars represent the mean values ± s.d. of: n = 5 biologically independent samples for biNHA2_ΔN_ and D277C-D278C in LiCl; n = 4 biologically independent samples for biNHA2_ΔN_ in NaCl; n = 3 biologically independent samples for D277C-D278C in NaCl. **b**. *left:* as in a., for NHA2 constructs grown in in the absence or presence of either 0 or 25 mM LiCl, *middle:* 0 or 300 mM NaCl and *right*: normalized lithium sensitivity based on growth of AB11c yeast cells grown in the absence of salt-stress. Errors bars represent mean values ± s.d of n = 5 biologically independent samples. **c**. normalized lithium sensitivity at 10 and 20 mM LiCl concentrations as described in b. (see Supplementary Fig. 1 for expression level and FSEC chromatograms and Supplementary Fig. 3 for LiCl growth curves). Errors bars represent the mean values ± s.d of n = 5 biologically independent samples. Source data

**Extended Data Fig. 2:**
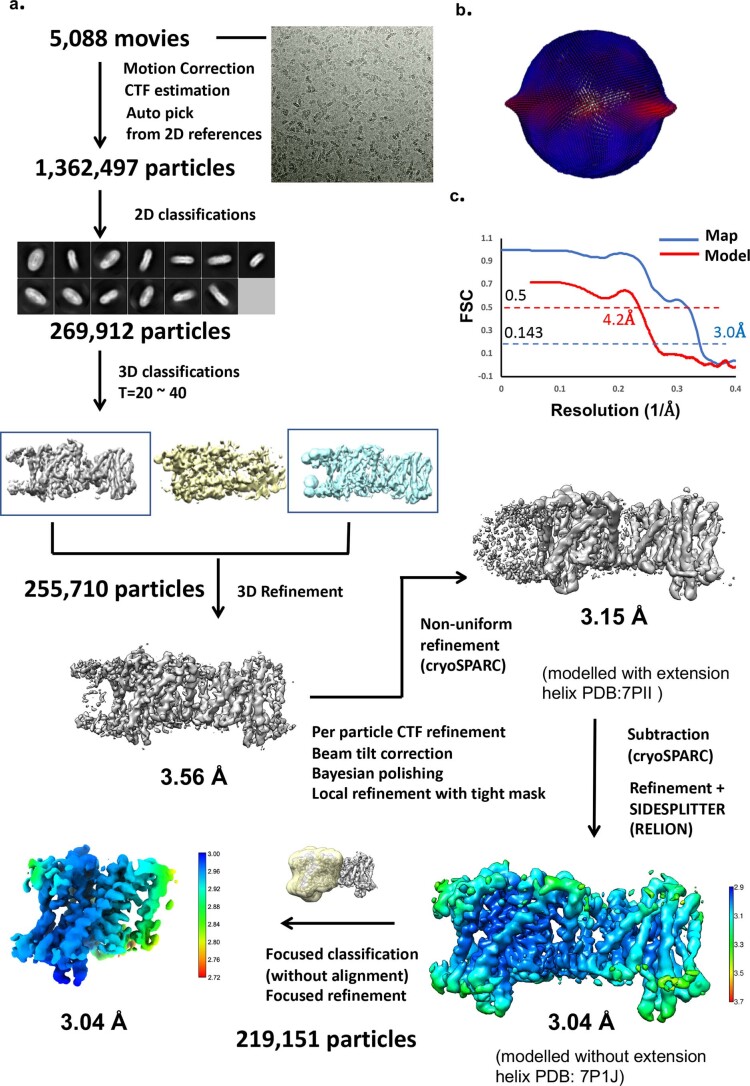
The data-processing workflow of *bison* NHA2_ΔN_ in detergent. **a**. The dataset contained 5,088 movies that were corrected by MotionCor2 and CTFFind. After reference based auto-picking, 1,362,497 particles were selected. Several rounds of 2D classification were performed, yielding 269,912 particles, which were subjected to 3D classification. One of the three 3D classes was selected, and it contained 255,710 particles. After several rounds of refinement with global and local search using CTF refine, polishing and masking 3.56 Å was achieved. After non-uniform refinement in cryoSPARC, a resolution of 3.15 Å was achieved at gold standard FSC (0.143), which was later used for modeling the N-terminal helix (after the final model was built from the next step of refinement). The final maps were obtained after particle subtraction of the micelle region in cryoSPARC and SIDESPLITTER in Relion at a resolution of 3.04 Å was achieved at gold standard FSC (0.143), with a local resolution range of 2.9 to 3.7 Å. To improve map resolution further focused classification and refinement was carried out and used to guide model building of the NHA2_ΔN_ homodimer (PDB: 7P1J). **b**. Angular distributions of the final reconstruction. **c**. The FSC curve for the final NHA2_ΔN_ homodimer in detergent (PDB:7P1J).

**Extended Data Fig. 3:**
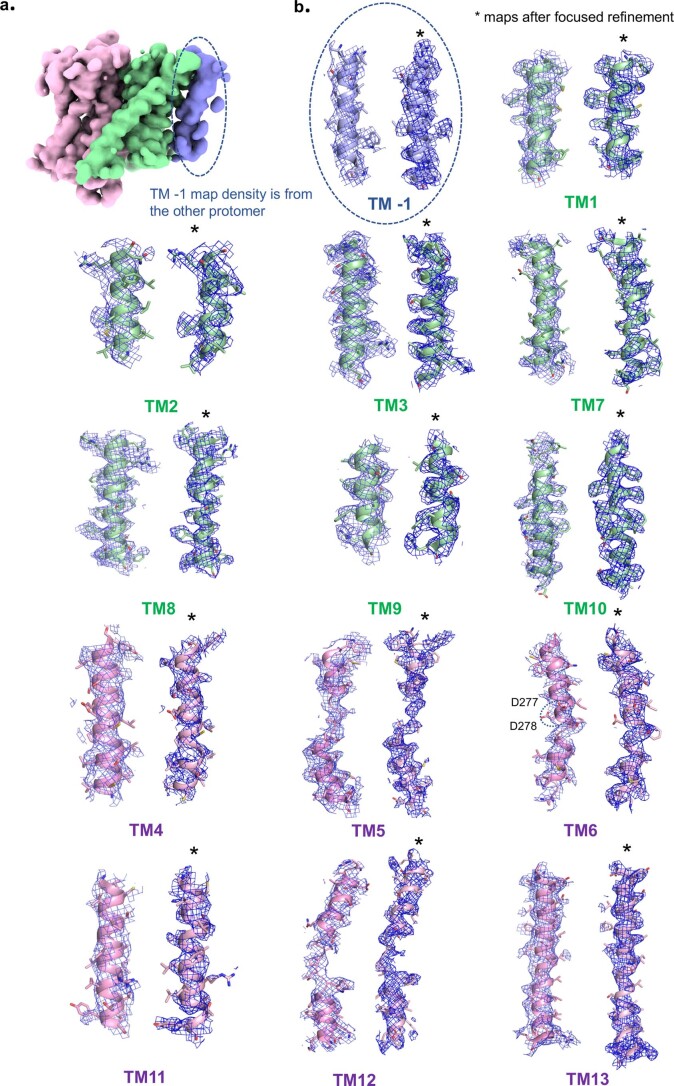
Cryo-EM density of *bison* NHA2 _ΔN_ in detergent before and after focused refinement. **a**. Cryo-EM density map of NHA2_ΔN_ after focused refinement with the 6-TM core transport domain (colored in pink), the dimer domain (colored in green) and the N-terminal domain-swapped helix TM –1 from the neighboring protomer (blue and circled). **b**. Cryo-EM density map for protomer A before (left) and after (right) focused refinement. The protomer is made up from TM 1 to 13 of one monomer and TM -1 of the other monomer; as such oligomerization contacts are retained in the focused refined map. In TM6 the aspartate residues D277 and D278 (encircled) in TM6 were modeled based on the rotomer position in NapA at pH 8.0 (PDB id: 4BWZ).

Bison NHA2 was selected for structural studies as it was more stable following detergent extraction from its overexpression host *Saccharomyces cerevisiae* than human NHA2 and other mammalian homologs investigated (Methods). The bison NHA2 construct used for structural studies is referred to as NHA2_ΔN_, because it does not include the first 69 residues of the N-terminal tail. The N terminus is poorly conserved and was removed to improve expression and reduce predicted disorder^31^ (Methods and Supplementary Fig. 1). The truncated NHA2_ΔN_ sequence shares 97% sequence identity to human NHA2_ΔN_ (Supplementary Fig. 2). It has been shown previously that only functional human NHA2 can rescue growth in the salt-sensitive *S. cerevisiae* AB11c strain^9,32^, which lacks the main Na^+^- and K^+^-extrusion systems. We confirmed that bison NHA2_ΔN_ was expressed in the AB11c strain and complemented growth under Li^+^- and Na^+^-salt stress conditions to at least the same extent as the full-length construct or human NHA2_ΔN_ (Extended Data Fig. 1a,b and Supplementary Figs. 1a,b and 3a,b). By contrast, poor *S. cerevisiae* AB11c growth was apparent under Na^+^- or Li^+^-salt stress conditions for either non-induced cells or the double ion-binding aspartate mutant of bison NHA2_ΔN_ Asp277Cys-Asp278Cys, previously shown to abolish human NHA2 activity^9^ (Extended Data Fig. 1a,b and Supplementary Figs. 1a,b and 3b). The bison NHA2_ΔN_ sample preparation was subsequently optimized for grid preparation, cryo-EM data acquisition and structural determination at pH 8.0 (Extended Data Fig. 2a and Methods). Single-particle analysis produced two-dimensional (2D) class averages corresponding to predominantly side views of the NHA2_ΔN_ homodimer. To improve 3D reconstruction, detergent subtraction and non-uniform refinement were performed in cryoSPARC (v.2.14.2)^33^. The final cryo-EM map of NHA2_ΔN_ in detergent was estimated at 3.0 Å (Fourier shell correlation (FSC) = 0.143 criterion), although protomer A was consistently better aligned than protomer B, and was therefore resolved at a higher resolution (Table 1 and Extended Data Fig. 2). Overall, the structure of NHA2_ΔN_ in detergent has a model resolution at 4.2 Å (FSC = 0.5), which is reflected in the fact that some side chains in the core domain and the connecting TM6–TM7 loop could not be modeled, probably due to flexibility (Fig. 1b, Extended Data Fig. 2b,c and Methods). Focused classification and refinement of protomer A was therefore carried out, which reduced the heterogeneity and improved map quality for model building (Extended Data Figs. 2 and 3a,b).

**Table 1:** Data collection, processing and refinement statistics of bison NHA2 structures

	Detergent outward facing w/o extended helix EMD-13162, PDB 7P1J	Detergent outward facing with extended helix EMD-13161, PDB 7PII	Nanodiscs outward facing w/o extended helix EMD-13163, PDB 7P1K
**Data collection and processing**			
Magnification	165,000		130,000
Voltage (kV)	300		300
Electron exposure (e^–^/Å^2^)	80		63.5
Defocus range (μm)	0.7–2.5		0.6–2.2
Pixel size (Å)	0.83		0.68
Symmetry imposed	*C*1		*C*1
Initial particle images (no.)	1,326,947		3,055,998
Final particle images (no.)	269,412		362,665
Map resolution (Å)	3.04	3.15	3.58
FSC threshold	0.143	0.143	0.143
Map resolution range (Å)	2.9–4.6	3.0–4.8	3.2–4.6
**Refinement**			
Initial model used (PDB code)	4BWZ		
Model resolution (Å)	3.8	4.0	3.1
FSC threshold	0.143	0.143	0.143
Model resolution range (Å)			
Map sharpening *B* factor (Å^2^)	−80.3	−58.2	−100
Model composition			
Nonhydrogen atoms	6,502	6,644	6,834
Protein residues	868	884	866
Ligands			PI: 4
			CHS: 8
*B* factors (Å^2^)			
Protein	151.99	185.09	33.48
Ligand			34.09
R.m.s. deviations			
Bond lengths (Å)	0.004	0.003	0.009
Bond angles (°)	0.659	0.642	1.744
Validation			
MolProbity score	2.5	2.34	2.52
Clashscore	10.73	8.60	12.49
Poor rotamers (%)	4.73	5.2	7.90
Ramachandran plot			
Favored (%)	93.14	95.21	96.50
Allowed (%)	6.86	4.79	3.5
Disallowed (%)	0	0	0

**Extended Data Fig. 4:**
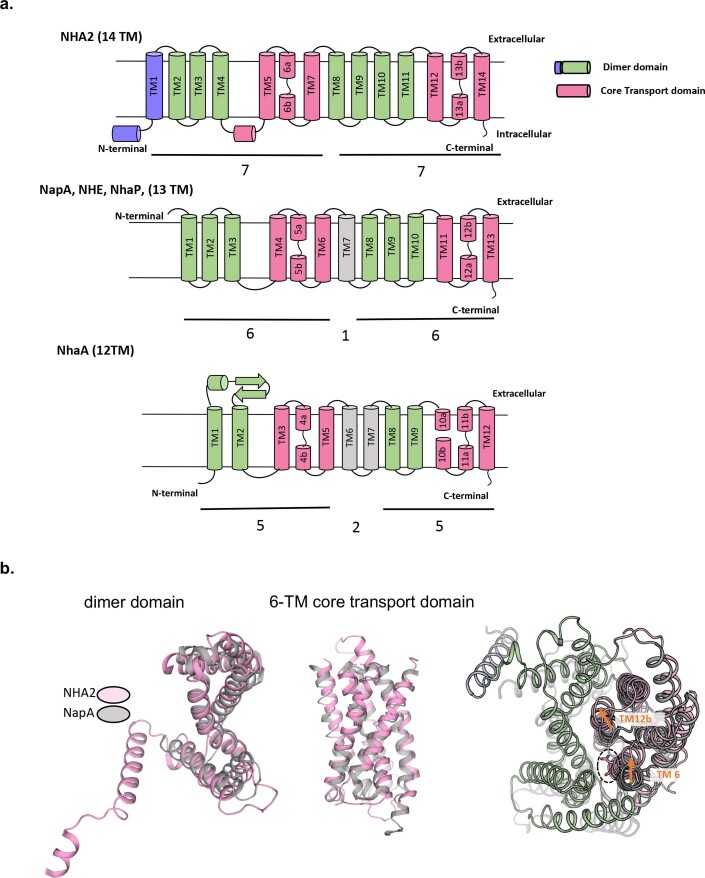
Topology comparison of NHA2 to 13-TM and 12-TM Na^+^/H^+^ antiporter homologs and structural comparison to NapA. **a**. *top:* NHA2 has 14 TMs with the N-and C-terminus located in the cytoplasm and is made up of two 7-TM structural-inverted repeats that form the 6-TM core domain (pink), dimerization domain (green) and N-terminal domain-swapped helix TM -1 (blue); note, the linker helix TM7 has been assigned with the dimer domain as is TM -1, even though technically both these helices could be assigned as independent from both core and dimer domains. *middle:* NapA, NHE and NhaP members with 13 TMs (N-terminus extracellular and C-terminus cytoplasm) is the most common topology seen in the Na^+^/H^+^ exchangers, which are made up of two 6-TM structural-inverted repeats that form the 6-TM core domain (pink), dimerization domain (green) and non-symmetry-related linker helix (gray). *bottom:* NhaA is the only Na^+^/H^+^ exchanger seen with 12-TMs and it is made up of two 5-TM structural-inverted repeats and forms the 6-TM core domain (pink), dimerization domain that contains a β-hairpin between TM1 and TM2 (green) and two non-symmetry related helices (gray). **b**. *left:* cartoon representation of the dimer domain of the NHA2 monomer (pink) showing the structural conservation compared to the dimer domain of the bacterial homolog NapA domains (gray) following superimposition. *middle:* as in left but for the transport domain. *right:* structural superimposition of the NHA2 (pink and green) and NapA (gray) monomers. The arrow highlights the structural differences in the core domain helices TM12b and TM6 between outward-facing NHA2 and NapA structures, with the strictly conserved Asp278 (Asp157 in NapA) shown in stick form (encircled).

Unexpectedly, NHA2_ΔN_ is composed of 14-TM segments, rather than the 13 TMs observed in the mammalian Na^+^/H^+^ exchangers NHE1^25^ and NHE9^23^ and the bacterial Na^+^/H^+^ antiporters NapA^24^, *Mj*NhaP^26^ and *Pa*NhaP^27^ or the 12-TMs observed in NhaA^22^ (Fig. 1c,d and Extended Data Fig. 4a). To facilitate comparison to the 13-TM members, the first helix in NHA2_ΔN_ was designated TM –1. The observed additional N-terminal helix TM –1 is part of the dimer domain in NHA2_ΔN_ and is domain-swapped, mediating homodimerization by interacting with TM8 and the TM8–TM9 loop in the neighboring protomer (Fig. 1c). As a result of the tilted angle of TM –1 and the length of the connecting loop to TM1, the NHA2_ΔN_ protomers are held apart by ~ 25 Å on the intracellular side (Fig. 1c). Although NHA2_ΔN_ homodimerization appears very different to any Na^+^/H^+^ exchanger observed so far, the NHA2_ΔN_ monomer has a high degree of structural conservation with the outward-facing monomer of NapA, but with the additional TM −1 helix (Fig. 1d,e and Extended Data Fig. 4b). Before masked refinement we also observed additional map density for a predicted short helical segment prior to TM −1, which sits at right angles to the protein (Fig. 1c, Supplementary Fig. 4a–c and Methods).

### Oligomerization is required for optimal NHA2 function

**Fig. 2:**
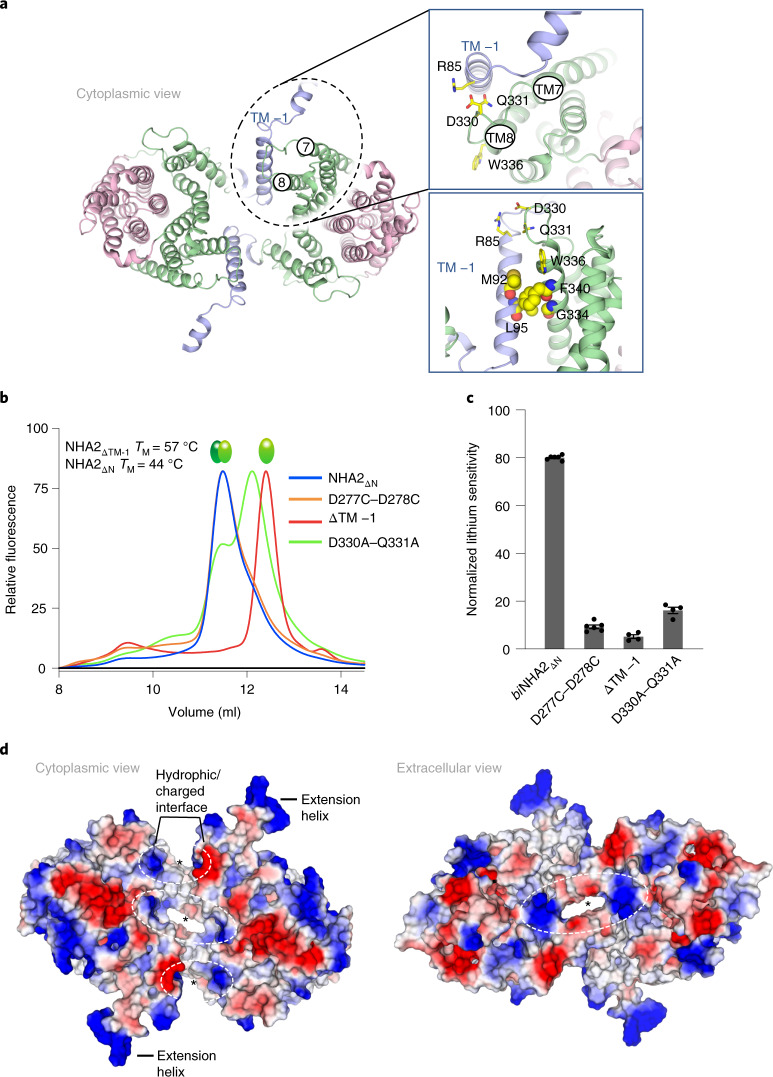
NHA2_ΔN_ oligomerization. **a**, Cartoon representation of the NHA2_ΔN_ homodimer viewed from the cytoplasmic side and colored as in Fig. 1c, with the dotted circle highlighting one of the two oligomerization contacts formed between TM −1 (blue) and TM8 (green) on the neighboring protomer. Top inset: zoomed-in view showing the potential polar contacts between the strictly conserved residues D330–Q331 in the TM8–TM9 loop of one protomer (yellow sticks, labeled) and Arg85 in TM −1 of the other protomer (yellow sticks, labeled). Bottom inset: zoomed-in view showing the oligomerization interactions formed by hydrophobic residues (yellow spheres, labeled) between TM −1 (blue) and TM8 (green). **b**, Representative FSEC traces of DDM-CHS solubilized NHA2_ΔN_ (blue) and NHA2_ΔN_ non-functional mutant D277C–D278C (orange) from membranes isolated following heterologous expression in the salt-sensitive yeast strain *S. cerevisiae* AB11c^9,32^. FSEC traces were compared to the NHA2_ΔN_ loop mutant D330A–Q331A (green) and the TM −1 deletion mutant NHA2_ΔTM−1_ (red). Melting temperatures (*T*_M_) for purified NHA2_ΔN_-GFP and NHA2_ΔTM−1_-GFP in DDM/CHS were calculated from the melting curves in Supplementary Figs. 1c and 3c. **c**, Normalized lithium sensitivity of NHA2_ΔN_ and derived constructs grown in the AB11c strain in the presence of 20 mM LiCl (see Extended Data Fig. 1b and Supplementary Fig. 3b,c for cell growth in the presence of different lithium concentrations and non-induced controls). Errors bars represent the mean ± s.e.m. of *n* = 4 biologically independent samples. **d**, Electrostatic surface representation of the outward-facing NHA2_ΔN_ homodimer from the cytoplasm (left) and the extracellular side (right). The dashed circles and asterisks highlight the regions of positively charged surfaces between the two protomers. Source data

Oligomerization is thought to be required for Na^+^/H^+^ exchanger activity^21,34^ and in elevator proteins in general^30^, perhaps because homodimerization enables the core domain to move against a dimer domain that through dimerization becomes relatively immobile^30^. In NHA2_ΔN_, the dimerization contacts are small, made up solely from interactions between TM −1 in one protomer and TM8 in the other (Fig. 2a). Following removal of TM −1 (NHA2_ΔTM-1_) the shorter form was found to be well-folded with a similar expression level to NHA2_ΔN_ (Fig. 2b and Supplementary Fig. 1a,c). Based on size-exclusion chromatography profiles, however, the detergent-extracted NHA2_ΔTM–1_ variant migrated as a monomer (Fig. 2b), and was unable to complement Li^+^-salt stress, with yeast growth indistinguishable from the transport-inactive Asp277Cys-Asp278Cys double mutant (Fig. 2c, Extended Data Fig. 1b and Supplementary Fig. 3c). Confocal microscopy confirmed the plasma membrane and vacuolar localization of the TM −1 mutant, which was comparable to either bison NHA2 and NHA2_ΔN_ or human NHA2_ΔN_ localization (Supplementary Fig. 5a,b). The cytoplasmic end of TM −1 in one protomer appeared to also interact with the TM8–TM9 loop in the other protomer via polar interactions to a highly conserved DQ-motif (Asp330, Gln331; Fig. 2a and Supplementary Fig. 2), but the map density for these side chains was of insufficient detail to confirm this. Remarkably, however, an Asp330Ala-Gln331Ala double mutant was enough to shift a large fraction of NHA2_ΔN_ homodimers into monomers (Fig. 2b). The Asp330Ala-Gln331Ala double mutant also failed to rescue growth of the host strain under high Li^+^ stress and showed no measurable differences in trafficking (Fig. 2c and Supplementary Figs. 1a, 3c and 5). Taken together, we can confirm that TM −1 is required for homodimerization, and furthermore that oligomerization is required for optimal NHA2_ΔN_ activity.

### Lipid analysis and cryogenic electron microscopy structure of NHA2_ΔN_ in nanodiscs

**Fig. 3:**
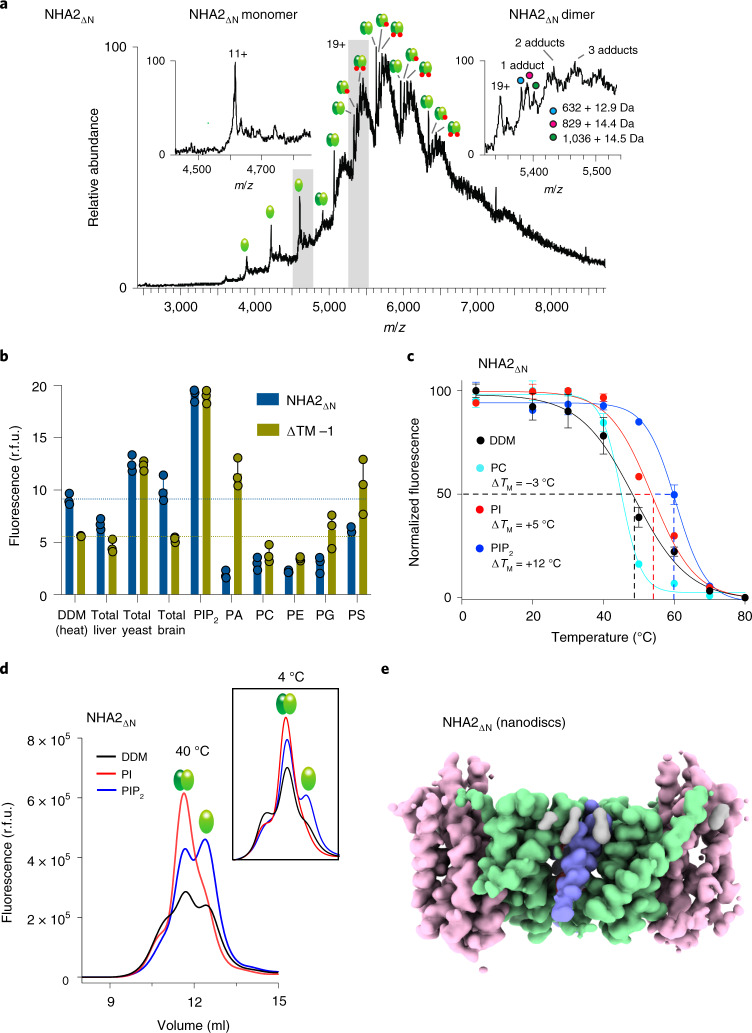
Lipid preferences of NHA2_ΔN_. **a**, The native high-resolution mass spectrum of purified NHA2_ΔN_ reveals a homodimer with multiple lipid adducts, as well as a small amount of lipid-free monomer (left inset). The masses of the first lipid adduct shown for the 19+ monomer are consistent with retention of a PI (829 ± 14.4 Da) or a PIP_2_ (1,036 ± 14.5 Da) molecule (right inset). Peaks shown as inserts are highlighted by grey bars in the full spectrum. **b**, Thermal stabilization of DDM-purified dimeric NHA2_ΔN_-GFP (blue bars) and NHA2_ΔTM−1_ (green bars) by lipids. Normalized mean fluorescence (r.f.u., relative fluorescence units) is shown after heating (*T*_M_ + 5 °C, for the respective forms) and centrifugation in the presence of either the detergent DDM or DDM-solubilized lipids. Error bars represent the mean ± s.e.m. of *n* = 3 independent experiments (Methods). **c**, Thermal shift stabilization of purified dimeric NHA2_ΔN_-GFP in the presence of DDM addition (black) compared to PIP_2_ in DDM (blue), POPC in DDM (cyan) and PI in DDM (red). The data are normalized fluorescence mean ± s.e.m. of *n* = 5 independent experiments for DDM, *n* = 3 independent experiments for PI and PC and *n* = 2 independent experiments for PIP_2_. The apparent melting temperature *T*_M_ was calculated with a sigmoidal four-parameter logistic regression function (Methods). **d**, Representative FSEC traces of DDM/CHS-purified NHA2_ΔN_ after heating at 40 °C for 10 min in the presence of DDM addition (black) compared to PIP_2_ in DDM (blue) or PI in DDM (red). Inset: as in the main panel, but prior to heating. **e**, Cryo-EM density map of NHA2_ΔN_ in nanodiscs with the 6-TM core ion-transport domains (colored in pink), the dimer domain (colored in green), cholesterol (gray) and the N-terminal domain-swapped helix TM −1 (blue). Source data

The interface between the NHA2_ΔN_ protomers contains both hydrophobic and positively charged regions that are likely to be lipid-filled in a biological membrane (Fig. 2d). Analysis of detergent-purified NHA2_ΔN_ from *S. cerevisiae* by high-resolution native MS^35^ reveals peaks corresponding in mass to the homodimer with multiple lipid adducts, whereas residual monomers are essentially lipid-free (Fig. 3a). The lipid peak with the lowest mass at 632 ± 12 Da does not correspond to any major lipid, whereas the second peak at 829 ± 14 Da fits most closely with the binding of a phosphatidylinositol (PI) lipid. Additional MS analysis of purified PI lipids from *S. cerevisiae* confirms that the second-most abundant PI lipid at 836 Da is closest to this mass (Supplementary Fig. 6). The most intense peak is 1,036 ± 14 Da, which is a close match to the molecular weight of the lipid phosphatidylinositol 4,5-bisphosphate (PIP_2_) at 1,042 Da (Fig. 3a).

To complement the native MS analysis, we screened a range of relevant lipids using a green fluorescent protein based thermal-shift assay (GFP-TS)^36^, which we had previously shown could detect the specific binding of negatively charged cardiolipin and PIP_2_ interactions to purified NhaA and NHE9 proteins, respectively^23,36^. Consistent with native MS analysis, we found that NHA2_ΔN_ was most thermostabilized by yeast polar lipids containing 26% PI and by the lipid PIP_2_ (Fig. 3b,c). By contrast, the NHA2_ΔTM−1_ mutant was indiscriminately thermostabilized by all negatively charged PIP_2_, phosphatidic acid (PA), phosphatidylglycerol (PG) and phosphatidylserine (PS) lipids (Fig. 3b and Methods). The disruption of the homodimer form, observed for the NHA2_ΔTM−1_ variant, could have arisen from the exposure of positively charged surfaces at the interface (Fig. 2d). Consistent with this interpretation, even without heating, the (−4)-charged lipid PIP_2_ disrupts a small fraction of NHA2_ΔN_ homodimers into monomers (Fig. 3d). During heating, PIP_2_ stabilizes both NHA2_ΔN_ homodimers and monomers. Given that native MS indicates that PIP_2_ is retained in the purified NHA2_ΔN_ homodimer, it is unclear whether this disruptive effect is due to the high concentration of PIP_2_ used in the assay or not. By contrast, although ~98% pure yeast PI is less thermostabilizing than PIP_2_, the addition of PI lipid has a profound effect on the stability of predominantly the NHA2_ΔN_ homodimer (Fig. 3c,d). Taken together, native MS and thermostability analysis reveal that PI lipids specifically bind to NHA2_ΔN_, and its addition stabilizes the homodimer.

**Fig. 4:**
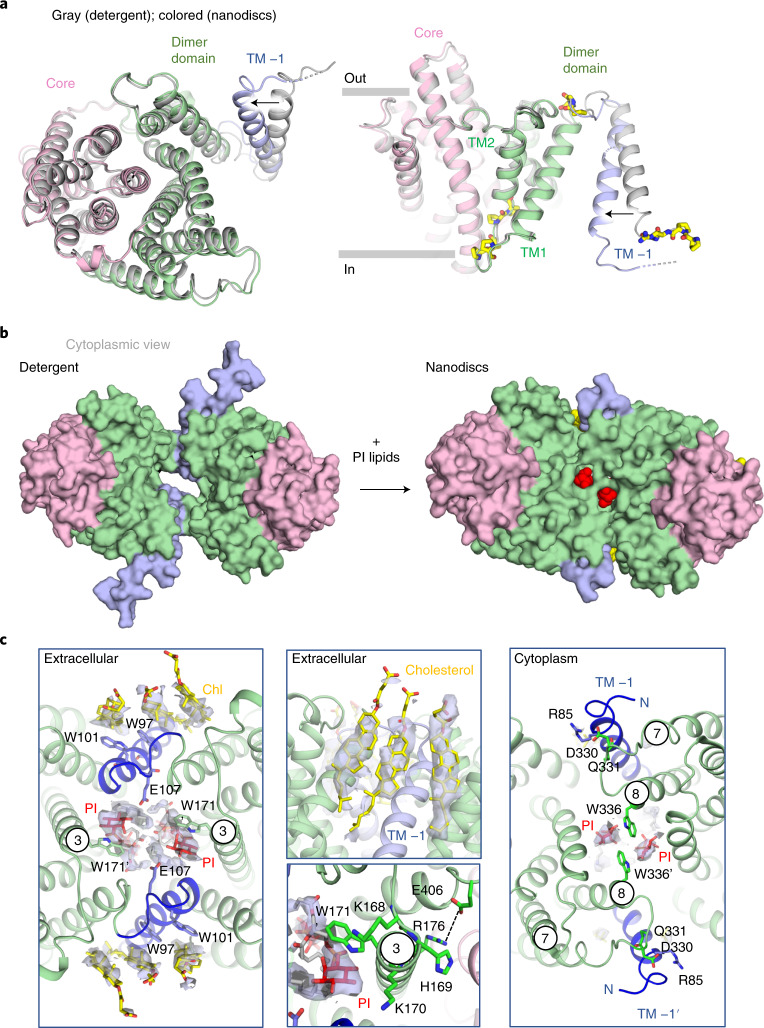
Lipid remodeling of NHA2_ΔN_ in nanodiscs. **a**, Left: cartoon representation of the 14-TM NHA2_ΔN_ monomer in nanodiscs, from the intracellular side (colored as in Fig. 1c), superimposed onto the NHA2 monomer in detergent (in gray) to highlight the movement of TM −1, as indicated by the arrow. Right: as in the left panel, but from the side and including the highly conserved proline residues in yellow stick form (Extended Data Fig. 1). **b**, Surface representation of the NHA2_ΔN_ structure from the cytoplasmic side in detergent (left) and in nanodiscs mixed with PI lipids (right). **c**, Left: cartoon representation of the NHA2 homodimer in nanodiscs, from the extracellular side, highlighting the bound PI lipids (red sticks, cryo-EM map in gray mesh) coordinated by tryptophan residues in TM3 at the protomer interface and cholesterol interacting with tryptophan residues in TM −1. Middle top: zoomed view showing the cryo-EM map density (gray mesh) for the cholesterol lipids (stick form, yellow) interacting with TM −1 at the dimerization interface. Middle bottom: zoomed view showing the cryo-EM map density for one of the PI lipids (stick form, red) interacting with W171 in TM3 and potentially E107 in TM −1. More extensively phosphorylated forms of the inositol moiety, at either C4 or C4/C5 for PI_4_P and PIP_2_ respectively, could be accommodated and enable additional interaction to the K168 and K170 residues. A salt bridge (dashed line) is also formed between residues R176 in TM3 and E406 in TM10. Right: cartoon representation of the NHA2 homodimer in nanodiscs, from the cytoplasmic side, highlighting the bound PI lipids (red, gray) coordinated by tryptophan residues, in TM8 at the protomer interface, that have come closer together by the movement of TM −1, which retains its interactions with the TM7–TM8 loop. Selected TM numbering are circled.

**Extended Data Fig. 5:**
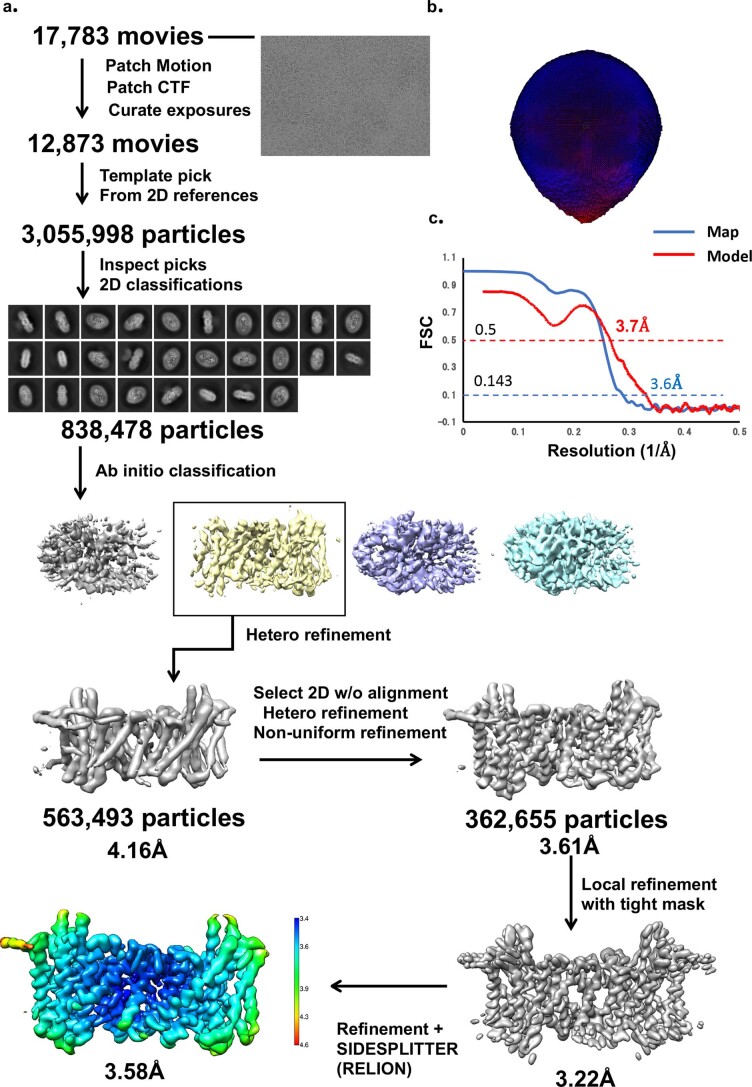
The data-processing workflow of *bison* NHA2 _ΔN_ in nanodiscs. **a**. The dataset contained 17,783 movies that were corrected by Patch motion correction and Patch CTF estimation in cryoSPARC. After template based auto-picking, 3,055,998 particles were selected. Several rounds of 2D classification were performed, yielding 838,438 particles, which were subjected to 3D classification. One of the three 3D main classes was selected, and it contained 563,493 particles. After non-uniform refinement in cryoSPARC, a resolution of 3.61 Å was achieved that was improved with local refinement and masking to 3.22 Å and after SIDESPLITTER in Relion a resolution of 3.5 Å was achieved at the gold standard FSC (0.143), with a local resolution range of 3.4 to 4.6 Å. **b**. Angular distributions of the final reconstruction for NHA2_ΔN_ in nanodiscs **c**. The FSC curve for the NHA2_ΔN_ in nanodiscs (PDB:7P1K).

**Extended Data Fig. 6:**
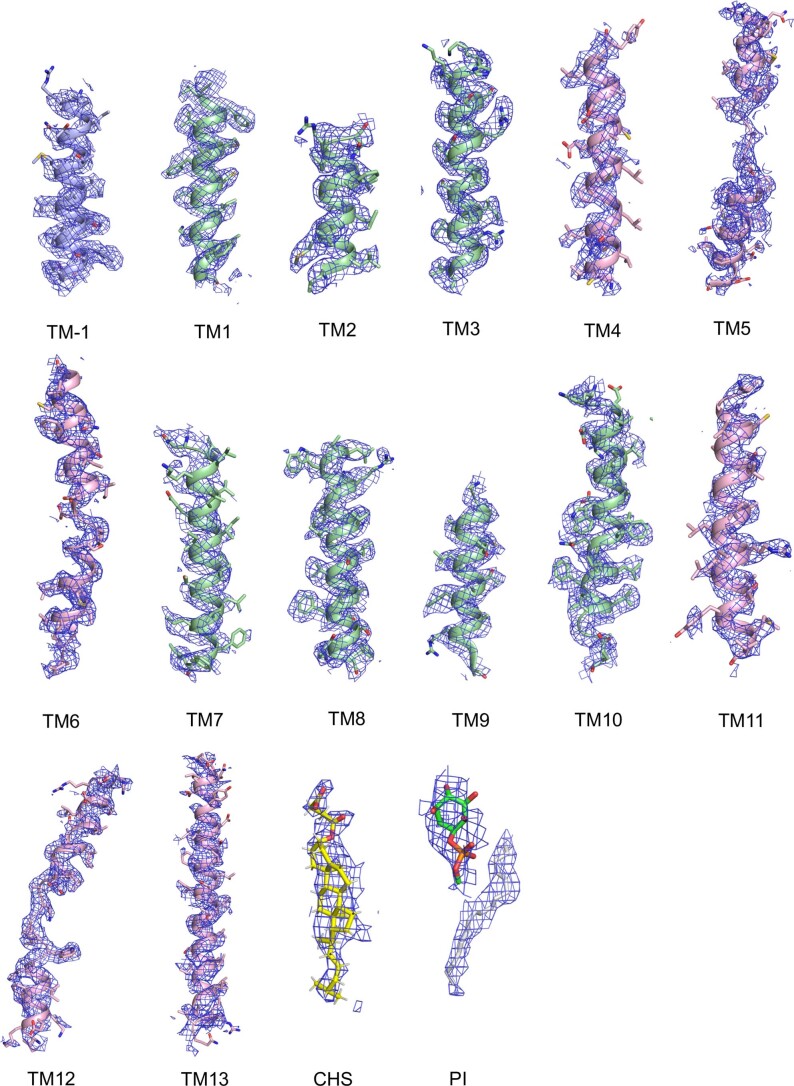
Cryo-EM density of *bison* NHA2 _ΔN_ in nanodiscs. Cryo-EM density map of the NHA2_ΔN_ helices in nanodiscs. The respective helices are shown in cartoon representation for the 6-TM core transport (pink), the dimer domain (green), the N-terminal domain-swapped helix TM –1 (blue), with their cryo-EM map density in blue mesh. Density for several lipids is also shown in blue mesh, and modeled lipids as sticks with CHS (yellow), PI-headgroup (green) and PI tail (gray); other lipids and map density is shown in Extended Data Fig. 7c.

**Extended Data Fig. 7:**
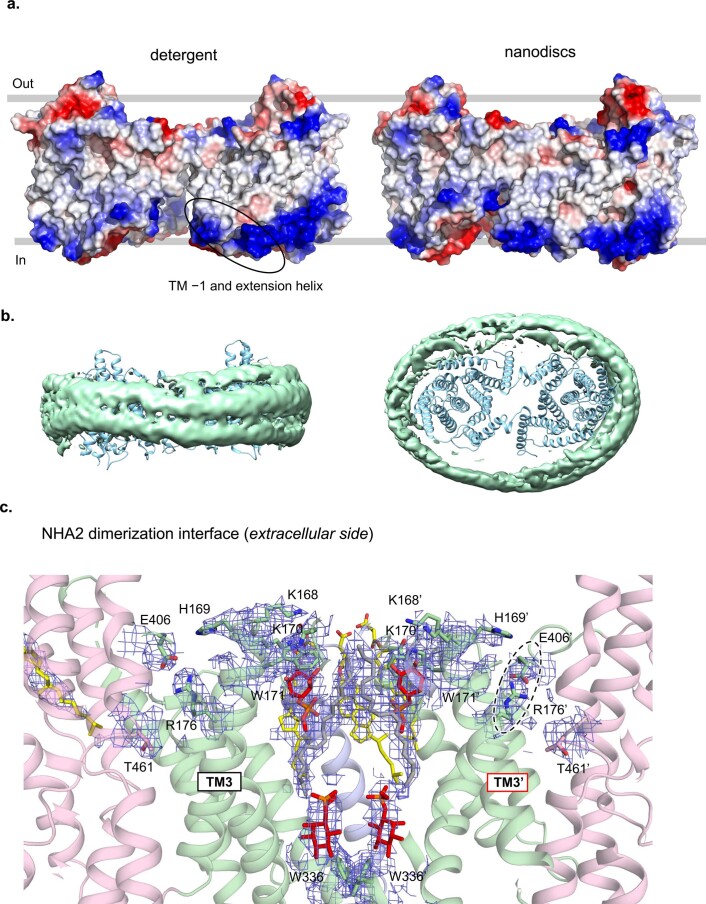
Comparison of *bison* NHA2 _ΔN_ structures in detergent and nanodiscs. **a**. Electrostatic surface representation of the side view of the outward-facing NHA2 homodimer highlighting the large, intracellular gap between protomers and intracellular positively-charged surface in detergent (*left*) that is closed upon TM –1 rearrangement in nanodiscs incorporated with PI lipids (*right*). **b**. The cryo EM density for the nanodisc surrounding NHA2_ΔN_ from the side and top, and the manually placed *bison* NHA2_ΔN_ structure determined in detergent. **c**. Cartoon representation on the dimer interface showing the polar contacts between the K168, H169, K170 and W171 residues, in the end of TM3 of one protomer (green sticks, labeled), and the identical residues from the neighboring protomer (green sticks, labeled with ‘), with the PI lipids located in between these residues at the center of the dimer interface. Also interacting with R176 of T3 is E406 (TM10), which might stabilize the outward-facing cavity leading the ion-binding, interaction encircled. Modelled lipids are shown as sticks for, PI lipid headgroups (red), cholesterol located on extracellular half of the protein (yellow), and PI tails (gray). Cryo-EM density is shown as blue mesh around the mentioned residues and ligands.

NHA2_ΔN_ was incorporated into nanodiscs containing pure yeast PI for structural determination. PIP_2_ was not trialed to avoid disrupting the homodimer at the high concentrations used in nanodisc preparations (Methods). Overall, the final cryo-EM map was estimated at 3.5 Å (FSC = 0.143 criterion), but with higher model resolution at 3.7 Å (FSC = 0.5) and uniform density of both protomers, reflecting the improved stability and map quality compared to the detergent NHA2_ΔN_ structure (Table 1, Fig. 3e, Extended Data Figs. 5a–c and 6 and Methods). Nevertheless, before masked refinement, we were unable to confidently model the N-terminal extension helix as it was difficult to distinguish between the respective nanodisc and protein densities. The only clear difference between the structure of NHA2_ΔN_ in detergent compared to nanodiscs was the positioning of TM −1, which had moved inwards by ~10 Å (Fig. 4a). Remarkably, the readjustment of TM −1 had completely closed the intracellular gap between protomers (Fig. 4b, Extended Data Fig. 7a and Supplementary Video 1a–c). Manual placement of the NHA2_ΔN_ detergent structure into the cryo-EM maps from nanodiscs confirms that there was additional space between the protein and the nanodisc (Extended Data Fig. 7b)—that is, indicating that the conformational difference was not simply caused by using too small nanodiscs. In the cryo-EM maps there are several additional densities at the dimerization interface next to tryptophan residues of equivalent signal strength (Fig. 4c and Extended Data Fig. 7c). Tryptophan is well known to be an important residue for anchoring TM segments at the lipid–water interface^37^. Owing to their size, shape and location, we interpret these densities as belonging to the sugar headgroup of four PI molecules, with some additional densities belonging to their fatty acid tails (Fig. 4b and Extended Data Fig. 7c).

On the extracellular side, two myo inositol headgroups interact with Trp171 in TM3 and possibly with Glu107 located in the TM −1 to TM1 loop, which is strictly conserved (Fig. 4c and Supplementary Fig. 2). This PI lipid site is the most likely position to accommodate PIP_2_ binding, because the indole nitrogen of Trp171, as well as the amine side chain groups of Lys168 and Lys170, could help coordinate the negatively charged phosphates (Fig. 4c). Stabilization of TM3 is further apparent by a salt bridge formed between Arg176 (TM3) and Glu406 (TM10) residues (Fig. 4c and Extended Data Fig. 7c). An Arg176Ala variant was well folded, but abolished Li^+^-complementation, whereas the His169Ala variant, located one turn away, retained growth to a similar level as NHA2_ΔN_ (Fig. 4c, Extended Data Fig. 7c and Supplementary Figs. 1 and 3c). Two strictly conserved tryptophan residues in TM −1 Trp97 and Trp101 further interact with several cholesterol lipids, which may also help to anchor the position of TM −1 on the extracellular side (Fig. 4c and Extended Data Fig. 7c). Although cholesterol hemisuccinate (CHS) was used throughout purification of NHA2_ΔN_, we find that cholesterol lipids are only clearly visible in the nanodisc NHA2 cryo-EM maps (Fig. 4c). On the cytoplasmic side, the inositol headgroup is positioned next to Trp336 from both protomers, which are located at the end of TM8 (Fig. 4c). Taken together, the structural data indicate that cholesterol and PI lipids anchor TM −1 on the extracellular side, enabling a conformational change to occur mainly on the cytoplasmic side. On the cytoplasmic side, TM −1 moves together with the DQ-motif connected to TM8, which has moved inwards at Trp336 to interact with PI lipids (Fig. 4c).

It is plausible that the NHA2_ΔN_ structure in detergent might represent a more extended conformation than that attained in a normal membrane bilayer. However, if NHA2 should only acquire the compacted homodimer seen in nanodiscs, it would not explain why the protein has evolved to oligomerize by the additional TM −1 helix. Dynamic elastic modeling of the nanodisc NHA2_ΔN_ structure shows that TM −1 is indeed flexible and can spontaneously adopt the position seen in the detergent structure (Supplementary Fig. 7a and Supplementary Video 2a). Intrinsic TM −1 mobility is facilitated by highly conserved proline and glycine residues located in the loop between TM −1 and TM1, and a cluster of five proline residues in the loop between TM1 and TM2 (Fig. 4a and Supplementary Fig. 2). Finally, the modeled N-terminal extension helix is also highly positively charged (Fig. 2d) and is reminiscent of the positively charged extension helix seen in the oligomeric betamine transporter BetP, which binds to negatively charged lipids in response to a change in cell volume^38^. As such, it possible that TM −1 mobility, oligomerization and elevator structural transitions might further be influenced by the extension helix (Supplementary Fig. 7b and Supplementary Video 2b), leading to the question of whether NHA2_ΔN_ might have a role in volume sensing, as seen in other NHE proteins^2^.

### NHA2 extracellular funnel and the ion-binding site

**Fig. 5:**
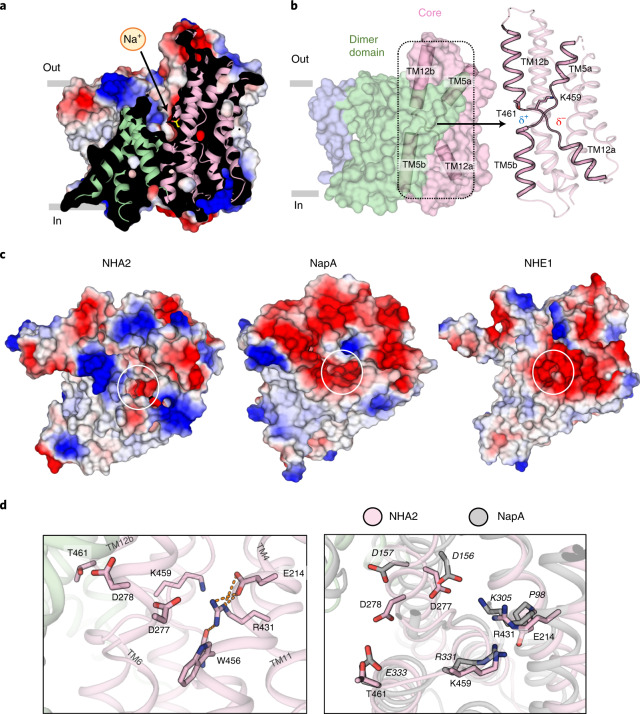
NHA2_ΔN_ ion-binding site and its electroneutral activity. **a**, Cartoon representation of NHA2_ΔN_ in nanodiscs with the electrostatic surface representation through the ion-binding site of one monomer (colored blue to red, for positive to negative charge). The strictly conserved ion-binding residue Asp278 is labeled and shown in yellow sticks. **b**, Surface representation of NHA2_ΔN_ in nanodiscs, showing the 6-TM core domain (pink), the dimerization domain (green) and the N-terminal domain-swapped transmembrane helix TM −1 (blue). The crossover of half helices TM5a-b and TM12a-b (cartoon) are unique to the NhaA-fold and the half-helical dipoles that they create are highlighted. In NHA2_ΔN_, a lysine residue (K459 in stick form) is well positioned to neutralize the negatively charged half-helical dipoles, but the positively charged dipoles lack a negatively charged residue that is conserved in all other Na^+^/H^+^ antiporter structures^19^. Instead, the polar residue (T461 in stick form) that is conserved in all NHA2 members is enough for protein stability (Extended Data Fig. 1). **c**, Electrostatic surface representation of the extracellular view of the monomers of outward-facing NHA2 (left), outward-facing NapA (middle; PDB 4BWZ) and outward-facing human NHE1 (right; PDB 7DSX). The circles show the positions of the respective outward-facing funnels. **d**, Left: the ion-binding site of NHA2 has the two aspartates seen in electrogenic Na^+^/H^+^ antiporters (D277 and D278, in stick form) and a NHA2-specific salt bridge between R431 and E214 that is connected to W456 (residues shown in stick form). Dashed lines represent hydrogen bonding. Right: ion-binding site comparison between NHA2 (pink sticks) and electrogenic NapA (gray sticks and residue number in italic), highlighting the differences in E214 (P98 in NapA) and T461 (E333 in NapA).

**Extended Data Fig. 8:**
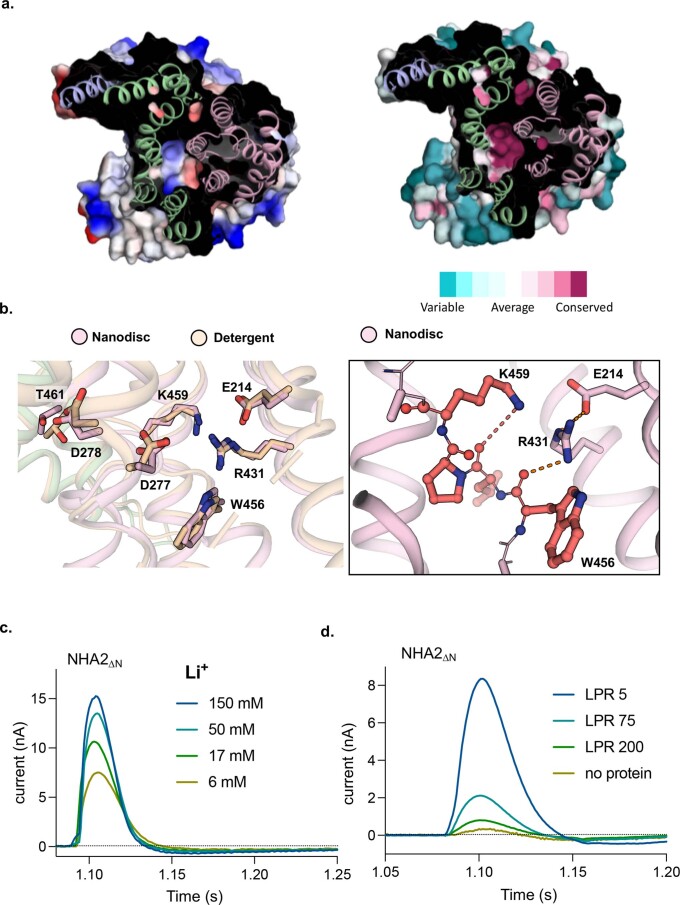
Ion-binding site and SSM measurements of *bison* NHA2 _ΔN_. **a**. *left:* electrostatic surface of the NHA2_ΔN_ ion-binding site from the detergent structure. Model is shown in cartoon representation colored as in Fig. 1c, the electrostatic surface potential is colored blue to red, for positive to negative charges, respectively. *right:* as in the left panel, but surface shows residue conservation, colored according to conservation scores from the alignment of representative 500 mammalian NHA2 sequences calculated usning the ConSurf server^83^ to highlight that the negative charges on one side of the outward-facing funnel are highly conserved. **b** *left:* Comparison between ion-binding site residues in the NHA2_ΔN_ structure obtained in detergent and nanodiscs, with helices shown in cartoon representation and residues as sticks colored for nanodisc as in Fig. 1c, and detergent in sand. *right:* Cartoon representation of the ion-binding site of NHA2 in nanodiscs with the residues of the TM12a-b breakpoint illustrated in stick form to highlight the stabilization by K459 and R431, which is further salt-bridged to E214, with putative hydrogen bonds illustrated as dotted lines, as is the salt bridge. **c**. SSM-based electrophysiology measurements of *bison* NHA2_ΔN_ proteoliposome with transient currents recorded after Li^+^ concentration jumps at pH 7.5 on both sides. **d**. As in c. Transient currents in different lipid to protein ratios as indicated. Source data

The core domain in NHA2_ΔN_ is in the outward-facing conformation (Fig. 5a). The characteristic feature of the ‘NhaA-fold’ is the two discontinuous helices TM5a-b and TM12a-b in the core domain, which contain unwound regions that cross over each other near the center of the membrane (Fig. 5b)^22^. Proximal to these extended helix break points are typically oppositely charged residues, which neutralize the half-helical termini dipoles of TM5a and TM12b^19^. In NHA2_ΔN_, however, Thr461 replaces the conventional negatively charged aspartate or glutamate residue and, on the other side, Lys459 replaces the arginine residue used in all 13-TM members^19,23–27^ (Fig. 5b). The substitution of Thr461 to alanine remained functional in *S. cerevisiae* complementation assays, whereas either Thr461-to-glutamic acid or Lys459-to-arginine mutants were both non-functional under high Li^+^-salt stress, indicating that these amino acid differences have evolved to be optimal for NHA2 function (Extended Data Fig. 1c and Supplementary Figs. 1 and 3d). The absence of acidic residues in both the helix-break positions and in the funnel itself gives rise to an outward-facing cavity that lacks the extensive negatively charged surfaces seen in other outward-facing Na^+^/H^+^ exchanger structures (Fig. 5a–c and Extended Data Fig. 8a)^24,25^.

**Fig. 6:**
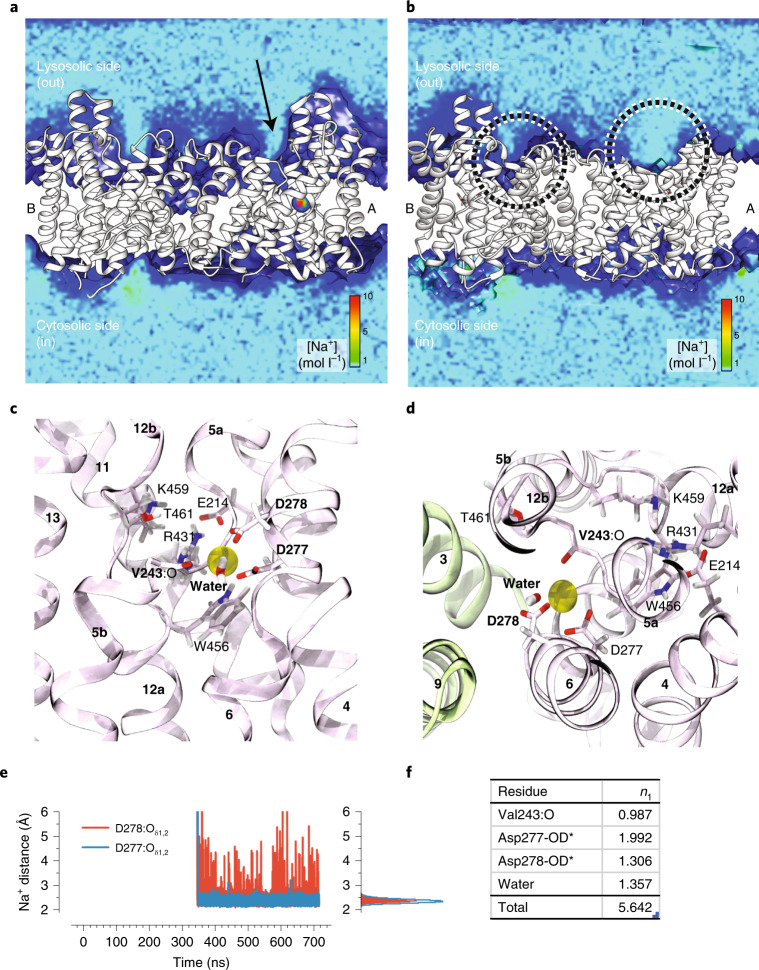
MD simulations of ion-binding to NHA2_ΔN_. **a**, Density of sodium ions over 716 ns of simulation f-01-0 during which one sodium ion spontaneously entered the binding site in protomer A. The [NaCl] bulk concentration was 150 mM and ions were free to diffuse. At the start of the simulation, both D277 and D278 were deprotonated in protomer A (right). Protomer B (left) had D277 protonated (neutral), while D278 remained in the carboxylate form (negatively charged). The arrow indicates the outward-facing entrance funnel through which ions diffuse to the binding site. The NHA2 dimer, shown in side view along the membrane, is presented in cartoon representation colored white; the membrane was omitted when preparing the figure for clarity. **b**, Sodium ion density, as in **a**, but when either Asp277 is deprotonated and Asp278 is protonated (protomer A, left) or both aspartates are protonated (protomer B, right) (based on simulation f-23-1). Ions do not enter the lysosolic funnel regions (highlighted with dashed circles). **c**, Side view from the dimerization domain (omitted) on the putative sodium-binding site, drawn from the last frame of the MD simulation. The sodium ion is shown as a yellow sphere, sodium-coordinating residues are indicated in bold, and other residues near the binding site are labeled for context. Water molecules within 3 Å of the sodium ion were included, with only one present in this snapshot at the end of the MD trajectory. **d**, Top view (from the lysosolic side), with elements as in **c**, with the addition of the dimer domain helices in light green. **e**, The shortest distance of any sodium ion to any carboxylate oxygen in either D277 or D278: time series (left) and histogram (right). **f**, Coordination of the bound sodium ion. The average contributions of oxygen atoms from different residues to the first hydration shell of the bound sodium ion, *n*_1_, identify the binding site residues. ‘OD*’ indicates the additive contributions from both the O_δ1_ and O_δ2_ carboxylate oxygen atoms; ‘water’ from any water molecules. *n*_1_ < 0.001 are not shown.

The ion-binding-site aspartates Asp277 and Asp278 are strictly conserved and, due to a lack of experimental map density, the side chain rotamers were modeled based on the equivalent positions of the corresponding well-defined ion-binding aspartates from NapA (Fig. 5d and Supplementary Fig. 2). In molecular dynamics (MD) simulations of the nanodisc NHA2_ΔN_ structure, a potential sodium-ion-binding event was captured that involved Asp277, Asp278, water molecules and the backbone carbonyl oxygen atom of Val243 in the broken stretch of TM5a-b (Fig. 6 and Supplementary Fig. 8a,b). In the simulations, the side chains of Asp278 and Asp277 easily adapt to the presence of a sodium ion by switching to a different rotamer. Consistent with there being few negatively charged residues located in the funnel, only a few Na^+^ ions spontaneously reach the ion-binding Asp277 and Asp278 residues (Fig. 6). During a total simulation time of 1.5 µs, only one spontaneous binding event and one partial binding event were observed (Supplementary Fig. 8c–f and Table 1), whereas in MD simulations of outward-facing NapA, tens of events were recorded over a similar time period^29^. Moreover, in simulations with either of the aspartate residues, or both, protonated (charge-neutral) sodium ions did not diffuse into the funnel (Fig. 6), suggesting that sodium ion binding requires both aspartates to be negatively charged to provide an electrostatically favorable environment. In contrast to the extracellular surface, the cytoplasmic surface of the core domain in NHA2_ΔN_ is highly negatively charged (Fig. 2d), which indicates that NHA2_ΔN_ might have a preferential mode of Na^+^ uptake from the cytoplasmic side. The extracellular half-helices TM5a and TM12b in NHA2 have also rotated and moved closer to the dimer domain than their comparable position in the outward-facing structure of NapA (Extended Data Fig. 4b). The rearrangements of these flexible half-helices are in agreement with the mobility of TM12b seen previously between different outward-facing structures of NapA and could represent local gating differences^29^.

### NHA2 activity is electroneutral despite two aspartates

Based on sequence conservation, mutational analysis and the monomeric crystal structure of NhaA, both ion-binding aspartates were initially thought to be the two proton carriers conveying electrogenicity^20,22^. However, subsequent NhaA^39^ and NapA^24^ crystal structures in combination with functional analysis^40^ and MD simulations^41^ have proposed that a lysine residue forming a salt bridge to one of the aspartate residues is the second proton carrier^39,41^. In NHA2_ΔN_, Asp277 is located ~5 Å from the arginine residue Arg431, rather than the salt-bridge-forming lysine residue found in the electrogenic NhaA^39^ and NapA^24^ members (Fig. 5c and Extended Data Fig. 8b). Because of the distance of Arg431 from Asp277 and the high p*K*_a_ of arginine^42^, it is unlikely that Arg431 could act as a proton carrier in NHA2. Consistent with this structural interpretation, NHA2 activity is thought to be electroneutral, as transport is unaffected by the collapse of the membrane potential in cells, oocytes and proteoliposomes^12,18,40^.

**Fig. 7:**
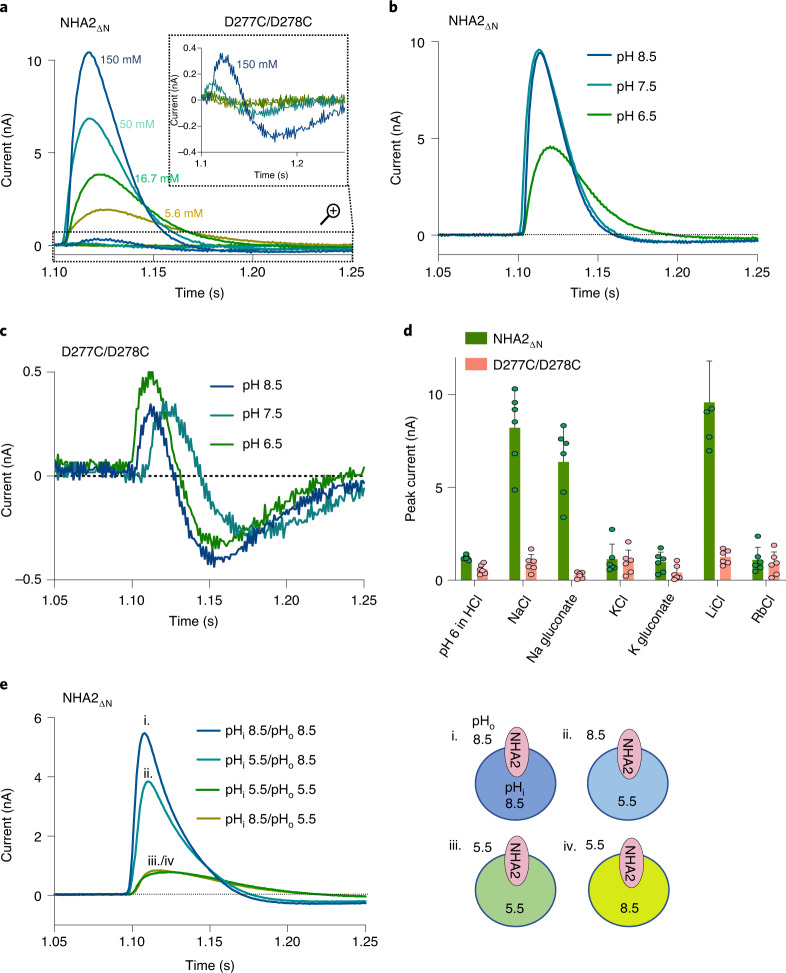
SSM-based electrophysiology measurements of bison NHA2_ΔN_ proteoliposomes. **a**, Transient currents recorded on NHA2_ΔN_ proteoliposomes under symmetrical pH 7.5 and increasing Na^+^ concentration jumps as shown. Inset: zoomed-in responses to NHA2_ΔN_ in which the ion-binding aspartates were substituted to cysteine (D277C–D278C). **b**, Transient currents recorded after addition of 150 mM NaCl at symmetrical pH 6.5, 7.5 and 8.5 for bison NHA2_ΔN_. **c**, As in **b** for the bison NHA2_ΔN_ construct in which Asp278 and Asp279 were substituted with cysteine. **d**, Peak current averages in response to different concentration jumps. The first bars show peak currents obtained by pH jumps from pH 7.0 to pH 6.0 titrated with HCl. The following bars show peak currents upon exchange of 150 mM choline chloride with 150 mM of the given salt at pH 7.5. Error bars show the mean values ± s.d. of *n* = 6 independent experiments (sensors). **e**, The pH was varied independently inside (pH_i_) and outside (pH_o_) the proteoliposomes, followed by 150 mM NaCl jumps to activate NHA2 in the presence of a pH gradient. Only a pH reduction on the outside dramatically affects current amplitudes, which is consistent with an electroneutral transport cycle^43,44^. Representative results of recordings performed on two individual sensors are shown. Source data

**Extended Data Fig. 9:**
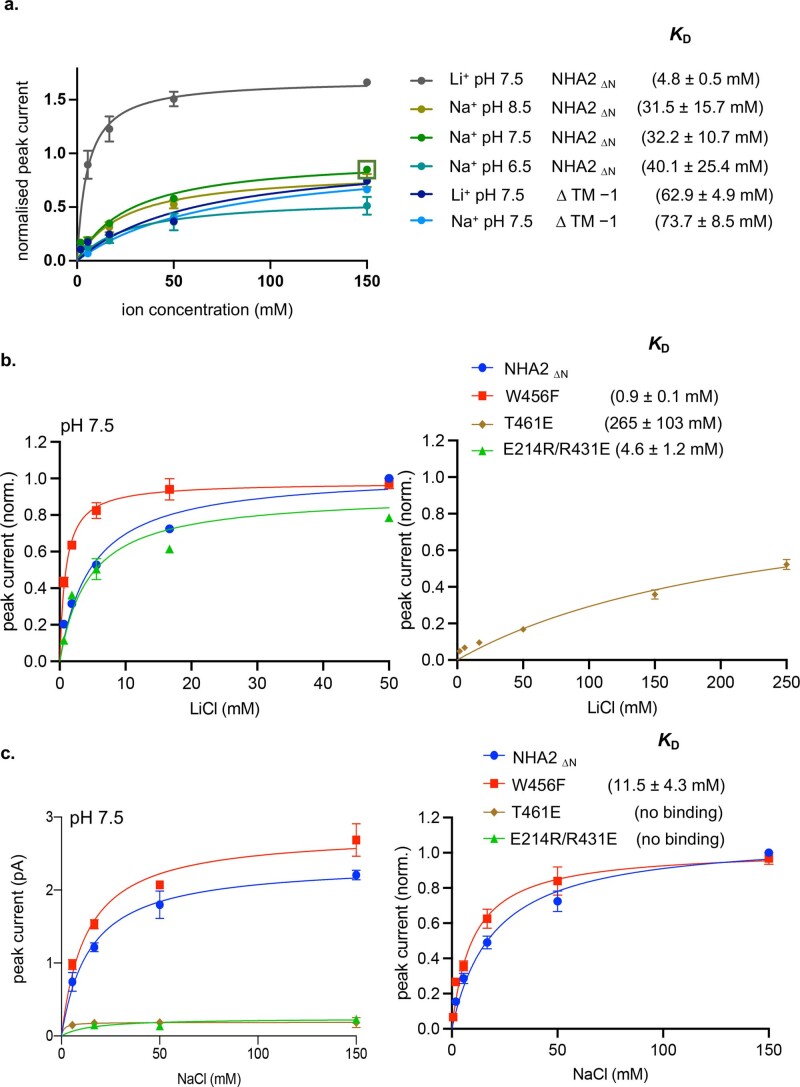
SSM-based electrophysiology measurements of NHA2 _ΔN_ and variants in proteoliposomes. **a**. Fit of the normalized amplitude of the transient currents as a function of Na^+^ (Li^+^) concentrations and pH for *bison* NHA2_ΔN_ and *bison* NHA2_ΔTM–1_ and the corresponding binding affinity (*K*_D_). Note, since we are measuring pre-steady-state ion translocation rather than steady-state currents, it is more accurate to refer this estimate as a binding constant (K_D_) rather than the Michaelis–Menten constant, *K*_M_. Currents have been normalized for *bison* NHA2_ΔN_ 150 mM Na^+^ at pH 7.5 as indicated by the green square. Error bars are the mean values ± s.d. of: n = 6 independent experiments (sensors) for *bison* NHA2_ΔN_ with NaCl; n = 7 independent experiments (sensors) for *bison* NHA2_ΔTM-1_ with NaCl; n = 3 independent experiments (sensors) for *bison* NHA2_ΔN_ and *bison* NHA2_ΔN_ with LiCl **b**. The fit of the normalized amplitudes for *bison* NHA2_ΔN_ and derived variants W456F and E214R-R431E recorded after Li^+^ concentration jumps at symmetrical pH 7.5 (*left*) and for the variant T461E (*right*). Error bars are the mean values ± s.d. of n = 3 independent experiments (sensors) **c**. As in b. for NaCl additions. Error bars are the mean values ± s.d. of n = 3 independent experiments (sensors). Note, the either the weak or no binding observed for the T461E variant with Li^+^ and Na^+^ addition respectively, is consistent with the lack of complementation to Li^+^ or Na^+^ salt-stress in the AB11c strain (Supplementary Fig. 3d-e). Source data

To robustly confirm the electroneutral activity of NHA2, the protein was reconstituted into liposomes for solid-supported membrane (SSM)-based electrophysiology recordings, which is a more sensitive technique than patch-clamped electrophysiology for low-turnover transporters^43^. With this technique, proteoliposomes are adsorbed to an SSM and charge translocation of ions is measured via capacitive coupling of the supporting membrane^43^. Importantly, SSM-based electrophysiology can also detect pre-steady-state currents of the half-reaction in electroneutral transporters, that is, the binding and transport of Na^+^ across the membrane, as Na^+^ accumulates at a much faster rate than the counter H^+^ efflux in the absence of ΔpH (ref. ^44^). Peak currents at pH 7.5 were observed for NHA2_ΔN_ with increasing concentrations of either Na^+^ or Li^+^ that were 20 to 50 times higher than the signal obtained from the transport-inactive Asp277Cys-Asp278Cys double mutant (Fig. 7a and Extended Data Fig. 8c). Consistent with the actual NHA2_ΔN_ functional activity, currents were pH-dependent and proportional to the lipid–protein ratio (LPR), whereas the ion-binding aspartate mutant showed no differences from the signals obtained from empty liposomes (Fig. 7a–c and Extended Data Fig. 8d). Replacing NaCl with Na^+^-glucoronate showed similar peak currents, and either KCl or K^+^-glucoronate addition showed no activity (Fig. 7d), ruling out that NHA2 might transport either K^+^ or Cl^−^ ions as proposed in the closely related isoform, NHA1^18^. The peak currents recorded were pre-steady state and their positive amplitudes are entirely consistent with electroneutral transport^44^, rather than electrogenic signals, which would show amplitudes in the opposite direction^43^. Moreover, as expected for pre-steady-state currents, NHA2 activity was substantially more diminished when the pH was reduced on the outside as compared to the inside of the liposomes (Fig. 7e); that is, H^+^ on the outside competes with Na^+^ (Li^+^) for the same ion-binding site and lowers the rate of Na^+^ (Li^+^) translocation. The ∼30% reduction of transient Na^+^ currents when the pH is decreased on the inside rather than on the outside occurs because the outwardly directed ΔpH probably accelerates H^+^ translocation, which dampens the signal recorded from Na^+^ influx (Fig. 7e). The apparent binding affinities of NHA2_ΔN_ for Na^+^ (*K*_D_ = 32 mM) and Li^+^ (*K*_D_ = 4.8 mM) at pH 7.5 were determined (Extended Data Fig. 9a)^40^ and are comparable to the affinity (*K*_M_) for extracellular Na^+^ in most NHEs^2^. Notably, we estimate a biased ~75% inside-out orientation for protein reconstitution, which means the *K*_D_ estimate more closely reflects the physiological situation (Methods and Supplementary Fig. 9). Given that NHA2 is unable to bind Na^+^ at pH 4.6, the ion affinities are consistent with an inwardly directed pH gradient driving the export of Na^+^ from the neutral pH of the cytoplasm. Taken together, the structure and SSM-based electrophysiology data confirm that NHA2 operates as an electroneutral transporter to drive Na^+^ efflux.

In addition to Arg431 being distant from Asp277, the guanidinium group of Arg431 is also forming salt-bridge interactions with a nearby glutamate Glu214 in TM4 and a backbone carbonyl oxygen located at the TM12a-b breakpoint (Fig. 5d). The TM12a-b breakpoint is further stabilized by interactions with Lys459 (Extended Data Fig. 8a). Bioinformatic analysis has previously shown that the Glu214 residue is unique to the NHA2 members^19^, and residue substitutions lacking a carboxylate failed to rescue the salt-sensitive phenotype of the host strain^45^. Consistently, alanine mutations of either Glu214, Arg431 or Lys459 severely affected complementation under Li^+^-salt stress (Extended Data Fig. 1c and Supplementary Figs. 1 and 3d). To further investigate the importance of the NHA2-specific salt bridge between the Arg431 and Glu214 residues, single Glu214Arg and Arg431Glu mutants were generated and, as expected, showed either no or poor complementation under Li^+^-salt stress, respectively (Extended Data Fig. 1c and Supplementary Fig. 3d). Size-exclusion profiles demonstrated that the Arg431Glu mutant was well tolerated, but Glu214Arg was poorly folded, indicating that Lys459 might be able to compensate and maintain stability in the Arg431Glu mutant (Supplementary Fig. 1a,b). Interestingly, the salt bridge swapped mutant Glu214Arg-Arg431Glu, was able to rescue Li^+^-salt stress to a similar degree as NHA2_ΔN_, but could not complement for Na^+^ (Extended Data Fig. 1c and Supplementary Figs. 1 and 3d). SSM-based electrophysiology confirms that this mutant has now converted NHA2_ΔN_ into a Li^+^-specific transporter, as the *K*_D_ for the mutant remains the same for Li^+^ (*K*_D_ = 4.6 mM), but Na^+^ addition showed no measurable binding (Extended Data Fig. 9b,c). These results provide strong evidence that the salt-bridge pairing between Glu214 (TM4) and Arg431 (TM11) is mainly structural, but their environment can clearly fine-tune cation specificity.

The presence of the bulky amino acid tryptophan (Trp456) at the end of TM12a is quite distinct from all other Na^+^/H^+^ exchanger structures that, like NapA, have smaller, non-aromatic side chain residues in this position (Fig. 5d). The mutation of Trp456 to either phenylalanine or alanine retained some complementation for Na^+^ and Li^+^ like NHA2_ΔN_, but not under high salt stress (Supplementary Figs. 1a,b and 3d,e). SSM-based electrophysiology showed that the Trp456Phe mutant has, in fact, higher Li^+^ and Na^+^ apparent affinities to NHA2_ΔN_, with a fivefold increase for Li^+^ (*K*_D_ = 0.9 mM), yet it is unclear whether this results in a slower turnover (Extended Data Fig. 9b). Notably, Trp456 in the breakpoint between TM12a-b is reminiscent of the *π* bulges in antiporter-like subunits of complex I, which form *π*-cation interactions to a nearby lysine residue^46^. It is plausible that, in a Na^+^-bound conformation, Tp456 forms *π*-cation interactions to arginine, which may help to stabilize Na^+^ coordination. Clearly, an ion-bound structure is required to piece together how Trp456 is ultimately coupled to this unique salt-bridge network (Extended Data Fig. 8a).

## Discussion

**Extended Data Fig. 10:**
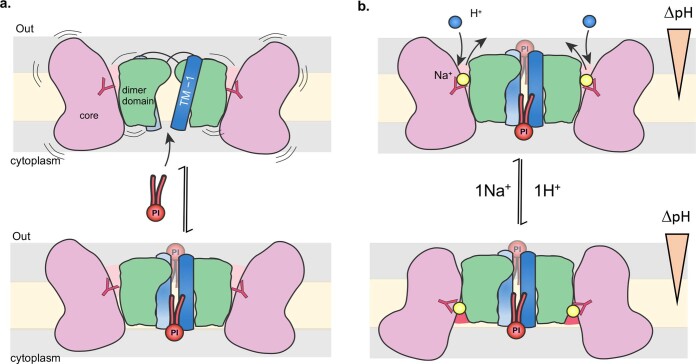
Schematic summary of NHA2 lipid-remodeling and electroneutral exchange. **a**. In the presence of PI lipids NHA2 undergoes a conformational change, closing the intracellular gap between protomers to form a more compacted homodimer, which is likely the more active state of the protein; core transport domain (pink), dimer domain (green), additional dimer domain-swapped helix (TM –1 in blue), PI lipid (red) and ion-binding aspartate (red stick). **b**. NHA2 co-localises with an V-type H^+^-ATPase and the proton (blue-sphere) gradient is used to drive the efflux of sodium (yellow-sphere) from the cytoplasm, with a 1H^+^: 1Na^+^ stoichiometry (and a Na^+^ binding affinity K_D_ = ~30 mM) into either organelles, for example, lysosomes or across the plasma membrane of specialized cells (components colored as in a).

The cryo-EM structure of NHA2 reveals the closest structural similarity to the bacterial electrogenic Na^+^/H^+^ antiporter NapA^24^, but with an additional N-terminal helix (TM −1) that creates a unique, homodimer assembly. In NHA2, this additional N-terminal helix mediating oligomerization creates a large, cytoplasmic gap between protomers, which is intrinsically dynamic and can be closed by PI lipids (Extended Data Fig. 10a). Thermal-shift assays, native MS and judicial placement of tryptophan residues are consistent with the observed PI-lipid-mediated remodeling on the cytoplasmic side. Although we cannot exclude that other types of lipid may influence the lipid remodeling of NHA2, phosphoinositol lipids are certainly the most common lipid type for ion-channel and transporter regulation^47^. Moreover, in crude membrane fractions from liver, brain and yeast, the latter was clearly the most thermostabilizing and is the only crude lipid fraction with a high PI content. Interestingly, PI lipids are highly enriched in intracellular organelles^48^, which is the preferential localization of NHA2. However, given that NHA2 can also localize to the plasma membrane of specialized cells^8^, it is currently unclear whether PIP_2_ can play a similar role to PI in these membranes. The predicted PIP_2_ sites in the extracellular side of NHA2 also need further validation because PIP_2_ is principally found in the inner leaflet of the plasma membrane, but not exclusively^49^.

In *E. coli* NhaA, the positively charged dimer interface has evolved to bind the negatively charged lipid cardiolipin, which has been shown to be required for homodimerization^36,50^ and also in vivo functional activity^51^. In *E. coli*, salt stress increases the synthesis of cardiolipin, and NhaA is required to cope with salt stress^51^. The negatively charged lipids PIP_2,3_ also stabilize NHE9 via a unique loop domain located at the dimerization interface^23^. Overall, there is a precedence of lipids influencing homodimerization of Na^+^/H^+^ exchangers, which gives further support to the lipid remodeling observed here for NHA2. From a structural viewpoint, the additional TM −1 helix in NHA2 expands the structural inverted repeat from 5-TMs (NhaA) to 6-TMs (NapA and other 13-TM members), to now 7-TMs in NHA2 (Extended Data Fig. 4a). The expansion in the number of TM segments clearly alters how the Na^+^/H^+^ exchangers dimerize, implying, from a structural context, that oligomerization has probably evolved to enable differences in how their activities can be regulated by homodimerization.

We have shown that oligomerization is essential for NHA2 activity, and the reasonable assumption is that the lipid-compacted form is more active, because it provides a more stable anchor for core domain elevator transitions. More recently, it has been shown that the addition of cardiolipin to NhaA increases the affinity of ^22^Na^+^ binding^52^. It is also possible that stabilization of the NHA2 homodimer has a regulatory role that is also linked to ion binding. Consistent with this rationale, TM3 was stabilized in the nanodisc structure by both PI lipids and a salt-bridge interaction, which is important, because the dimer domain helix TM3 is positioned opposite the ion-binding site and forms the hydrophobic barrier across which the core domain moves^23^. Interestingly, SSM-based electrophysiology showed that the Na^+^ and Li^+^ binding affinities (*K*_D_) for the monomeric NHA2_ΔTM−1_ construct were 2- and 13-fold poorer, respectively, than NHA2_ΔN_ (Extended Data Fig. 9a).

Despite NHA2 containing two aspartate residues in the ion-binding site, SSM-based electrophysiology confirms earlier studies^12,18,40^ showing that NHA2 performs electroneutral rather than electrogenic transport (Extended Data Fig. 8). From a mechanistic standpoint, this conclusion supports that lysine, rather than aspartate, is the second proton carrier in bacterial electrogenic Na^+^/H^+^ antiporters^40^. From an energetics perspective, this means that NHA2 activity will only be driven by ΔH^+^ or ΔNa^+^ gradients. As such, proton-driven Na^+^ efflux in mammalian cells will require a steep proton gradient, which is consistent with the co-localization of NHA2 with the V-type H^+^-ATPase^12,13^ (Extended Data Fig. 10b). Given the low number of spontaneous Na^+^ binding events seen in MD simulations in the outward-facing state, NHA2 may have an in-built preference for Na^+^ uptake on the cytoplasmic side, which would be more consistent with the apparent affinity estimates. Moreover, endosomal and lysosomal NHE6 and NHE9 transporters have an extensive C-terminal tail that binds several extrinsic factors to regulate their transport activity^2^. By contrast, NHA2 lacks this regulatory domain and, as such, one might expect NHA2 to have more of a housekeeping role in these organelles.

Finally, Na^+^-dependent amino acid transporters of the SLC38 family are crucial for the regulation of mTOR complex 1 (mTORC1)^53^ in lysosomes, and it remains to be investigated how NHA2 activities influence lysosomal ion homeostasis. Lysosome volumes and Na^+^ concentrations can change significantly^54^, and future studies are required to establish whether the lipid-remodeling of NHA2 and its activities can be triggered by a change in cell or organellar volume, which alters the membrane thickness, and cholesterol and PIP_2_ distributions. Indeed, tryptophan is a well-established sensor that can respond to changes in hydrophobic mismatch by changing its position to rearrange the tilt angle of helices^37^. Although many questions remain unanswered, the NHA2 structure and lipid remodeling offer a new molecular framework for exploring these new avenues, with interesting physiological ramifications.

## Methods

### NHA2 structural selection using fluorescence-based screening

NHA2 genes from *Homo sapiens* (human), *Mus musculus*, *Bos taurus* and *Bison bison* were synthesized and cloned into the GAL1-inducible *Tobacco etch virus* (TEV) site containing GFP-TwinStrep-His_8_ vector pDDGFP3, and transformed into *S. cerevisiae* strain FGY217 (MATα, ura3–52, lys2Δ201 and pep4Δ) as previously described^55,56^. The highest yielding constructs were overexpressed in 2-l cultures, cells were collected and membranes isolated and solubilized with 1% *n*-dodecyl-β-d-maltoside (DDM, Glycon). Subsequently, the monodispersity of the detergent-solubilized protein product was assessed by fluorescence-detection size-exclusion chromatography (FSEC) using a Shimadzu HPLC LC-20AD/RF-20A (488 nm_ex_, 512 nm_em_) instrument and a Superose 6 10/300 column (GE Healthcare) in 20 mM Tris-HCl, pH 7.5, 150 mM NaCl and 0.03% DDM buffer. The respective thermostability of the highest expressing and most monodisperse candidate constructs were determined as previously described^36,56,57^.

All NHA2 variants were generated with a standard polymerase chain reaction (PCR)-based strategy and the bison NHA2_ΔTM_ sequence (truncated 1–69 residues in italics) was as follows (TEV cleavage sequence underlined):

*MRNQDKRAAHKDSEPSTEVNHTASSYQGRQQETGMNLRGIDGNEPTEGSNLLNNNEKMQGTPAEPNHLQ*RRRQIHACPPRGLLARVITNVTMVILLWAVVWSVTGSECLPGGNLFGIIMLFYCAIIGGKLFGLIKLPTLPPLPPLLGMLLAGFLIRNVPVISDNIQIKHKWSSALRSIALSVILVRAGLGLDSNALKKLKGVCVRLSLGPCLIEACTSAVLAYFLMGLPWQWGFMLGFVLGAVSPAVVVPSMLLLQEGGYGVEKGIPTLLMAAGSFDDILAITGFNTCLGMAFSTGSTVFNVLKGVLEVIIGVVTGLVLGFFIQYFPSSDQDNLVWKRAFLVLGLSVLAVFSSTYFGFPGSGGLCTLVTAFLAGRGWASTKTDVEKVIAVAWDIFQPLLFGLIGAEVLITALRPETIGLCVATLGIAVLIRILVTYLMVCFAGFNIKEKIFISFAWLPKATVQAAIGSVALDTARSHGEKQLEGYGMDVLTVAFLSIIITAPVGSLLIGLLGPRLLQKAEQNKDEEDQGETSIQVENLYQFG

### Complementation assay for mammalian NHA2 against salt stress in yeast

*S. cerevisiae* strain AB11c (*ena1-4Δnhx1Δnha1Δ*) lacking endogenous Na^+^(Li^+^) efflux proteins^32^ was used as a host for the expression of bison and human NHA2 and constructs. To confirm the expression and proper folding of NHA2 constructs in the AB11c strain, heterologous expression conditions were repeated as previously described for the FGY217 strain and membranes were isolated for FSEC analysis^55^. In brief, cells were cultivated in 10 ml of culture in 50-ml aerated capped tubes containing medium lacking uracil (-URA) at 30 °C and 150 r.p.m. At an optical density at 600 nm (OD_600_) of 0.6 a.u., protein overexpression was induced by the addition of galactose to a final concentration of 2% (wt/vol) and incubation was continued at 30 °C and 150 r.p.m. The cells were collected after 22 h by centrifugation (2,250*g*, 4 °C, 5 min), resuspended in cell resuspension buffer (yeast suspension buffer ;YSB, 50 mM Tris-HCl (pH 7.6), 5 mM EDTA, 10% glycerol and 1× complete EDTA-free protease inhibitor cocktail (Roche). To estimate the NHA2 expression levels in whole cells, the cell suspension was transferred to an optical-bottom 96-well plate and GFP fluorescence emission was measured at 512 nm by excitation at 488 nm using a Fluoroskan plate reader (Thermo Scientific) as previously described^55^. To assess the localization of the NHA2-GFP fusions, an aliquot of cell suspension was viewed with a Zeiss LSM700 scanning confocal microscope as previously described^55^. A KDEL3-GFP fusion was also monitored as an endoplasmic reticulum marker. The cells were subsequently lysed by bead-beating (Sigma) on a table-top vortex for 5 min. Cell debris was removed and the resulting supernatant membranes were isolated by ultracentrifugation (120,000*g*, 4 °C, 1 h) and resuspended in YSB. The membranes were solubilized by the addition of DDM to a final concentration of 1% (wt/vol). Subsequently, the monodispersity of the detergent-solubilized protein product was assessed by FSEC^57,58^ as described for the NHA2 structural selection using fluorescence-based screening.

Saturated seed cultures were diluted to a starting OD_600_ value of either 0.2 (for Li^+^ toxicity testing) or 0.02 (for Na^+^ toxicity testing) in -URA medium containing 2% (wt/vol) galactose and different concentrations of either LiCl or NaCl to a final volume of 200 μl in a 96-well plate, and cultured at 30 °C. After incubation at 30 °C for 48–72 h, the cultures were resuspended and the OD_600_ recorded using Spectramax plate reader (Molecular Devices).

#### NHA2 purification

*S. cerevisiae* strain FGY217 was transformed with the respective vector and cultivated in 12l cultures containing -URA medium and 0.1% d-glucose and shaken at 30 °C and 150 r.p.m. in Tunair flasks (Merck) using Innova 44R incubators (New Brunswick). At an OD_600_ of 0.6, protein overexpression was induced by the addition of galactose to a final concentration of 2% (wt/vol) and incubation was continued at 30 °C and 150 r.p.m. Cells were collected after 22 h by centrifugation (9,000*g*, 4 °C, 10 min), resuspended in buffer (50 mM Tris-HCl pH 8.0, 1 mM EDTA, 0.6 M sorbitol) and subsequently lysed by mechanical disruption as previously described^55^. Cell debris were removed by centrifugation (3,500*g*, 4 °C, 20 min) and, from the resulting supernatant, membranes were isolated by ultracentrifugation (100,000*g*, 4 °C, 1 h) and homogenized in membrane resuspension buffer (20 mM Tris-HCl pH 7.5, 0.3 M sucrose).

For samples used for structural studies in detergent with cryo-EM, membranes were solubilized in solubilization buffer (1% (wt/vol) DDM (Glycon), 0.2% (wt/vol) CHS (Sigma-Aldrich), 20 mM Tris-HCl pH 8.0, 150 mM NaCl) during mild agitation for 1 h at 4 °C. Subsequently, non-solubilized material was removed by ultracentrifugation (100,000*g*, 4 °C, 1 h). The supernatant was incubated for 2 h at 4 °C with 5 ml of Ni-NTA agarose (Qiagen) pre-equilibrated in wash buffer 1 (WB1, 0.1% (wt/vol) DDM, 0.02% (wt/vol) CHS, 50 mM Tris-HCl pH 8.0, 300 mM NaCl and 20 mM imidazole). This resin was transferred into a gravity flow column (Bio-Rad) and subsequently washed with 300 ml WB1. For the elution buffer (EB), 0.1% (wt/vol) DDM, 0.02% (wt/vol) CHS, 50 mM Tris-HCl pH 8.0, 300 mM NaCl and 300 mM imidazole were used. The eluted protein was then incubated overnight at 4 °C in the presence of equimolar amounts of TEV protease with mild agitation. The digested protein was collected, concentrated using 100-kDa molecular-weight cutoff spin concentrators (Amicon Merck-Millipore) and subjected to SEC using a Superose 6 Increase 10/300 column (GE Healthcare) and an Äkta Purifier FPLC system (GE Healthcare) in 20 mM Tris-HCl pH 7.5, 150 mM NaCl, 0.001% (wt/vol) lauryl maltose neopentyl glycol (Anatrace) and 0.0002% (wt/vol) CHS.

Samples used for SSM-based electrophysiology studies were purified as described above with the following alterations. (1) The solubilization buffer used was 20 mM Tris-HCl pH 8.0, 150 mM NaCl, 1% (wt/vol) DDM, 0.2% (wt/vol) CHS and 10% glycerol. (2) The protein was eluted in 20 mM Tris-HCl pH 8.0, 150 mM NaCl, 0.03% (wt/vol) DDM, 0.006% (wt/vol) CHS and 250 mM imidazole, and TEV digestion was performed during dialysis against imidazole free buffer overnight. (3) Before SEC, an additional reverse His-binding step was performed using a 5-ml HisTrap FF column (Cytiva) to remove non-digested protein and the still His-tagged TEV protease. (4) SEC was performed using an EnRich 650 column (Bio-Rad) on an Agilent LC‐1220 system in 20 mM Tris-HCl pH 8.0, 150 mM NaCl, 0.03% (wt/vol) DDM and 0.006% (wt/vol) CHS.

Samples used for nanodisc reconstitution and thermostabilization assays were purified as described for structural studies, except the protein was not subjected to TEV digestion.

#### Native mass spectrometry

Before MS analysis, detergent exchange into C_12_E_9_ (Anatrace) was carried out at 4 °C using a Superdex Increase 200 column on an Äkta Purifier FPLC system (GE Healthcare). Protein samples at a concentration of ~20 μM were introduced into the mass spectrometer using gold-coated borosilicate capillaries produced in house. Mass spectra were recorded on a hybrid Q-Exactive Orbitrap mass spectrometer (Thermo Fisher) modified for high *m*/*z* analysis. The settings were as follows: capillary voltage, 1.4 kV; HCD (Higher-energy C-trap dissociation) collision energy 50–200 V; HCD cell pressure 1 × 10^−9^ mbar; collision gas, argon. Data were visualized using Excalibur 4.0 software (Thermo Fisher).

#### Determination of lipid preferences of NHA2_ΔΝ_ and NHA2_ΔTM−1_ by GFP‐based thermal shift assay

The characterization of thermostability and the lipid thermal stabilization of NHA2_ΔΝ_ and NHA2_ΔTM−1_ were determined as previously described^36^. In brief, GFP fusions of NHA2_ΔΝ_ and NHA2_ΔTM−1_ were purified as described previously in buffer containing 0.03% (wt/vol) DDM and 0.006% (wt/vol) CHS. Samples were diluted on ice to a final concentration of ~0.05 mg ml^−1^ in buffer containing 20 mM Tris-HCl pH 8.0, 150 mM NaCl, 1% (wt/vol) DDM and 1% (wt/vol) β-d-octylglucoside (Anatrace). Individual sample aliquots of 100 μl were heated for 10 min over a temperature range of 20–80 °C in a PCR thermocycler (Veriti, Applied Biosystems) and heat‐denatured material was pelleted at 18,000*g* for 30 min at 4 °C. To avoid pellet contamination, only 80 μl of the respective supernatants was transferred to an optical 96-well plate (Greiner) and fluorescence was measured using a Fluoroskan plate reader (Thermo Fisher, using Skanlt software 6.0.2). The apparent *T*_M_ was calculated by plotting the mean GFP fluorescence intensity from three technical repeats per temperature and fitting it to a sigmoidal logistic regression using GraphPad Prism software. To screen for differential lipid stabilization, the GFP thermal shift (GFP‐TS) protocol was utilized with the modification that the protein was incubated for 30 min at 4 °C with the individual lipids at a final concentration of 3 mg ml^−1^ (1 mg ml^−1^ for PIP_2_) in 1% (wt/vol) DDM before the respective samples were heated at a single temperature (*T*_M_ of the respective construct + 5 °C). Stock solutions of the respective lipids 18:1 1,2-dioleoyl-*sn*-glycero-3-phosphate (sodium salt) (DOPA; Avanti, cat. no. 840875), 18:1 (Δ9-Cis) 1,2-dioleoyl-*sn*-glycero-3-phosphocholine (DOPC; Avanti, cat. no. 850375P), 18:1 (Δ9-Cis) 1,2-dioleoyl-*sn*-glycero-3-phosphoethanolamine (DOPE; Avanti, cat. no. 850725), 18:1 (Δ9-Cis) 1,2-dioleoyl-*sn*-glycero-3-phospho-(1′-rac-glycerol) (sodium salt) (DOPG; Avanti, cat. no. 840475), liver total lipid extract (bovine; Avanti, cat. no. 181104), yeast polar lipid extract (*S. cerevisiae*; Avanti, cat. no. 190001), brain extract from bovine brain type VII (Sigma-Aldrich, cat. no. B3635) and phosphatidylinositol bis-4,5-phosphate, 1,2-dioctanoyl (PIP_2_; Larodan, cat. no. 59-1124), 98% pure l-α-phosphatidylinositol from yeast (PI; Larodon, cat. no. 37-0132) were prepared by solubilization with water in 10% (wt/vol) DDM to a final concentration of 30 mg ml^−1^ (10 mg ml^−1^ for PIP_2_), overnight at 4 °C with mild agitation.

#### Preparation of bison NHA2_ΔΝ_ nanodisc reconstituted sample

MSP1E2 with an N-terminal hexa-His-tag and a TEV-protease recognition site was purified as previously described^59^ with the addition of His-tag removal by proteolytic cleavage using TEV protease during dialysis. After digestion and before storage, an additional reverse His-trap step was introduced to remove His-tagged TEV protease from the sample. 98% pure l-α-phosphatidylinositol from yeast (PI; Larodon, cat. no. 37-0132) was solubilized in chloroform (Merck) and dried using a rotary evaporator (Hei-Vap Core, Heidolph Instruments). Dry lipids were thoroughly resuspended in 20 mM Tris pH 7.5, 150 mM NaCl buffer at a final concentration of 20 mM. For the nanodisc reconstitution, SEC-purified bison NHA2_ΔΝ_-GFP fusion in buffer containing 0.03% DDM 0.006% CHS, MSP1E2 and the described PI lipid were mixed at a molar ratio of 1:5:50, respectively. The mixture was incubated on ice for 30 min. The solution was subsequently transferred to a tube containing ~30–50 mg of Bio-Beads (SM-2, Bio-Rad) previously equilibrated in 20 mM Tris-HCl pH 7.0, 150 mM NaCl buffer. The tube was incubated using a rotary shaker at 4 °C overnight. The Bio-Beads were sedimented by centrifugation (1 min, 3,000*g*, 4 °C) and the resulting supernatant was collected by pipetting. To remove the empty nanodiscs, the supernatant was incubated with Ni-NTA resin (~1 ml per mg of initial protein concentration used) pre-equilibrated with the same buffer in batches for 2–3 h on a rotary shaker. Non-bound supernatant was removed by washing the resin with five column volumes (CVs) of 20 mM Tris pH 7.5, 150 mM NaCl buffer and one CV of 20 mM Tris pH 7.5, 150 mM NaCl, 25 mM imidazole buffer. Bound nanodisc-incorporated protein was eluted in two CVs of 20 mM Tris pH 7.5, 150 mM NaCl, 250 mM imidazole buffer. The amount of protein was determined by GFP fluorescence on a plate reader. TEV protease was added 2:1 (wt/wt) and the eluate was dialyzed against 20 mM Tris pH 7.5, 150 mM NaCl buffer overnight. The mixture was then concentrated to an appropriate volume using a centrifugal concentrator (Amicon, Merck‐Millipore) with a molecular weight cutoff of 100 kDa and subjected to SEC using an EnRich 650 column (Bio-Rad) and an Agilent LC‐1220 system in 20 mM Tris-HCl pH 7.5, 150 mM NaCl. The peak fraction corresponding to the reconstituted bison NHA2 in nanodiscs (bands identified by SDS–PAGE) was concentrated to 2.7 mg ml^−1^ and used in cryo-EM experiments.

#### SSM-based electrophysiology of bison NHA2_ΔΝ_

SSM-based electrophysiology measurements were performed on a SURFE²R N1 device (Nanion Technologies) with liposome reconstituted protein^43^. For liposome reconstitution, yeast polar lipids (Avanti) solubilized in chloroform were completely dried using a rotary evaporator (Hei-Vap Core, Heidolph Instruments). Dry lipids were thoroughly resuspended in 20 mM Tris pH 8, 20 mM NaCl, 20 mM KCl, 5 mM MgCl_2_ buffer at a final concentration of 10 mg ml^−1^. The lipids were flash-frozen in liquid nitrogen and then thawed over eight freeze–thaw cycles. Unilamellar vesicles were prepared by extruding the resuspended lipids using an extruder (Avestin) with 400-nm polycarbonate filters (Whatman). The vesicles were destabilized by the addition of Na-cholate (0.65% final concentration). SEC-purified protein was added to the destabilized liposomes at a lipid-to-protein ratio (LPR) of 5:1 (or as otherwise stated) and incubated for 5 min at room temperature. Detergent was removed using a PD-10 desalting column, and the sample was collected in a final volume of 2.3 ml. Proteoliposomes were pelleted at 150,000*g* for 30 min and resuspended to a final lipid concentration of 1 mg ml^−1^ in 20 mM Tris pH 8.0, 20 mM NaCl, 20 mM KCl, 5 mM MgCl_2_ buffer and stored at −80 °C. Proteoliposomes were diluted 1:1 (vol/vol) with non-activating buffer (20 mM Tris at the desired pH, 300 mM KCl, 5 mM MgCl_2_) and sonicated using a bath sonicator before sensor preparation^43^.

Sensor preparation for SSM-based electrophysiology on the SURFE^2^R N1 system was performed as described previously^43^. During the experiments, NHA2 was activated by solution exchange from non-activating buffer to an activating buffer containing the substrate. Usually, *x* mM KCl was replaced by *x* mM NaCl or LiCl in the activating buffer. In some cases, 150 mM KCl was replaced by 150 mM choline chloride in the non-activating solution. In pH jump experiments, the activating buffer was titrated to the desired pH.

For measurements in the presence of pH gradients, a double solution exchange workflow was applied in which proteoliposomes were diluted and sonicated in non-activating buffer to equilibrate the intraliposomal pH (pH_i_) before sensor preparation. During the experiment, a non-activating solution with a different pH was applied to adjust the external pH (pH_o_). One second after establishment of the pH gradient, non-activating solution was exchanged by activating solution containing 150 mM NaCl to activate NHA2 in the presence of the pH gradient. After the experiment, the sensor was rinsed with non-activating solution at pH_i_ and incubated for 3 min to re-establish the intraliposomal pH.

### Cryogenic electron microscopy sample preparation and data acquisition

#### NHA2_ΔN_ structure in detergent

The purified bison NHA2_ΔN_ protein was individually applied to freshly glow-discharged Quantifoil R2/1 Cu300 mesh grids (Electron Microscopy Sciences). Grids were blotted for 3.0 s or 3.5 s under 100% humidity and plunge-frozen in liquid ethane using a Vitrobot Mark IV system (Thermo Fisher Scientific). Cryo-EM datasets were collected on a Titan Krios G2 electron microscope operated at 300 kV equipped with a GIF (Gatan) and a K2 summit direct electron detector (Gatan) in counting mode. The video stacks were collected at ×165,000, corresponding to a pixel size of 0.83 Å at a dose rate of 7.0–8.0 e^−^ per physical pixel per second using EPU2. The total exposure time for each movie was 10 s, leading to a total accumulated dose of 80 e^−^ Å^−2^, which was fractionated into 50 frames. All movies were recorded with a defocus range of −0.7 to −2.5 µm. The statistics of cryo-EM data acquisition are summarized in Table 1.

#### NHA2_ΔN_ structure in nanodiscs

The nanodisc-incorporated protein was individually applied to freshly glow-discharged Quantifoil R1.2/1.3 Cu300 mesh grids (Electron Microscopy Sciences). Grids were blotted for 4 s under 100% humidity and plunge-frozen in liquid ethane using a Vitrobot Mark IV instrument (Thermo Fisher Scientific). Cryo-EM datasets were collected on a Titan Krios G2 electron microscope operated at 300 kV equipped with a GIF (Gatan) and a K3 Summit direct electron detector (Gatan) in super-resolution counting mode. The video stacks were collected at ×130,000, corresponding to a pixel size of 0.68 Å at a dose rate of 14.3 e^−^ per physical pixel per second using EPU2. The total exposure time for each movie was 2 s, leading to a total accumulated dose of 63.5 e^−^ Å^−2^, which was fractionated into 40 frames. All movies were recorded with a defocus range of −0.6 to −2.2 µm. The statistics of cryo-EM data acquisition are summarized in Table 1.

### Image processing and model building

#### NHA2_ΔN_ structure in detergent

Dose-fractionated videos were corrected by MotionCorr2^60^, the contrast transfer function estimated by CTFFIND-4.1.13^61^, and dose-weighted images were used for auto-picking in RELION-3.0^62^ after 1,000 particles had been picked manually. The auto-picked particles were subjected to multiple rounds of 2D classification for selection and extracted for initial model generation in RELION^62^. The initial model was low-pass-filtered to 20 Å to serve as a starting reference for a further round of 3D auto-refinement in RELION-3.0 using all particles from suitable 3D classes. The 3D classes were iteratively refined to yield high-resolution maps in RELION with no symmetry applied, as imposing *C*2 symmetry gave poorer map quality. Per-particle CTF refinement and Bayesian polishing^63^ were next performed in RELION-3.0 and followed by non-uniform refinement and local refinement in cryoSPARC v2.14.2^33^ with a mask automatically generated by the default parameters in cryoSPARC. These maps were used to build an N-terminal extension helix, as this density was lost after signal subtraction (EMDB-13161 and PDB 7PII, Table 1). To improve the map features, signal subtraction of the micelle was carried out and converted from cryoSPARC to RELION format by UCSF pyem^64^. The subtracted particle data from the cryoSPARC set were refined in RELION-3.0 with SIDESPRITTER^65^. The overall resolution was estimated based on the gold-standard FSC cutoff at 0.143 and the local resolution was calculated from the two half-maps using RELION-3.0 (EMDB-13162 and PDB 7P1J, Table 1).

Homology modeling of NHA2 was performed using SWISS-MODEL with the crystal structure of NapA as a template (PDB 4BWZ). The NHA2 model was fitted as a rigid body into the map density using MOLREP in the CCP-EM suite^66^. After molecular replacement, manual model building was performed using COOT^67^, but not all side chains could be assigned. To guide model building, particles were subjected to one round of 3D classification for half of the assembly, without alignment, using a regularization parameter of 100, yielding five 3D classes. Four of the five 3D classes were then selected for focused refinement to a final resolution of 3.0 Å based on the gold-standard FSC (EMD-13597). The final NHA2 model (PDB 7P1J) underwent simulated annealing and NCS restraints using real-space refinement in PHENIX^68^. Finally, the NHA2_ΔN_ structure in detergent was compared to the structure in the nanodiscs (see next section) to confirm the side chain positioning of appropriate residues for regions showing poorer map quality. The refinement statistics for the final structure are summarized in Table 1.

#### NHA2_ΔN_ structure in nanodiscs

Dose-fractionated videos were corrected using Patch Motion and the dose-weighted micrographs were used for contrast transfer function estimation by Patch CTF. The dose-weighted images were used for auto-picking (Blob picker), classification and reconstruction. Initially auto-picked particles were used for one round of 2D classification to generate templates for a subsequent round of auto-picking. The auto-picked particles were subjected to multiple rounds of 2D classification, ab initio reconstruction and hetero refinement to remove ‘junk particles’. All the steps mentioned above were performed in cryoSPARC v3.2.0^33^. Additionally, particle removal was performed using 2D classification without alignment in RELION-3.1^62^. To convert the format from cryoSPARC to RELION, UCSF pyem^64^ was used. The particle stack was re-imported to cryoSPARC and heterologous refinement steps were performed. The final particle stack of the best class from heterologous refinement was further processed with non-uniform refinement and multiple local refinement steps in cryoSPARC v3.2.0. The last refinement step was performed in RELION-3.1 with SIDESPLITTER^65^. The overall resolution was estimated based on the gold-standard FSC cutoff at 0.143. The local resolution was calculated from the two half-maps using RELION-3.1.

#### Simulations

##### All-atom, explicit solvent MD simulations

NHA2 was simulated with all-atom, explicit solvent MD simulations in a realistic model of the lysosomal membrane. Simulations were based on the NHA2_ΔN_ nanodisc structure. The protein was embedded in a model for the lysosomal outer membrane, approximated by a composition of 65% PC (phosphatidylcholine), 20% PE (phosphatidylethanolamine), 6.5% PI, 4% SM (sphingomyelin), 4% PS and 0.5% PIP_2_ (refs. ^69,70^). No detailed data on the acyl chains composition of lipids in the lysosomal membrane were available. Therefore, experimental data for red blood cell plasma membranes^69^ were used as a starting point: PC 16:0/18:1, PE 16:0/18:1, SM 34:1, SM 40:1 and SM 40:2 (three SM species in equal proportions 1:1:1), PS 18:2/18:0, PI 18:0/20:4 and PIP_2_ with CHARMM36 lipid parameters^71^ POPC, POPE, SAPI, PSM/LSM/NSM, SLPS and SAPI25 with a total of 203 lipids in the cytosolic leaflet and 199 in the lysosolic leaflet. The protein/membrane system was simulated with a free NaCl concentration of 150 mM. The system was built with CHARMM-GUI v1.7^72–74^ using the CHARMM36 force field for proteins^75,76^ and lipids^71^, and the CHARMM TIP3P water model. Simulation systems contained around 179,000 atoms in a hexagonal unit cell with unit cell lengths *a* = 140 Å and *c* = 110 Å. All titratable residues were simulated in their default protonation states at pH 7.5, as predicted by PROPKA 3.1^77^, with the following exceptions. The p*K*_a_ of Lys459 was predicted as downshifted to ~7 due to the interaction with Arg431 and hence it was modeled as deprotonated (neutral). Additionally, the putative binding site residues Asp277 and Asp278 were simulated in all combinations of protonation states as described in the following. Equilibrium MD simulations were performed with GROMACS 2021.1^78^ on graphics processing units (GPUs). Initial relaxation and equilibration followed the CHARMM-GUI protocol^72^ with an initial energy minimization and six stages of equilibration with position restraints (with the harmonic force constant on protein and lipids). All simulations were run in the *NPT* ensemble at constant temperature (*T* = 303.15 K) and pressure (*P* = 1 bar). A stochastic velocity rescaling thermostat^79^ was used with a time constant of 1 ps, and three separate temperature-coupling groups for protein, lipids and solvent. Semi-isotropic pressure coupling was achieved with a Parrinello-Rahman barostat^80^ with a time constant of 5 ps and compressibility of 4.5 × 10^−5^ bar^−1^. The Verlet neighbor list was updated as determined by gmx mdrun for optimum performance during run time within a Verlet buffer tolerance of 0.005 kJ mol^−1^ ps^−1^. All simulations employed periodic boundary conditions and therefore Coulomb interactions were calculated with the fast-smooth particle-mesh Ewald (SPME) method^81^ with an initial real-space cutoff of 1.2 nm, and interactions beyond the cutoff were calculated in reciprocal space with a fast Fourier transform on a grid with 0.12-nm spacing and fourth-order spline interpolation. The balance between real-space and reciprocal-space calculations was optimized by the GROMACS GPU code at run time. The Lennard–Jones forces were switched smoothly to zero between 1.0 nm and 1.2 nm and the potential was shifted over the whole range and decreased to zero at the cutoff. Bonds to hydrogen atoms were converted to rigid holonomic constraints with the P-LINCS algorithm^82^ or SETTLE^83^ (for water molecules). The classical equations of motion were integrated with the leapfrog algorithm with a time step of 2 fs.

##### Simulations of different protonation states of ion-binding site ‘DD’ motif residues

MD simulations were performed with different protonation states of Asp277 and Asp278. We explicitly modeled all four combinations of protonation states for these two important residues. We hypothesized that the state in which NHA2 is likely to bind a sodium ion has both Asp277 and Asp278 deprotonated and is therefore negatively charged (state 0). As controls, we also modeled states less likely to support ion binding. In state 1, Asp277 is protonated (and neutral) while Asp278 remains deprotonated and negatively charged. In state 2, Asp277 remains deprotonated while Asp278 is protonated. Finally, the state least likely to support cation binding is state 3, with both aspartate residues protonated. Because NHA2 is a homodimer, we sampled two different charge states in a single simulation by preparing protomer A in state 0 (or 2) and protomer B in state 1 (or 3). Three independent repeats were performed by varying the initial velocities for simulations including state 0, whereas two repeats were carried out for the state 2/3 simulations (Supplementary Table 1).

##### Trajectory analysis

Analysis was carried out with Python scripts based on MDAnalysis^84^ (distances, root-mean-square deviation (RMSD), root-mean-square fluctuation (RMSF)). Sodium density maps were calculated with MDAnalysis from trajectories that were structurally superimposed on all Cα atoms of protomer A (for simulation f-01-0) or all protein Cα atoms (simulation f-23-1). Lipid density maps were calculated for all lipids, PI and POPE in simulation f-01-0. Time series of bound Na^+^ distances to carboxyl oxygen atoms in D277 and D278 were calculated for all Na^+^ ions as the shortest distance between Na^+^ and either Asp OD1, OD2 atoms. Binding of Na^+^ ions was assessed with a simple distance criterion: any Na^+^ ion within 3 Å of any carboxyl oxygen atom of Asp277 was considered bound. Molecular images were prepared in VMD 1.9.4^85^ and UCSF Chimera^86^.

##### Elastic network modeling and nanodisc transition

Elastic network models (ENMs) represent proteins as a simple network of residues (C-alphas) connected by elastic springs, so that diagonalization of the connectivity matrix renders 3N-6 eigenvectors or normal modes (NMs) that describe the large-scale conformation changes seen in experimental structures. To obtain an approximation to the intrinsic dynamics of NHA2, NMs were computed using the MD-refined potential ED-ENM as previously described^23,87,88^. Mid-frequency modes computed from the NHA2 outward conformation were found to display elevator-like movements between the dimer and transport domains capable of partially driving the transition towards inward-like states resembling the NapA inward conformation (Supplementary Fig. 7b). Transition pathways between NHA2 conformations in detergent and nanodiscs were generated with coarse-grained eBDIMS Langevin simulations^87,88^.

### Reporting Summary

Further information on research design is available in the Nature Research Reporting Summary linked to this Article.

## Online content

Any methods, additional references, Nature Research reporting summaries, source data, extended data, supplementary information, acknowledgements, peer review information; details of author contributions and competing interests; and statements of data and code availability are available at 10.1038/s41594-022-00738-2.

## Supplementary information

Supplementary Information Supplementary Table 1, Supplementary Figures 1-9 and Supplementary Source Data Fig. 9.

Reporting Summary

Peer Review Information

Supplementary Video 1 **Morph showing the structural transitions between the detergent and nanodisc** ***bison*** **NHA2**_**ΔN**_. **a**. NHA2 structural transitions as viewed from the extracellular side with core domain (pink), dimerization domain (green) and domain-swapped TM −1 (blue).

Supplementary Video 2 **b**. As in a., as viewed from the side.

Supplementary Video 3 **c**. As in a., as viewed from the cytoplasm.

Supplementary Video 4 **Intrinsic dynamics of TM −1 in** ***bison*** **NHA2**_**ΔN**_. **a**. Intrinsic dynamics of the NHA2 monomeric in nanodiscs with core domain (pink), dimerization domain (green) and domain-swapped TM −1 (blue) that spontaneously forms a conformation like that seen in detergent with TM −1 (grey).

Supplementary Video 5 **b**. Global NMA dynamics of the complete NHA2 dimer displays elevator-like mid-frequency modes towards the inward state.

Supplementary Data 1 Supplementary data for Supplementary Fig. 3

## Data Availability

The atomic coordinates and cryo-EM maps for NHA2 are available at the Protein Data Bank (PDB) and the Electron Microscopy Data Bank (EMDB) databases. The accession numbers are as follows: NHA2 detergent with extension helix, PDB 7P1I and EMD-13161; NHA2 detergent without extension helix, PDB 7P1J and EMD-13162; NHA2 detergent with focused classification, EMD-13597; NHA2 in nanodiscs, PDB 7P1K and EMD-13163. The datasets generated in the current study are available from the corresponding author on reasonable request. Source data are provided with this paper.

## Source data

Source Data Fig. 2 Statistical Source data
Source Data Fig. 3 Statistical Source data
Source Data Fig. 7 Statistical Source data
Source Data Extended Data Fig. 1 Statistical Source data
Source Data Extended Data Fig. 8 Statistical Source data
Source Data Extended Data Fig. 9 Statistical Source data
