# Author Correction: Structure, mechanism and lipid-mediated remodeling of the mammalian Na^+^/H^+^ exchanger NHA2

**DOI:** 10.1038/s41594-023-00970-4

**Published:** 2023-04-07

**Authors:** Rei Matsuoka, Roman Fudim, Sukkyeong Jung, Chenou Zhang, Andre Bazzone, Yurie Chatzikyriakidou, Carol V. Robinson, Norimichi Nomura, So Iwata, Michael Landreh, Laura Orellana, Oliver Beckstein, David Drew

**Affiliations:** 1 Department of Biochemistry and Biophysics, Stockholm University, Stockholm, Sweden; 2 Center for Biological Physics, Department of Physics, Arizona State University, Tempe, AZ USA; 3 Nanion Technologies GmbH, Munich, Germany; 4 Department of Chemistry, University of Oxford, Oxford, UK; 5 Graduate School of Medicine, Kyoto University, Konoe-cho, Yoshida, Sakyo-ku, Kyoto, Japan; 6 Department of Microbiology, Tumor and Cell Biology, Karolinska Institutet, Stockholm, Sweden; 7 Department of Oncology–Pathology, Karolinska Institutet, Stockholm, Sweden

**Keywords:** Membrane structure and assembly, Membrane lipids, Supramolecular assembly, Cryoelectron microscopy

Correction to: *Nature Structural & Molecular Biology* 10.1038/s41594-022-00738-2. Published online 16 February 2022.

In the version of this article initially published, there was a presentation error where the graph inset in Figure 7a was a duplication of Figure 7c; panel c has now been corrected in the HTML and PDF versions of the article.

