# Supplementary material for: Structure, mechanism and lipid-mediated remodeling of the mammalian Na+/H+ exchanger NHA2
Source: Nat Struct Mol Biol. 2022 Feb 16;29(2):108–20. doi: 10.1038/s41594-022-00738-2 (PMC8850199; doi:10.1038/s41594-022-00738-2)
Supplement: Supplementary file 1 — Supplementary Table 1, Supplementary Figures 1-9 and Supplementary Source Data Fig. 9. [file 41594_2022_738_MOESM1_ESM.pdf]

nature portfolio

https://doi.org/10.1038/s41594-022-00738-2

Supplementary information

Structure, mechanism and lipid-mediated remodeling of the mammalian Na^+/H^+ exchanger NHA2

In the format provided by the
authors and unedited

Supplementary Data Table 1: MD simulations.

simulation name	state-A	state-B	atoms	run length (ns)	binding-A	binding-B
f-01-0	0	1	178522	716.6	strong(1)	—
f-01-1	0	1	178522	390.1	—	—
f-01-2	0	1	178522	386.9	partial(2)	—
f-23-1	2	3	179068	374.1	—	—
f-23-2	2	3	179068	382.1	—	—

Protomers A and B were prepared with different protonation states: **state 0** (both Asp277 and Asp278 deprotonated), **state 1** (Asp277 protonated, Asp278 deprotonated), **state 2** (Asp277 deprotonated, Asp278 protonated), and **state 3** (both Asp277 and Asp278 protonated).

(1) Spontaneous binding from 349.9 ns onwards until the end of the simulation.
(2) Spontaneous partial binding from 327.0 ns onwards until the end of the simulation.

Supplementary Fig. 1

a.

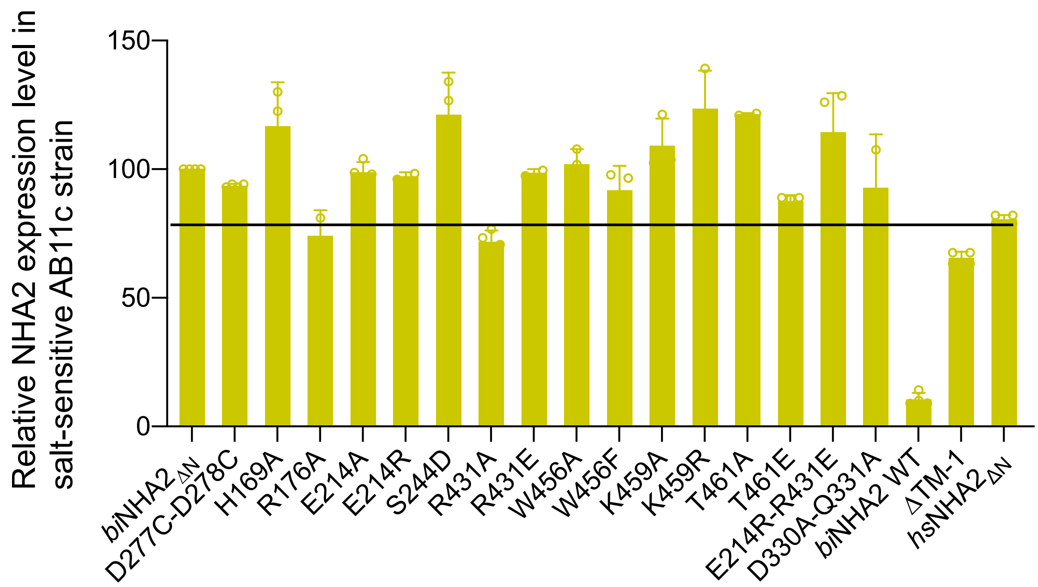

b.

FSEC traces of NHA2 constructs expressed in the salt-sensitive AB11c strain

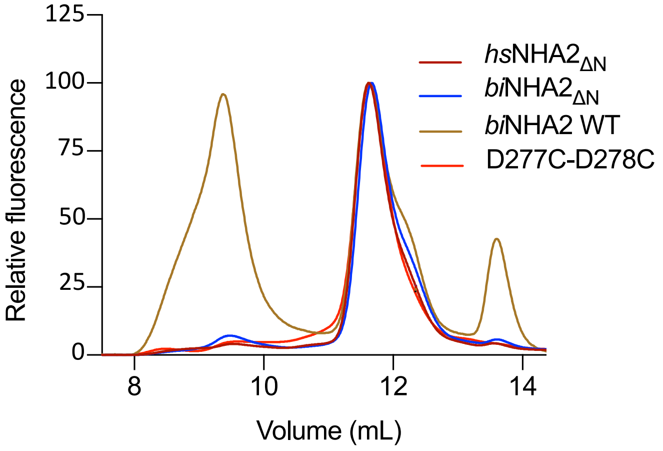

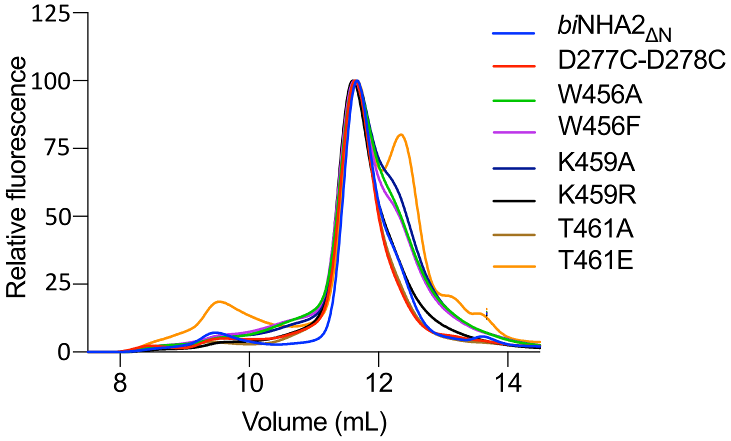

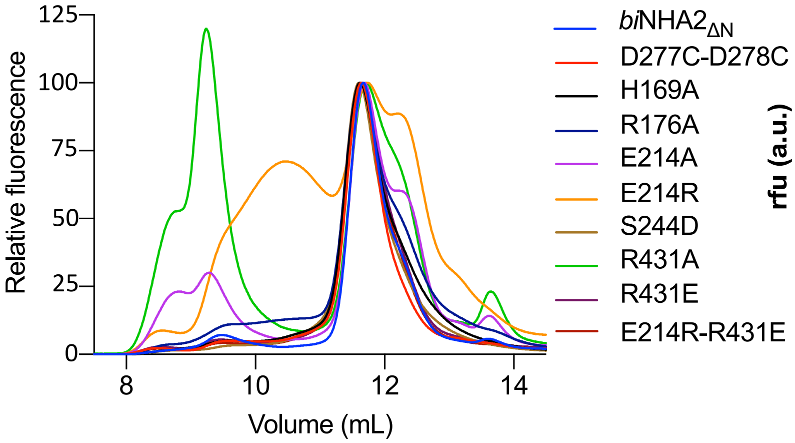

c.

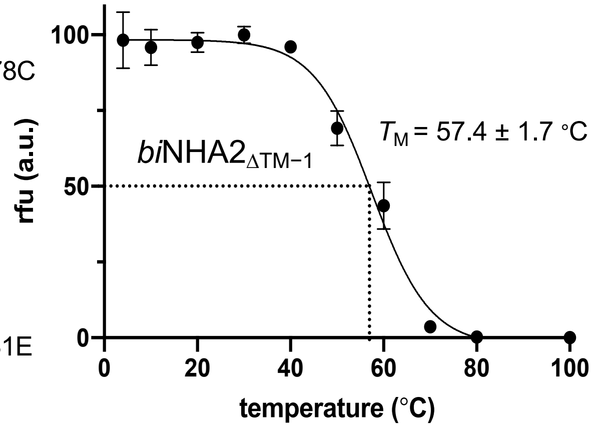

Supplementary Data Fig. 1. Expression and quality of NHA2-GFP fusions assessed by fluorescence-detection size exclusion chromatography (FSEC) in the ABc11 yeast strain. a. Bar represent normalized expression level compared to *bison* NHA2 $_{\Delta N}$ for various NHA2 constructs and mutants as measured by GFP fluorescence in yeast whole-cells. Errors bars represent the: mean values ± s.d. of n = 4 biologically independent samples for biNHA2$_{\Delta N}$, D277C-D278C, biNHA2 WT, ΔTM-1, E214A, R431A, T461E, E214R-R431E, hsNHA2$_{\Delta N}$; mean values ± s.d. of n = 3 biologically independent samples for H169A, S244D, W456A, W456F, K459A, K459R; range of n = 2 biologically independent samples for R176A, E214R, R431E, T461A, D330A-Q331A; note only the full-length bison NHA2 construct is poorly expressed (10-fold less than *bison* NHA2 $_{\Delta N}$) and has a large fraction of aggregates (see b.), yet still shows significant complementation at 10 mM LiCl and some complementation at 25 mM LiCl (Supplementary Fig. 3b). **b.** FSEC traces of listed NHA2 constructs extracted by DDM/CHS from ABc11 yeast membranes. The peak at 11.5 ml corresponds to the NHA2 homodimer and all the constructs shown here have a main FSEC peak at 11.5ml; the only constructs with a shifted predominant peak at 12.2 ml for the monomer are NHA2$_{TM\text{-}1}$ and NHA2 $_{\Delta N}$ (Q330A, D331A) shown in Fig. 3b. All constructs showing a minor monomer peak at 12.2 ml show poor folding as apparent by the aggregation peak in the void. e.g., E214R, R431A, T461E, and *bison* NHA2 full-length (WT). **c.** Thermal shift of purified monomeric NHA2 $_{TM\text{-}1}$-GFP in the presence of DDM/CHS. Data presented are normalized mean fluorescence as mean values ± s.e.m of n = 3 technical repeats; the apparent T_M was calculated with a sigmoidal 4-parameter logistic regression function.

Supplementary Fig. 2

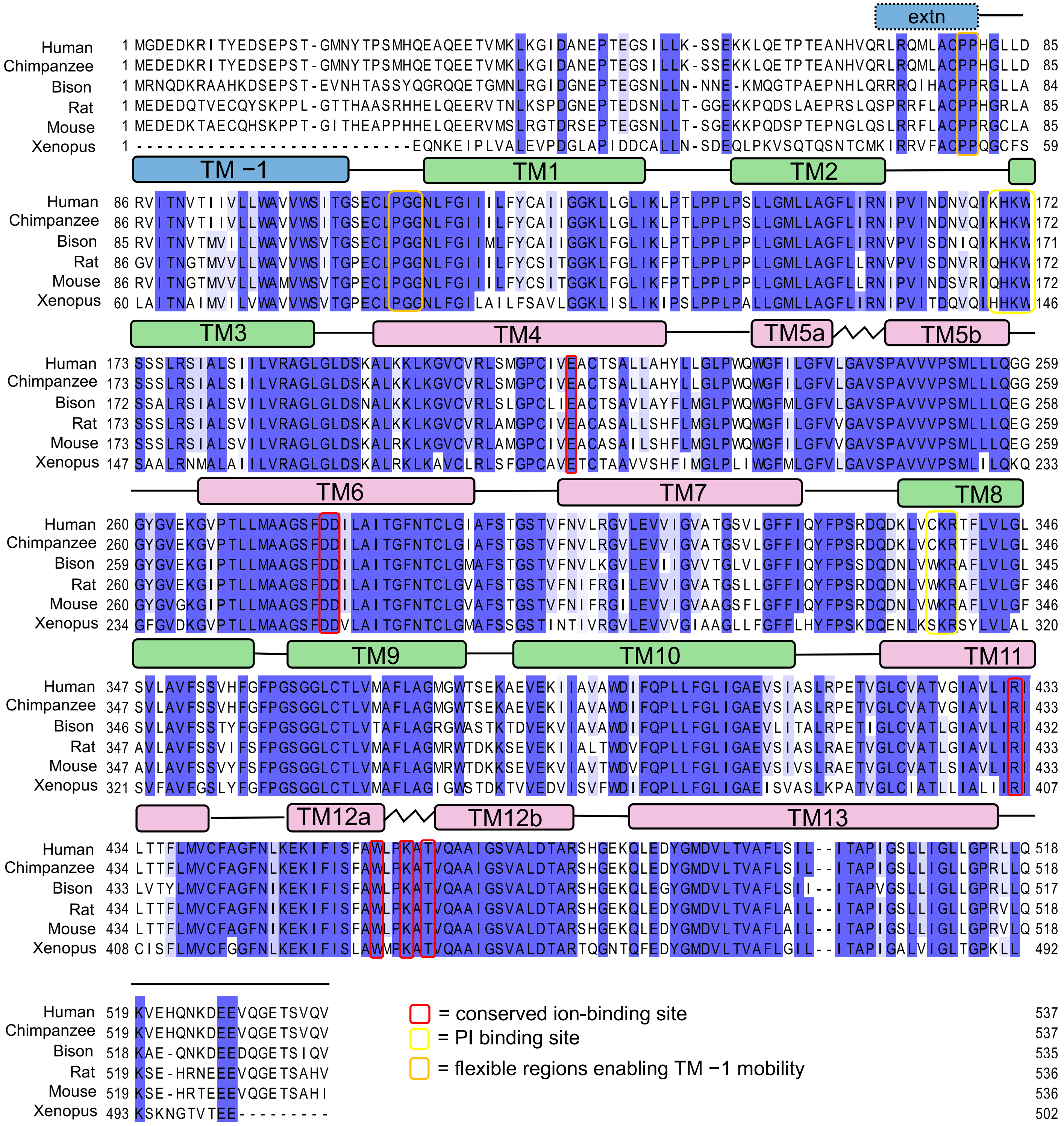

Supplementary Fig. 2. Multiple sequence alignment of *bison* NHA2 and eukaryotic homologues. NHA2 selected sequences of mammalian vertebrates, *Xenopus laevis* (African clawed frog) and *Human* (SLC9B2) were aligned. Residues with over 70% sequence identity are indicated by purple background. Conserved ion-binding site residues are highlighted with a red border. Positions which have been identified as part regions for lipid binding are highlighted with yellow border, and proline and glycine clusters associated with TM –1 mobility with orange border. Helix breakpoints (zigzag-line), and helices of the core 6-TM transport domain TMs (pink), dimerization domain (green) and domain-swapped helix TM –1 (blue) are shown and enumerated.

Supplementary Fig. 3

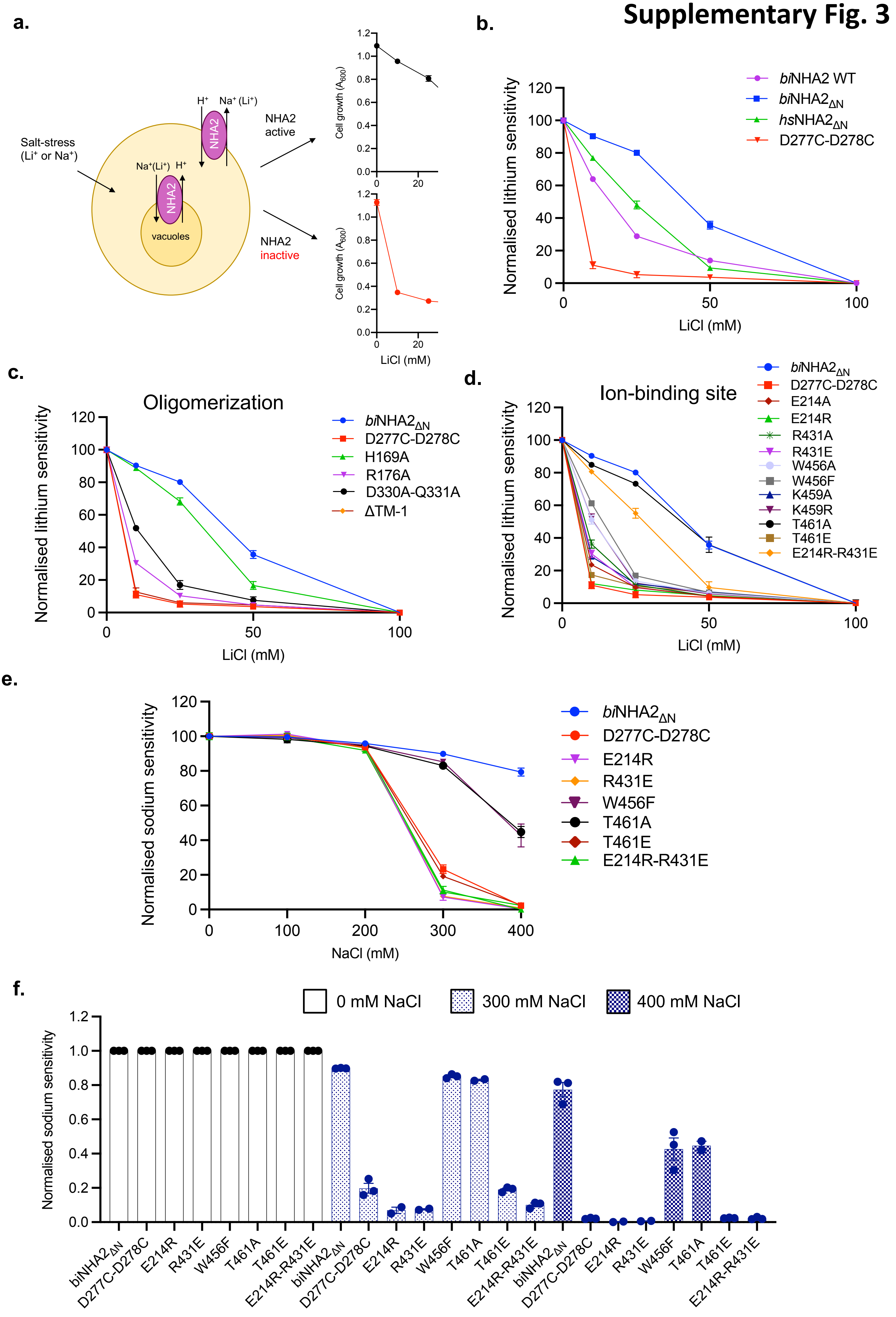

Supplementary Fig. 3. Functional complementation of Li^+ sensitivity in the yeast strain AB11c by heterologous expression of NHA2 constructs. **a**. Schematic showing the yeast salt-stress assay used for screening for NHA2 functional activity. **b.** Ab11c strain was transformed with *bison* NHA2 WT, *bison* NHA2 $_{\Delta N}$, *human* NHA2 $_{\Delta N}$ or *bison* NHA2 $_{\Delta N}$ with both Asp277 and Asp278 have been substituted with cysteine (D277C-D278C). Yeast were grown as outlined in Methods in -URA media supplemented with 2% galactose and LiCl at listed concentrations and growth was determined by optical density of the culture at 600 nm (OD_{600}) after 48 at 30°C and normalised against growth of *bison* NHA2 $_{\Delta N}$. Errors bars represent the mean values ± s.d. of n = 4 biologically independent samples **c**. As in a. for all *bison* NHA2 $_{\Delta N}$ structural and oligomerization mutants **d.** As in a, for *bison* NHA2 $_{\Delta N}$ ion-binding site mutants. Errors bars represent the mean values ± s.d. of n = 4 biologically independent samples. **e**. In most cases Li^+ was used to asses NHA2 complementation as Li^+ is more toxic to yeast cells and NHA2 $_{\Delta N}$ has a higher apparent affinity (K_D) for Li^+ vs. Na^+. Nevertheless, for those ion-binding site mutants that retained some Li^+-complementation, they were further assessed for Na^+ sensitivity. *above:* Yeast were grown as outlined in Methods in -URA media supplemented with 2% galactose and NaCl and growth was determined by optical density of the culture at 600 nm (OD_{600}) after 72 at 30°C and normalised against growth of *bison* NHA2 $_{\Delta N}$. *below:* To facilitate comparison the above data is shown as bar graphs for 300 and 400 mM NaCl. Errors bars represent the: mean values ± s.d. of n = 3 biologically independent samples for biNHA2$_{\Delta N}$, D277C-D278C, W456F, T461E and E214R-R431E; range of n = 2 biologically independent samples for E214R, R431E, T461A.

a. b.

c.

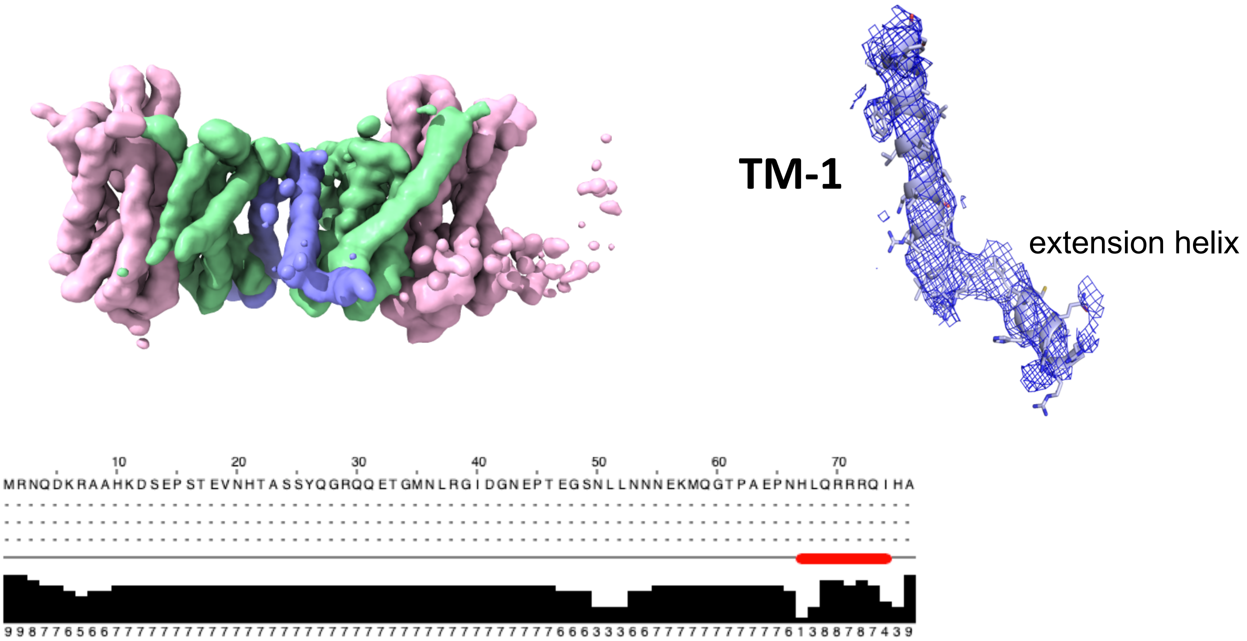

Supplementary Fig. 4. Cryo-EM density of *bison* NHA2 $_{\Delta N}$ in detergent before masked refinement a. Cryo-EM density map of NHA2 $_{\Delta N}$ prior to masked refinement with the 6-TM core transport domains (coloured in pink), the dimer domain (coloured in green) and the N-terminal domain detergent with additional density that -swapped helix TM –1 from the neighbouring protomer (blue). **b**. Cryo-EM density map for that fitted a short helix at the N-terminus. **c**. The full-length *bison* NHA2 N- sequence was subjected to secondary structure prediction by Jpred (https://www.compbio.dundee.ac.uk/jpred/) with the predicted N-terminal helix that was modelled shown as a red-bar.

Supplementary Fig. 5

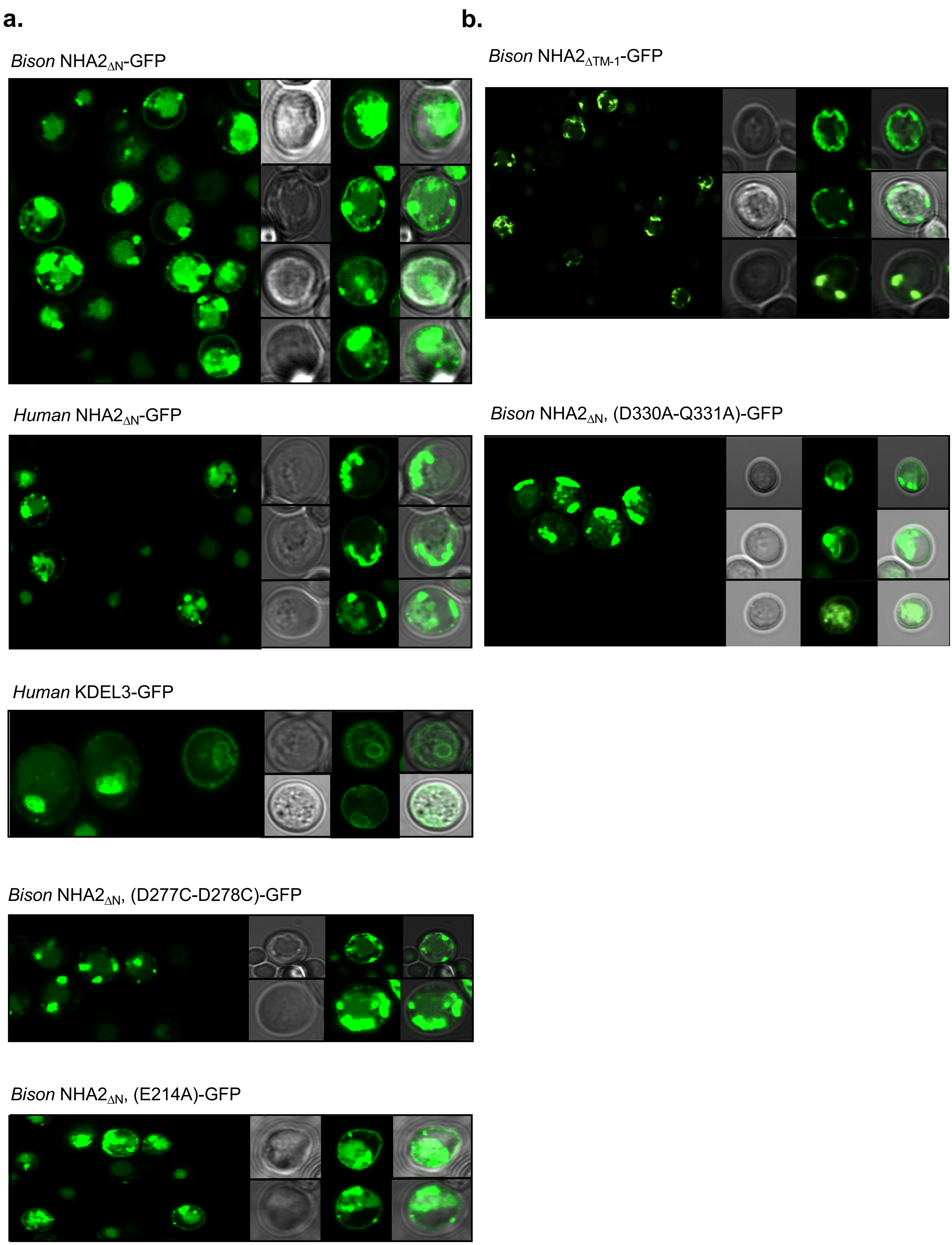

Supplementary Fig 5. Confocal localization of *human* and *bison* NHA2$_{\Delta N}$-GFP fusions and derived mutants. a. Confocal and widefield images of *Bison* NHA2$_{\Delta N}$-GFP, *Human* NHA2$_{\Delta N}$-GFP, *Human* KDEL3-GFP (ER-marker protein), and non-active ion-binding site mutants *Bison* NHA2$_{\Delta N}$ (D277C-D78C)-GFP, and *Bison* NHA2$_{\Delta N}$ (E214A)-GFP in the salt-sensitive strain Ab11c. Expression of NHA2 in either plasma membrane and/or vacuoles will rescue salt-stress similar to the vacuolar Na^+/H^+ exchanger NHX1 (J. Biol. Chem. 272, 26145-26152; Microbiology 145, 3221-3228; PNAS, 2007 104 (47) 18677-18681). **b.** As in a., for *Bison* NHA2$_{\Delta TM-1}$-GFP and the TM7-TM8 loop mutant *Bison* NHA2$_{\Delta N}$ (D330A-Q331A)-GFP that disrupt homodimerization and yet show similar localization to *Bison* and *Human* NHA2$_{\Delta N}$-GFP and clearly different from the ER marker (KDEL3).

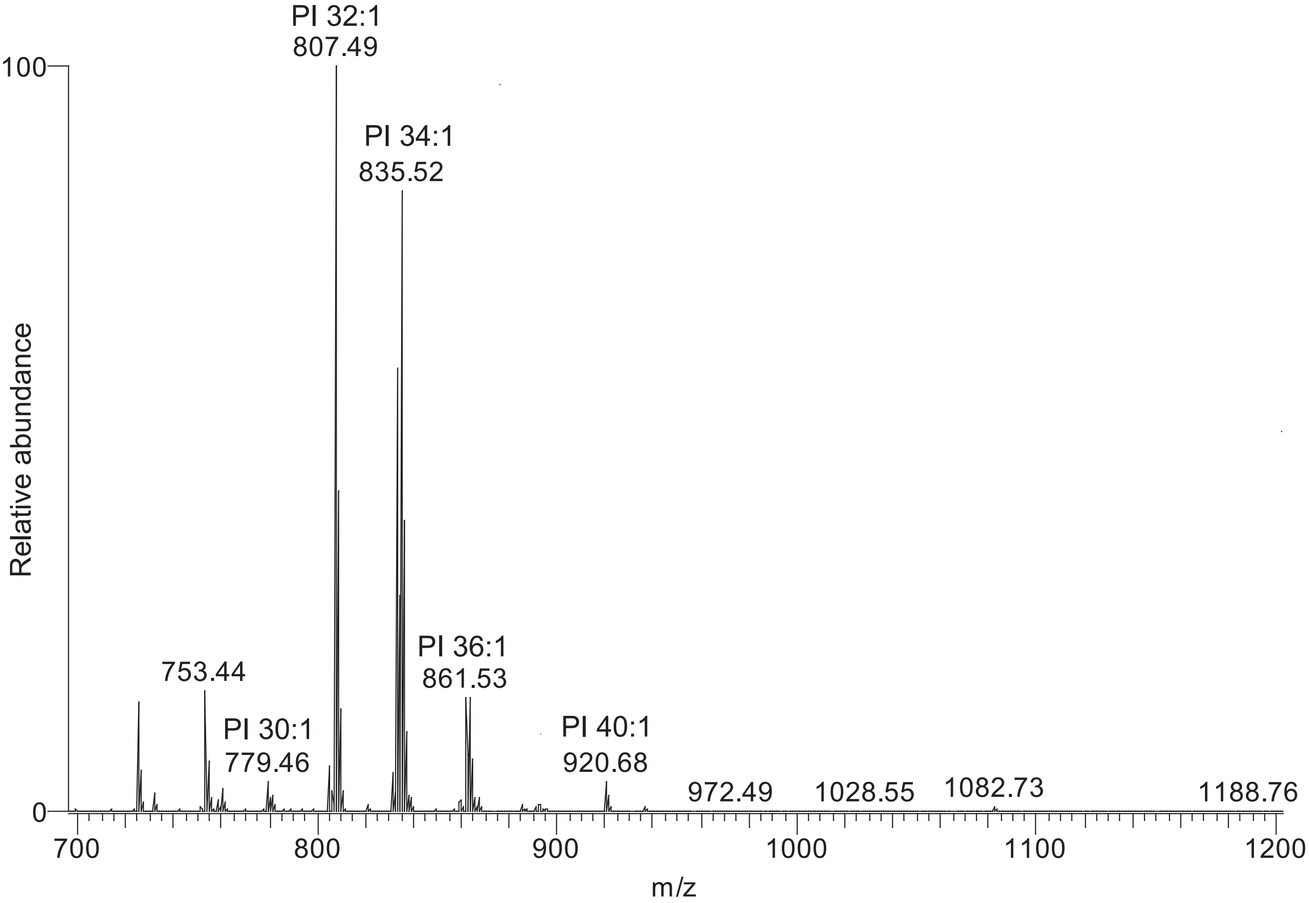

Supplementary Fig 6. Native MS of pure yeast PI lipids. Mass spectra of commercially available PI lipids from yeast reported to be >98% pure (product no: 37-0132, Laradon). Mass spectrum were recorded in negative ionization mode and because of negative mode, the mass label in the PI spectrum is the mass minus one proton. The two most abundant PI lipids (32:1 and 34:1) in this mixture are consistent with the reportedly most abundant PI lipids from lipidomics of *S. cerevisiae*, which in yeast are as abundant as PC lipids (PLoS ONE 8(9): e73936; Genome Res. 2003 Feb;13(2):244-53.).

Supplementary Fig. 7

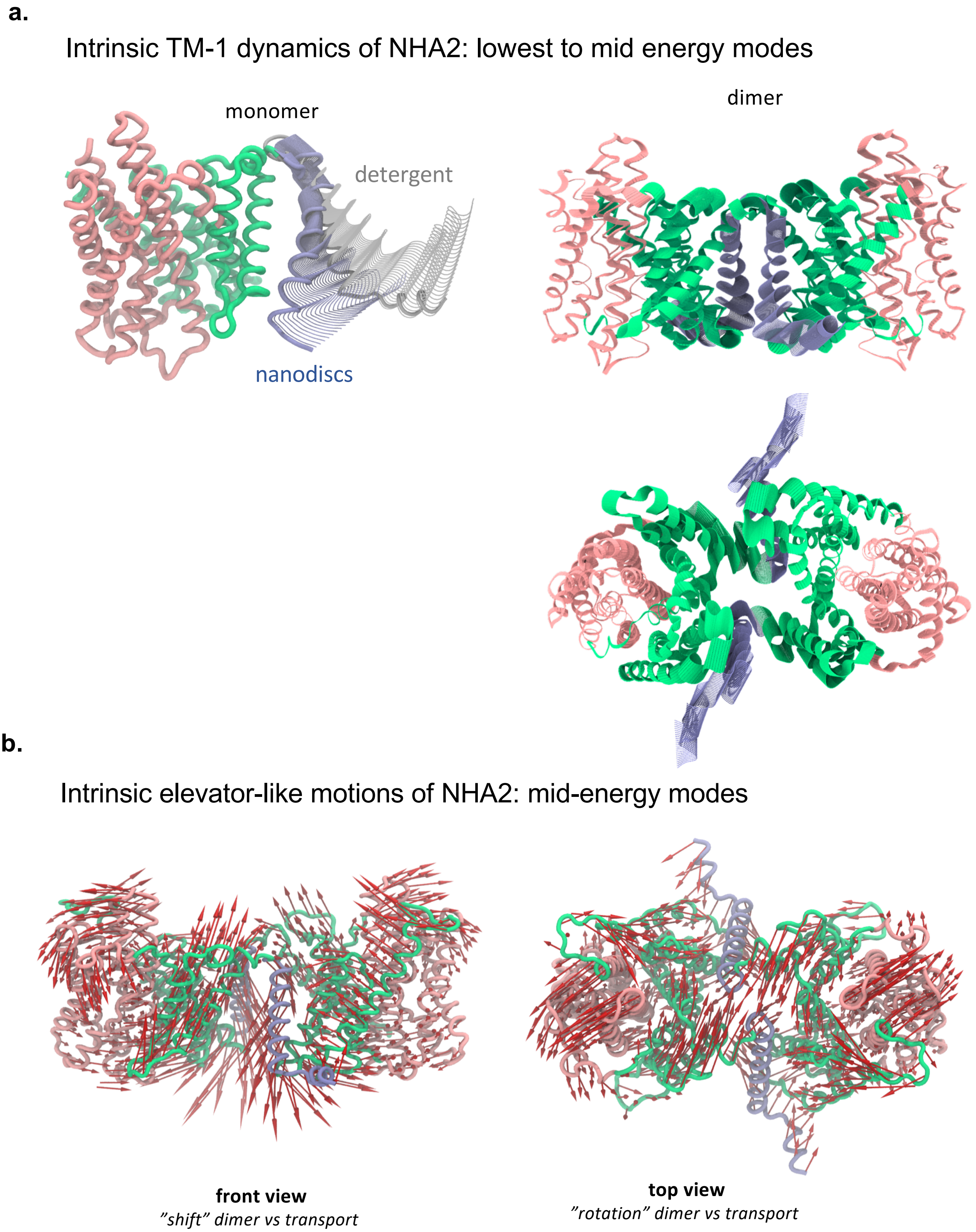

Supplementary Fig. 7. Elastic Network Modelling of NHA2 dimers and monomers. a. Intrinsic TM −1 dynamics of both the monomeric (left) and the dimeric (right) forms of NHA2 $_{\Delta N}$. The lowest frequency modes of bison NHA2 $_{\Delta N}$ are dominated by intrinsic TM−1 dynamics that spontaneously interconvert between the two observed structural conformations (detergent and nanodisc). **b**. Elevator-like motions of both monomeric and dimeric forms of NHA2. Mid-range modes (NM12-18) encode displacements of the dimer versus the transport domain that can drive transitions to inward-like states resembling the inward conformation of NapA.

Supplementary Fig. 8

a.

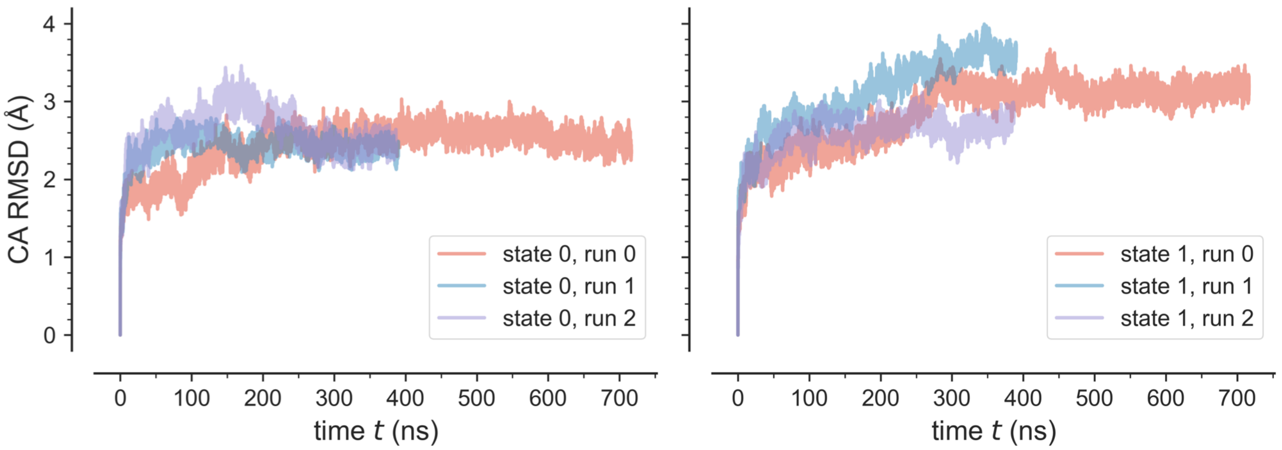

b.

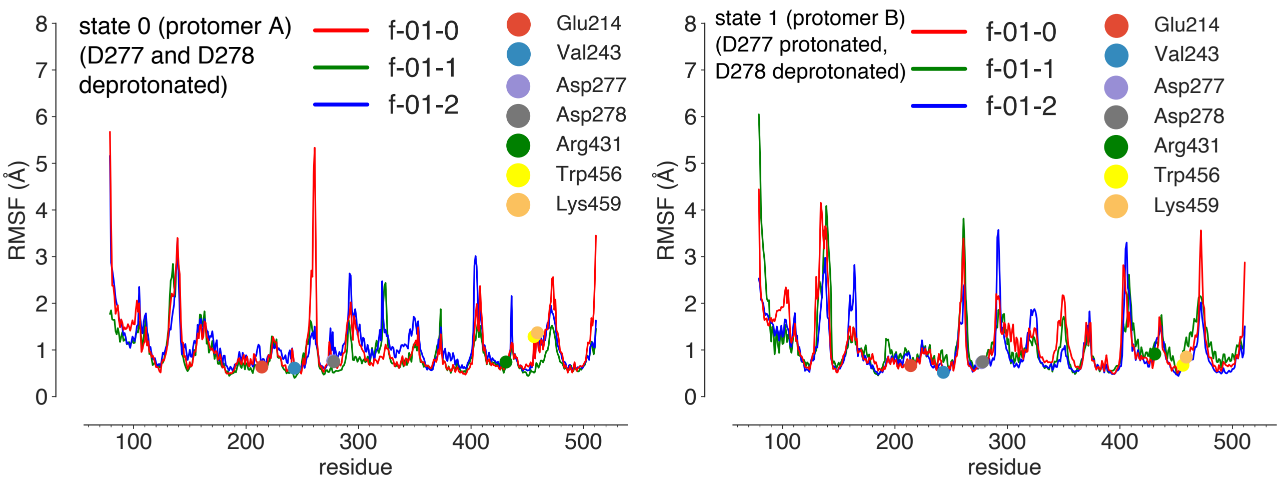

c.

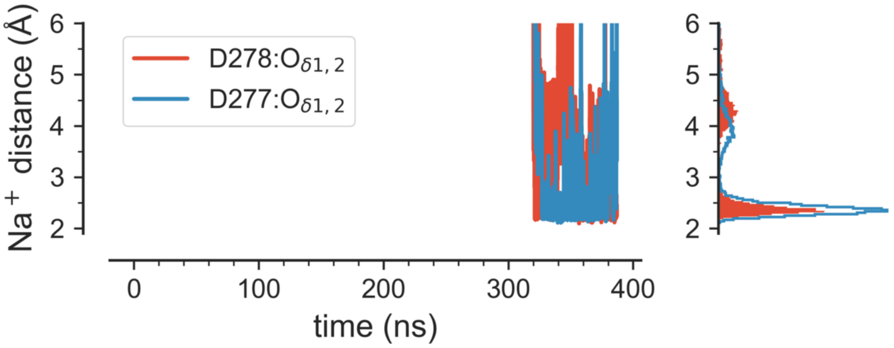

d.

residue	n1
Gly274:O	0.361
Ser275:O	0.035
Asp277-OD*	1.343
Asp278-OD*	0.956
Gly360:O	0.001
water	3.491
**Total**	**6.187**

e.

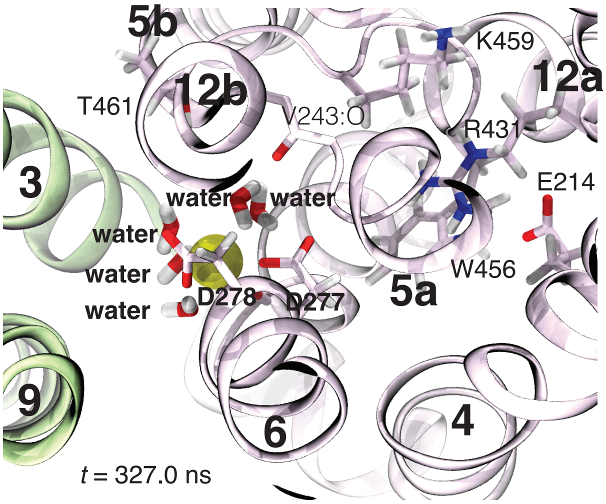

f.

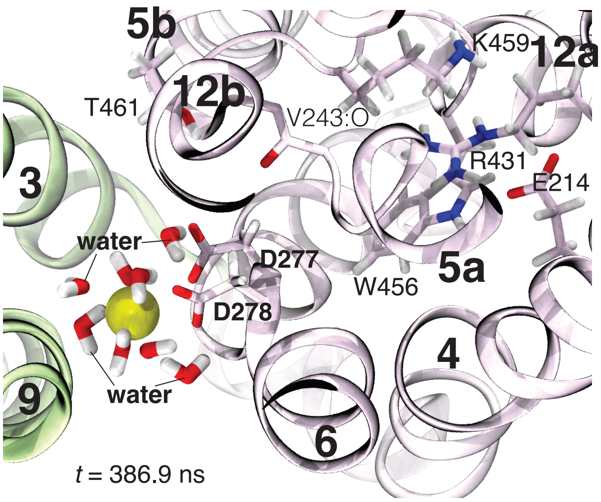

Supplementary Fig. 8 Average structural variability of the NHA2 nanodisc structure in MD simulations and ion-binding events. Conformational drift from the initial experimental structure and local flexibility was assessed with root mean square quantities. Protomers A and B, which were simulated in different protonation states were structurally superimposed on themselves and analyzed independently. *Left:* state 0 (D277 and D278 deprotonated). *right:* state 1 (D277 protonated, D278 deprotonated). **a.** C_α root mean square distances (RMSD) from the first trajectory frame after energy minimization and short equilibration MD. **b.** C_α root mean square fluctuations (RMSF) Locations of key residues are highlighted as coloured circles. **c**. In simulation f-01-2, a sodium ion partially bound to the ion binding residues in protomer A (protonation state 0, i.e., D277 and D278 negatively charged). Shortest distance of any sodium ion to either carboxylate oxygen in D277 or D278: timeseries (left) and histogram (right). **d**. Coordination of the partially bound sodium ion, quantified by the average contributions of oxygen atoms from different residues to the first hydration shell of the bound sodium ion, n_1. $n_1<0.001$ are not shown. Only backbone carbonyl oxygen atoms ("O") or the delta oxygen atoms of the carboxylate groups participated in binding. **e**. Top view (from the lysosolic side), with dimer domain in light green and core domain in light purple at time 327.0 ns. Water molecules within 3 Å of the sodium ion (yellow) are included. **f**. Top view at the end of the simulation at 386.9 ns.

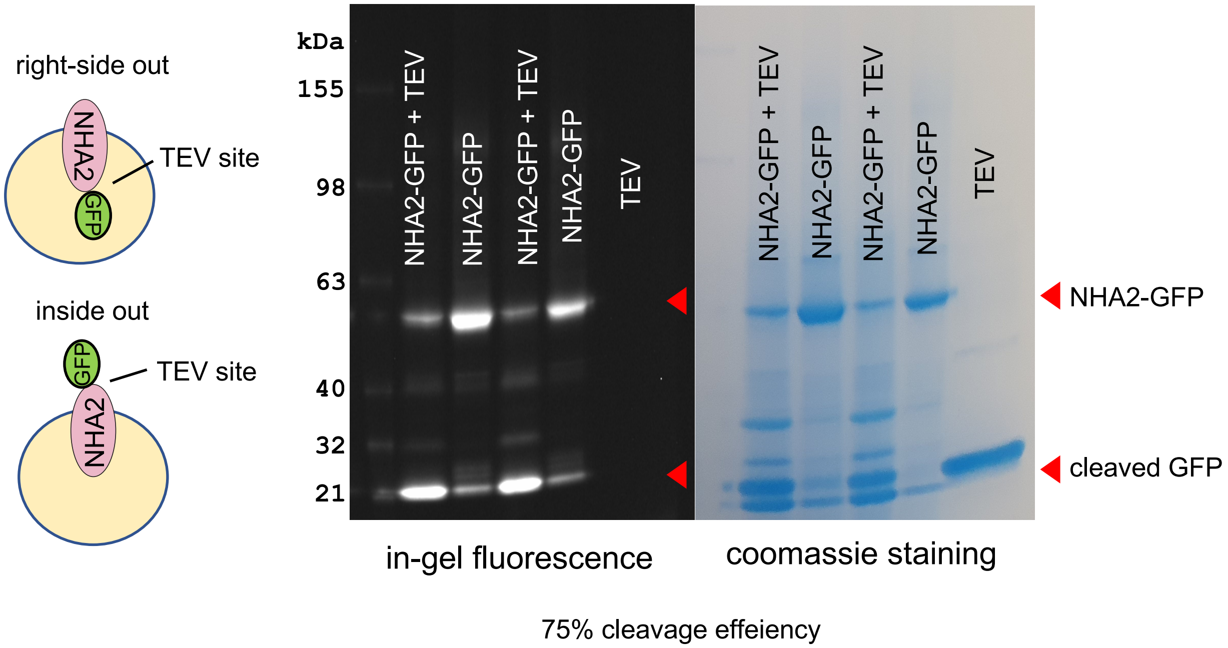

Supplementary Fig. 9 Estimation of *bison* NHA2$_{\Delta N}$ orientation into liposomes. *Left:* Cartoon showing the possible orientations of NHA2$_{\Delta N}$ -GFP upon insertion into liposomes with the cleavage site inaccessible (right-side out) or accessible (inside-out). *Right:* NHA2-GFP proteoliposomes were incubated o/n with a 1:1 ratio of TEV protease or with an equal volume of buffer. TEV Cleavage efficiency was estimated at an average of 75.3 ± 1.6 (%) by band intensities of SDS-page by in-gel fluorescence (left) and Coomassie staining (right). n = 2 biologically independent experiments.

Source Data Supplementary Fig. 9

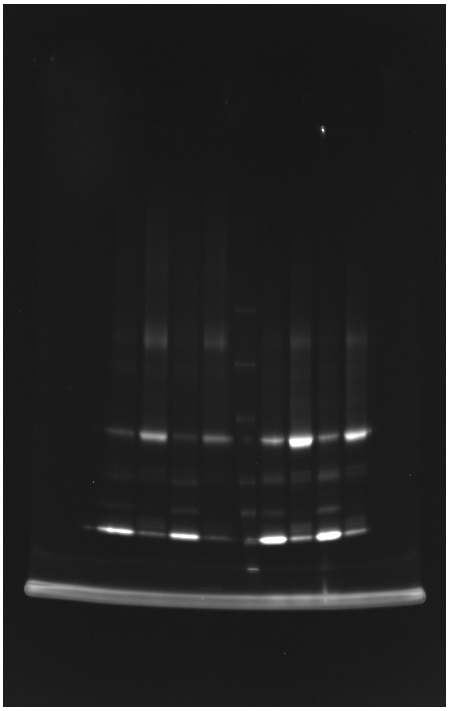

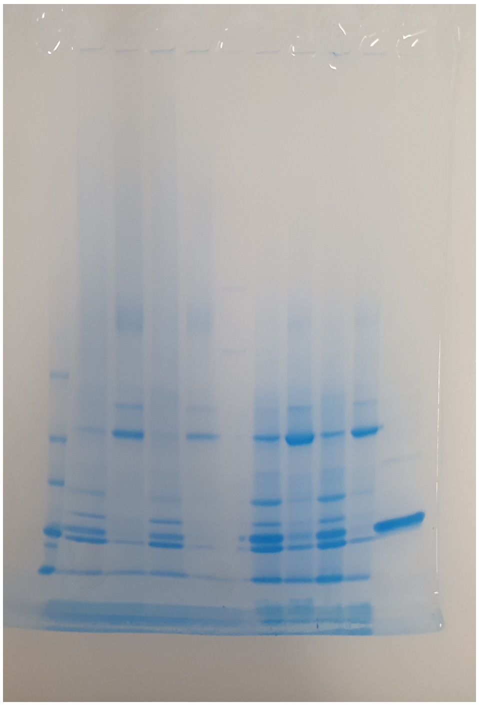

Uncropped gel for Supplementary Fig. 9
