# Supplementary material for: Structure, mechanism and lipid-mediated remodeling of the mammalian Na+/H+ exchanger NHA2
Source: Nat Struct Mol Biol. 2022 Feb 16;29(2):108–20. doi: 10.1038/s41594-022-00738-2 (PMC8850199; doi:10.1038/s41594-022-00738-2)
Supplement: Supplementary file 2 — Reporting Summary [file 41594_2022_738_MOESM2_ESM.pdf]

nature research

Corresponding author(s): David Drew

Reporting Summary

Nature Research wishes to improve the reproducibility of the work that we publish. This form provides structure for consistency and transparency in reporting. For further information on Nature Research policies, see Authors & Referees and the Editorial Policy Checklist.

Statistical parameters

When statistical analyses are reported, confirm that the following items are present in the relevant location (e.g. figure legend, table legend, main text, or Methods section).

n/a	Confirmed	
☐	☒	The exact sample size (*n*) for each experimental group/condition, given as a discrete number and unit of measurement
☐	☒	An indication of whether measurements were taken from distinct samples or whether the same sample was measured repeatedly
☒	☐	The statistical test(s) used AND whether they are one- or two-sided<br>*Only common tests should be described solely by name; describe more complex techniques in the Methods section.*
☒	☐	A description of all covariates tested
☒	☐	A description of any assumptions or corrections, such as tests of normality and adjustment for multiple comparisons
☐	☒	A full description of the statistics including central tendency (e.g. means) or other basic estimates (e.g. regression coefficient) AND variation (e.g. standard deviation) or associated estimates of uncertainty (e.g. confidence intervals)
☒	☐	For null hypothesis testing, the test statistic (e.g. *F*, *t*, *r*) with confidence intervals, effect sizes, degrees of freedom and *P* value noted<br>*Give P values as exact values whenever suitable.*
☒	☐	For Bayesian analysis, information on the choice of priors and Markov chain Monte Carlo settings
☒	☐	For hierarchical and complex designs, identification of the appropriate level for tests and full reporting of outcomes
☒	☐	Estimates of effect sizes (e.g. Cohen's *d*, Pearson's *r*), indicating how they were calculated
☐	☒	Clearly defined error bars<br>*State explicitly what error bars represent (e.g. SD, SE, CI)*

Our web collection on statistics for biologists may be useful.

Software and code

Policy information about availability of computer code

Data collection	SIDESPLITTER
Data analysis	Prism 7 - for data plotting and analysis<br>EPU2<br>Phenix 1.11.1-2575 or 1.18rc5_3822 - Structural refinement software suite<br>PyMol - Molecular graphics software<br>Coot - Structural model building<br>Gromacs 2021.1 - MD simulation package<br>MODELLER version 9.21 - MD simulation package<br>Coot - Structural model building<br>Relion 3.0 or 3.1<br>cryoSPARC v2.14.2 or 3.2.0<br>MOLREP in CCP-EM suite<br>CTFFIND4 4.1.13<br>Chimera 1.13.1<br>MotionCor2 1.1.0<br>UCSF-pyem<br>CHARMM36<br>CHARMM-GUI v1.7

MDAnalysis

For manuscripts utilizing custom algorithms or software that are central to the research but not yet described in published literature, software must be made available to editors/reviewers upon request. We strongly encourage code deposition in a community repository (e.g. GitHub). See the Nature Research guidelines for submitting code & software for further information.

Data

Policy information about availability of data

All manuscripts must include a data availability statement. This statement should provide the following information, where applicable:

- Accession codes, unique identifiers, or web links for publicly available datasets
- A list of figures that have associated raw data
- A description of any restrictions on data availability

The 3D cryo-EM density maps have been deposited in Electron Microscopy data bank with association numbers EMD-13161, EMD-13162 and EMD-13163. The protein coordinates have been deposited to Protein Data Bank with association codes 7P1I, 7P1J and 7P1K.

Field-specific reporting

Please select the best fit for your research. If you are not sure, read the appropriate sections before making your selection.

☒ Life sciences ☐ Behavioural & social sciences ☐ Ecological, evolutionary & environmental sciences

For a reference copy of the document with all sections, see nature.com/authors/policies/ReportingSummary-flat.pdf

Life sciences study design

All studies must disclose on these points even when the disclosure is negative.

Sample size	Biochemical assays and were typically performed at least in triplicate (n =3) to ascertain accurate values for data shown. Statistical methods were not used to determine sample size, but were used to calculate standard deviations or standard error.
Data exclusions	No data was excluded.
Replication	All biochemical assays were repeated at least 3 times and the results were reproduced each time.
Randomization	Samples were not randomized for biochemical assays as this is not applicable.
Blinding	No blinding was carried out for biochemical and structural analysis as this is not applicable.

Reporting for specific materials, systems and methods

Materials & experimental systems

n/a	Involved in the study
☐	☒ Unique biological materials
☒	☐ Antibodies
☒	☐ Eukaryotic cell lines
☒	☐ Palaeontology
☒	☐ Animals and other organisms
☒	☐ Human research participants

Methods

n/a	Involved in the study
☒	☐ ChIP-seq
☒	☐ Flow cytometry
☒	☐ MRI-based neuroimaging

Unique biological materials

Policy information about availability of materials

Obtaining unique materials	No restrictions apply for material used in this study, such as plasmids and strains.
