# Supplementary material for: Structure, mechanism and lipid-mediated remodeling of the mammalian Na+/H+ exchanger NHA2
Source: Nat Struct Mol Biol. 2022 Feb 16;29(2):108–20. doi: 10.1038/s41594-022-00738-2 (PMC8850199; doi:10.1038/s41594-022-00738-2)
Supplement: Supplementary file 3 — Peer Review Information [file 41594_2022_738_MOESM3_ESM.pdf]

Peer Review Information

Journal: Structural and Molecular Biology
Manuscript Title: Structure, mechanism and lipid-mediated remodelling of the mammalian Na+/H+ exchanger NHA2
Corresponding author name(s): Professor David Drew, Dr Roman Fudim, Dr Oliver Beckstein

Editorial Notes:

Parts of this Peer Review File have been redacted as indicated to remove third-party material.

Reviewer Comments & Decisions:

Decision Letter, initial version:

18th Aug 2021

Dear David,

Thank you again for submitting your manuscript "Structure and lipid-mediated remodelling mechanism of the Na+/H+ exchanger NHA2". I apologize for the delay in responding, which resulted from the difficulty in obtaining suitable referee reports. Nevertheless, we now have comments (below) from the 3 reviewers who evaluated your paper. In light of those reports, we remain interested in your study and would like to see your response to the comments of the referees, in the form of a revised manuscript.

You will see that both reviewers 1 (an expert on transporter structure-function) and 2 (an expert on transporters, cryo-EM, and MD simulations) are very positive about the interest and quality of the work, but do ask for several revisions. Reviewer 1 requests that data on subcellular localization of NHA2 mutants expressed in yeast is added. Reviewer 2 has suggestions for streamlining the manuscript to make it more accessible to a general audience, and for further supporting or clarifying some of the claims. This reviewer also has some questions regarding cryo-EM data processing. Reviewer 3 (an expert on transporters and cryo-EM) is more hesitant and points out potentially serious issues with the structural map for the detergent structure. I would like to stress that we consider addressing this point to the satisfaction of the reviewer essential for further consideration of

the study here at NSMB. The referee also requests additional controls and clarifications for several experiments. Finally, all reviewers have useful suggestions to improve the presentation and discussion of the findings.

We are committed to providing a fair and constructive peer-review process. Do not hesitate to contact us if there are specific requests from the reviewers that you believe are technically impossible or unlikely to yield a meaningful outcome. Please be sure to address/respond to all concerns of the referees in full in a point-by-point response and highlight all changes in the revised manuscript text file.

We appreciate the requested revisions are extensive. We thus expect to see your revised manuscript within 6 months. If you cannot send it within this time, please let us know. We will be happy to consider your revision as long as nothing similar has been accepted for publication at NSMB or published elsewhere. Should your manuscript be substantially delayed without notifying us in advance and your article is eventually published, the received date would be that of the revised, not the original, version.

As you already know, we put great emphasis on ensuring that the methods and statistics reported in our papers are correct and accurate. As such, if there are any changes that should be reported, please submit an updated version of the Reporting Summary along with your revision.

Reporting Summary:
https://www.nature.com/documents/nr-reporting-summary.pdf

Please note that the form is a dynamic 'smart pdf' and must therefore be downloaded and completed in Adobe Reader.

When submitting the revised version of your manuscript, please pay close attention to our href="https://www.nature.com/nature-research/editorial-policies/image-integrity">Digital Image Integrity Guidelines.

Finally, please ensure that you retain unprocessed data and metadata files after publication, ideally archiving data in perpetuity, as these may be requested during the peer review and production process or after publication if any issues arise.

If there are additional or modified structures presented in the final revision, please submit the corresponding PDB validation reports.

SOURCE DATA: we urge authors to provide, in tabular form, the data underlying the graphical representations used in figures. This is to further increase transparency in data reporting, as detailed in this editorial (http://www.nature.com/nsmb/journal/v22/n10/full/nsmb.3110.html). Spreadsheets can be submitted in excel format. Only one (1) file per figure is permitted; thus, for multi-paneled figures, the source data for each panel should be clearly labeled in the Excel file; alternately the data can be provided as multiple, clearly labeled sheets in an Excel file. When submitting files, the title field should indicate which figure the source data pertains to. We encourage our authors to provide source data at the revision stage, so that they are part of the peer-review process.

While we encourage the use of color in preparing figures, please note that this will incur a charge to

partially defray the cost of printing. Information about color charges can be found at http://www.nature.com/nsmb/authors/submit/index.html#costs

We require deposition of coordinates (and, in the case of crystal structures, structure factors) into the Protein Data Bank with the designation of immediate release upon publication (HPUB). Electron microscopy-derived density maps and coordinate data must be deposited in EMDB and released upon publication. To avoid delays in publication, dataset accession numbers must be supplied with the final accepted manuscript and appropriate release dates must be indicated at the galley proof stage. Please find the complete NRG policies on data availability at http://www.nature.com/authors/policies/availability.html.

Nature Structural & Molecular Biology is committed to improving transparency in authorship. As part of our efforts in this direction, we are now requesting that all authors identified as ‘corresponding author’ on published papers create and link their Open Researcher and Contributor Identifier (ORCID) with their account on the Manuscript Tracking System (MTS), prior to acceptance. This applies to primary research papers only. ORCID helps the scientific community achieve unambiguous attribution of all scholarly contributions. You can create and link your ORCID from the home page of the MTS by clicking on ‘Modify my Springer Nature account’. For more information please visit please visit www.springernature.com/orcid.

Please use the link below to submit your revised manuscript and related files:

[REDACTED]

Note: This URL links to your confidential home page and associated information about manuscripts you may have submitted, or that you are reviewing for us. If you wish to forward this email to co-authors, please delete the link to your homepage.

We look forward to seeing the revised manuscript and thank you for the opportunity to review your work.

Kind regards,
Florian

Florian Ullrich, Ph.D.
Associate Editor
Nature Structural & Molecular Biology
ORCID 0000-0002-1153-2040

Reviewers' Comments:

Reviewer #1:
Remarks to the Author:

Summary: This original structure of a unique member of the mammalian superfamily of Na+/H+ exchangers, SLC9B2/NHA2, makes multiple new and significant contributions. The quality and quantity of data, and its presentation are exceptional. The literature has been nicely integrated into this study

and appropriately referenced for maximal impact. My main caveat arises from the missing mutant localization experiments that affect interpretation of yeast phenotypes. This is especially relevant for dimer mutants, which may be structurally disorganized and arrested in trafficking. It is important to distinguish between the effect of mutations on transporter biogenesis versus transport function.

Originality and Signficance: First, the structure reveals a unique mechanism of dimerization that involves domain swapping of an extra N-terminal helix with limited contact points. The latter provided the opportunity to query the role of dimerization in transport activity (with the caveat below). In other Na+/H+ exchangers, extensive molecular contacts between dimers make a mutagenic approach untenable.

Second, we clearly see the structural effect of non-canonical substitutions in the NHA2 ion-binding cavity, which result in lower negative charge compared to NapA. While MD simulations point to a lower ion-binding rate, likely corresponding to lower transport rates, this could potentially be regulated by pH, lipids or other molecular modifiers.

Third, importantly we now have a structural basis for the experimentally observed lack of electrogenic transport resulting from substitution of Arg for Lys in the salt bridge: the structure shows a 5 angstrom distance between Arg431 and Asp277. This observation, coupled with the high pKa of Arg relative to Lys strengthens the hypothesis that the equivalent Lys residue carries the second proton in electrogenic exchangers (rather than the first aspartate of the DD dyad). The molecular basis electrogenicity is a critical question in the Na/H antiporter field.

Fourth, using elegant SSM-based electrophysiology, the authors argue that NHA2 mediates H+ gradient driven Na+ efflux, complementing similar arguments in the literature based on biochemical approaches.

Fifth, the structure in nanodiscs reveals a hitherto unappreciated role for PI lipids in structural organization, dimerization and potential transport function of NHA2. The similarity of TM-1 to the extension helix in BetP leads the authors to speculate on a potential role for NHA2 in volume sensing, which would be consistent with its proposed role as a marker or regulator of hypertension and in polycystic kidney disease.

Suggested improvements:

Experimental (major): Information on subcellular localization of NHA2 constructs is missing. The extensive mutagenic analysis using salt tolerance phenotype in yeast is appreciated and adds essential functional relevance to the emergent structural insights. However, loss of complementation in yeast could be due to mislocalization. For example, in the dimer to monomer mutagenesis experiments, did you check plasma membrane localization? This could be addressed by imaging GFP-tagged constructs, with the caveat that ER membranes can closely track plasma membranes in yeast cells. Note that ER localization gives a characteristic “double ring” appearance (around the nucleus AND cell periphery).

Text (minor):
Page 3, line 65; fig 1b and legend. The statement that electroneutral NHE members (CPA1) are solely coupled to Na+ gradients is incorrect. While this is true for the plasma membrane subtype (NHE1-5), the intracellular subtype (NHE6-9) is driven by H+ gradients generated by V-ATPase, well documented

in endosomes. The structural basis for this preference, if any, is unclear to me and if the authors have any insight, I would love to hear it.

Figure 1c-d: TM-1 is labeled +1TM. This is confusing.

Line 345, page 11 (and elsewhere): Coupling of NHA2 to V-ATPase has been observed on the plasma membrane of MDCK cells. Similarly, NHA2 is highly expressed in osteoclasts that are well known for V-ATPase activity in ruffled membranes at the cell surface. Are the authors extrapolating this functional link to V-ATPase to lysosomes? I am not aware of any evidence to support a functional role of NHA2 in lysosomal membranes. Could the authors clarify?

Page 3 line 80: “missing a 69 amino acid tail deletion”. Missing and deletion are redundant.

Page 4, line 114: what is meant by “proceeding” here?

Reviewer #2:
Remarks to the Author:
Summary
Matsouka et al. (NSMB-A45211-T) presents single particle cryo-electron microscopy (cryo-EM) structures of the bison Sodium/Hydrogen Exchanger, biNHA2, in detergent micelles and lipid nanodiscs, supported by ex vivo and in vitro analysis to test hypotheses related to its structure and function. The hitherto unknown structure of biNHA2 shows an unanticipated number of transmembrane (TM) helices (based on homologous proteins and predictions) and significant conformational differences between detergent and lipid nanodisc conditions. Notably, the authors see an additional helix in the N-terminus (TM -1) and conformations that are reminiscent of the 12-TM antiporter NapA. Using solid-supported membrane (SSM)-based electrophysiology, the authors show that biNHA2 is an electroneutral transporter and functions as a homodimer.

The research presented in the manuscript is timely and relevant to the broader audience. NHA2 and its mammalian homologs are structurally underrepresented despite their physiological and pharmacological relevance. As such, the exact number of TM helices has also been a matter of debate in the field for several years. Finally, as the protein has few features other than TM helices and is a homodimer, it poses a particularly challenging and interesting target for single particle cryo-EM.

The experiments support the claims the authors make in large part and there is no doubt that a tremendous amount of work and well-designed experiments have gone into preparing the manuscript; however, the manuscript is difficult to follow throughout and comes off as rushed. I strongly recommend that the authors restructure the manuscript and unpack the data in such a way that it is more approachable for the general readership of the journal. I recommend publishing after minor revisions according to my comments below.

Major issues
1) The manuscript lacks structure and would benefit from section headings to guide the reader through the paper. For instance, most of the first paragraph (L 45-67) caterers to a very narrow readership: it is too long and takes the reader too far away from NHA2 and its broader relevance, instead it dwells at its family context. This does not serve the scope nor the readership of the journal.

Likewise, several figures in the main text, the Extended Data and Supplementary seem redundant and much of the story is lost within.
2) The manuscript contains only very few figures that showcases their single particle cryo-EM data that the bulk of the manuscript is based upon. The main figures are completely devoid of any cryo-EM maps, which instead is contained in a few of the Extended data figures outlining the data processing pipeline. Only models based on their data are shown in the main figures. This leaves the impression that the authors do not want to put their original data that most of their conclusions are based upon on display. As the cryo-EM structures make up a substantial proportion of the work, the authors should dedicate at least one or two panels in figure 1 and move the phylogenetic tree of NHE1-9 and the schematic of CPA1 members in the supplementary material.
3) As a homodimeric protein, the protein could have been processed with imposed symmetry (C2) but the authors decided not to, and processed it in C1. The authors should argue for their choice in context of the translocation mechanism of NHA2 and whether this is coupled between the two monomers. The functional state is a dimer, so how are the two monomers coupled? Even if this question remains elusive, it is part of the structure and molecular mechanism of NHA2 which the authors promise the reader to elaborate on in their introduction. It would be interesting to know if the resolution improved for the monomer when imposing C2 symmetry or when doing focused refinement on the monomer (e.g. through symmetry expansion)?
4) The manuscript presents two structures of NHA2: one in detergent and one in lipid nanodiscs. However, Table 1 presents three structures each with their own EMDB and PDB entry (the formatting of the table is off though). The distinction between the two detergent maps (before and after subtraction) should be clearer in the main text (L97) and explained further in the methods section. In particular as this is not done for the nanodisc structure.
5) It seems like data from the complementation assay is duplicated and included more than once, e.g. Supplementary Fig 3 panel a, b and c, and Extended Data Fig 2c. This should be made more concise with fewer plots.
6) The authors should substantiate their presentation of the MD simulations with functional data, e.g. in L144-147. Does functional data support these observations?
7) The conclusion that PI lipids are located at the dimerization interface and responsible for any of the observed conformational changes should be tested in nanodiscs that does not contain PI lipids. The thermostability screen is not enough to support this notion.
8) The authors should elaborate on the proposed Lithium sensitivity for NHA2 and its relevance to human health.

Minor issues
1) Several abbreviations are never introduced before use (many of these are even found in the abstract and then also later in the manuscript), making the manuscript difficult to read. Please revise and introduce these throughout.
• L26: SLC9B2, should be Solute Carrier Family 9 Member B2
• L30: cryo EM, should be single particle cryogenic electron microscopy (cryo-EM)
• L33: SSM, should be solid-supported membrane
• L49: NHE, should be Na+/H+ exchanger
• L50: SLC9A1-9, should be Solute Carrier Family 9 Member A1-9
• L71: WNK4-NCC, should be with-no-lysine kinase 4- sodium-chloride cotransporter
• L189: lipid-protein-ratio (LPR) is introduced twice and seems redundant.
• L284: GFP-based thermal-shift assay (GFP-TS) is introduced twice and seems redundant.
• L289: phosphoinositides (PI) is used before this line, but abbreviated here.
• Supplementary and Extended data figures: bison NHA2 is referred to as biNHA2, but only referred to

as such in the methods section, not in the main text. The authors should be more concise.
2) L25: "… activity that is carried out in most, if not all cells." This statement should be substantiated or rephrased. Either they are in some cells or in all.
3) L36: "… from the well-established ion-binding aspartates." Well-established based on what? The authors should make this clearer.
4) L42: Assays done in cells (in culture) is termed ex vivo, not in vivo. The authors should revise the manuscript accordingly.
5) L27, 53, 68: The interpretation of "Na+(Li+)" seems specific to the field of exchangers/antiporters and needs an introduction for the broader readership.
6) L57: "… structures that operate by an "elevator" alternating-access mechanism" this statement should be substantiated by more than a single reference.
7) L59: Reference 12 is listed twice.
8) L81: The authors should reference the software that they based their truncation upon "… reduce predicted disorder"
9) L83: Supplementary Fig 2 and Extended Data Fig 1 are very similar and should be combined into a single overview to convey their messages.
10) L98: Missing reference to cryoSPARC.
11) L102: The authors should refer the reader to a figure with a structural overview in addition to the methods section for context.
12) L108-110: "The additional N-terminal helix TM –1 is domain-swapped in the dimer domain, and mediates homodimerization by interacting with TM8 and the TM8-TM9 loop from its neighbouring protomer (Fig. 1d)." The authors should make this clearer.
13) L114-117: "… helix extension that is also highly positively-charged and present on the intracellular face". This reads odd. The authors are not observing a positively charged helix, but assigned this stretch to be a helix that is positively charged. The authors should revise.
14) L121: Fig. 1f does not exist.
15) L126-127: "In NHA2, a lysine residue Lys459 replaces the strictly-conserved arginine residue found in all 13-TM-members13". The authors should reference more than a single work and delete "a lysine residue". Also, they should remind the reader of the relevance of the mentioned residues.
16) L137: Why is there a section break here?
17) L137-139: "… position (Glu333) is important for initially attracting Na+ ions to the ion-binding aspartates …" Authors should substantiate this statement with references.
18) L173: The authors should reference PROPKA which was presumably used for the pKa prediction mentioned here.
19) L179: "To robustly rule out that Asp277 cannot act as a proton carrier…" This is a very odd sentence, and the authors should consider to rephrase.
20) L182: The authors should cite more than a single study to underline the significance of the SSM-based technique.
21) L270: The authors should remove "compelling".
22) L561-613: The methods section explaining the cryo-EM processing should be rewritten and made more concise. "Relion 3.0 or 3.1" is stated an unnecessary number of times.
a. L565-570: This section is written as if it applies to both data sets but is contained within the section on the detergent NHA2.
b. L576-578: This sentence seems like it was originally two sentences.
23) Readability would be greatly improved with schematic figures outlining the core concepts of SSM-based electrophysiology and the complementation assay.
24) It would be nice with an overview of the mutants used in the ex vivo assays mapped onto the structure for context.

25) Fig. 1: TM -1 is labelled "+1 TM helix" and should be corrected.
26) Extended Data Fig 2a. The authors should explain why the OD600 is the same for biNHA2ΔN is the same as D277C-D278C at 100 mM LiCl.
27) The authors should add a space between units and the numbers they belong to throughout the methods section.
28) mL should be with capital L. The authors should correct this.
29) Extended Data Fig 3 and 10. The authors should disclose the orientation distribution of their data.
30) Extended Data Fig 5a. The authors should consider including this overview in one of the main figures.
31) Supplementary Fig. 1a. "strain" is miss spelled.

Reviewer #3:
Remarks to the Author:
The manuscript by Matsuoka and colleagues presents the cryo EM structures of NHA2 in detergent and nanodisc. SSM-based electrophysiology experiments illustrate that the NHA2 is an electroneutral transporter. The tryptophan-arginine-glutamate triad is determined important for the ion selectivity. An additional N-terminal helix undergoes substantial conformational change in the two structures reported in this work. The authors speculate that this conformational change results from phosphatidylinositol lipid binding, although I am not sure about this conclusion due to some concerns on the cryo EM maps and models in the related regions (see below). The authors also performed MD stimulations to further support their findings; however, I am not an MD stimulation expert so I will not comment on the quality of those parts.

Major concerns：

1， Cryo-EM analysis
This work presents cryo-EM maps of NHA2 in detergent and nanodisc, with reported resolution at 3.0 and 3.5 Å, respectively. The map quality of the NHA2-nanodisc sample is reasonable and the map features are consistent with its reported 3.5 Å resolution. However, the NHA2-detergent map seems poorly resolved and even worse than the NHA2-nanodisc map. Most of the side chain densities are missing in the map. The NHA2-detergent map looks more like a 4-4.5 Å map, and its resolution may have been over-estimated. Please check if the mask for refinement is too tight or the dataset contains duplicated particles.

It's also very puzzling that the methods state that the model building was performed based on the lower quality of NHA2-detergent map, instead of the NHA2-nanodisc map. It appears to be more reasonable to

use NHA2-nanodisc map, which contains more side chain features.

2, Phosphoinositide lipid

The authors employed a GFP-based thermal shift assay to study thermal stability of the NHA2ΔN and NHA2ΔTM-1, in presence of various lipid molecules. In figure 3d, there is only one control (control DDM). Is this a heated or unheated control? It would be helpful to provide both controls. Please also comment that TM value determined by this method represent stability of dimerized NHA2ΔN or monomeric NHA2ΔN?

Based on figure 3d, the PIP2 can improve thermal stability of NHA2ΔTM-1 sample to a degree similar to or greater than that of NHA2ΔN. Since the NHA2ΔTM-1 exists as a monomer, the improvement of the thermal stability should result from the PIP2 binds to the monomeric NHA2ΔTM-1 instead of the dimer-dimer interface. However, the authors interpreted this experiment as that the PIP2 binds to and stabilizes the dimerization interface. On the other hand, phosphatidylinositol was not determined bound with NHA2 protomer in the NHA2-nanodisc map. Please comment on this discrepancy.

PIP2, instead of the PI, was confirmed to improve the stability of NHA2 according to the GFP-based thermal shift assay; however, the authors incorporated the PI rather PIP2 into nanodisc. Can the PI also improve the stability of the protein in GFP-based thermal shift assay?

The NHA2-nanodisc map seems to be better resolved than the NHA2-detergent structure, yet the density in dimerization interface does not seem to be sufficient to clearly define the identity of the PI head group. Please provide the density map for these PI head groups. Moreover, the polar PI head groups, including an inositol ring and a phosphate group, are completely buried in a hydrophobic cavity formed by two protomers, which would be energetically unfavored. Furthermore, the PI and its phosphorylated forms are primarily located in the inner leaflet of the PM. However, as shown in figure 4b, the authors placed two PI head groups in the dimerization interface at both intracellular and extracellular sides. How sure can the authors be that these lipid densities at the dimer interface belong to PI? It requires more comments to rationalize the positions of these head groups of PI that seemingly contradict with common knowledge.

3, Solid-supported membrane (SSM)-based electrophysiology recordings

Do the authors have any sense about the orientation of the NHA2 in liposomes? An estimation would be helpful in interpreting results from the liposome assay.

The KD value for a transporter-ligand pair depends on the conformation of the transporter, inward- or outward-facing. In general, different conformation results in distinct KD. Therefore, what is reported in the manuscript should be an apparent KD.

This work emphasizes the significance of the PI/PIP2. In the SSM based functional assay, the PI/PIP2 is not included, yet the transporter remains functional. How does one understand the functional roles of the putative PI in the dimer interface? And could the authors validate the putative remodeling roles of PI/PIP2 using the SSM based functional assay?

4, The proposed tryptophan-arginine-glutamate triad is buried in the core domain and far away from the putative ion binding site. However, the experiments suggest that they are important for the ion selectivity. Please provide brief discussion how this perturbation may happen.

Minor points:

1, line119, “Indeed, the NHA2 DN structure adopts a similar outward-facing conformation to NapA even if the homodimer is assembled very differently (Fig. 1f).” Fig. 1f is missing.

2, line 285, “Whilst the NHA2 TM-1 was found to be stabilised by all negatively-charged lipids, the NHA2 ΔTM-1 construct was selectively...”. Should NHA2 TM-1 be NHA2ΔN?

3, In figure 4b, a side view would be helpful to fully show the location of these PI head groups relatively to the plasma membrane.

4, In figure 4d, I speculate that the NHA2 detergent model contains an N terminal extended helix, which is absent in the NHA2 nanodisc structure shown in the right panel. If so, it may be unfair to compare conformational change of two structures of difference lengths; otherwise, the authors should mention this difference.

Is this additional helix at the N-terminus amphipathic? What’s the function of this helix the author would speculate? Has this helix been resolved in the nanodisc dataset? Please briefly discuss this.

Line 1104, legend of figure 4e should be for figure 4d.

5, The threshold of FSC between model and map is usually use 0.5 cutoff, not 0.143. Please correct related figures and table.

Author Rebuttal to Initial comments

We appreciate the positive response concerning our manuscript. We have carefully examined each remark and responded to all points below.

Reviewers' Comments:

Reviewer #1:
Remarks to the Author:
Summary: This original structure of a unique member of the mammalian superfamily of Na+/H+ exchangers, SLC9B2/NHA2, makes multiple new and significant contributions. The quality and quantity of data, and its presentation are exceptional. The literature has been nicely integrated into this study and appropriately referenced for maximal impact. My main caveat arises from the missing mutant localization experiments that affect interpretation of yeast phenotypes. This is especially relevant for dimer mutants, which may be structurally disorganized and arrested in trafficking. It is important to distinguish between the effect of mutations on transporter biogenesis versus transport function.

Originality and Significance: First, the structure reveals a unique mechanism of dimerization that involves domain swapping of an extra N-terminal helix with limited contact points. The latter provided the opportunity to query the role of dimerization in transport activity (with the caveat below). In other Na+/H+ exchangers, extensive molecular contacts between dimers make a mutagenic approach untenable.

Second, we clearly see the structural effect of non-canonical substitutions in the NHA2 ionbinding cavity, which result in lower negative charge compared to NapA. While MD simulations point to a lower ion-binding rate, likely corresponding to lower transport rates, this could potentially be regulated by pH, lipids or other molecular modifiers.

Third, importantly we now have a structural basis for the experimentally observed lack of electrogenic transport resulting from substitution of Arg for Lys in the salt bridge: the structure shows a 5 angstrom distance between Arg431 and Asp277. This observation, coupled with the high pKa of Arg relative to Lys strengthens the hypothesis that the equivalent Lys residue carries the second proton in electrogenic exchangers (rather than the first aspartate of the DD dyad). The molecular basis electrogenicity is a critical question in the Na/H antiporter field.

Fourth, using elegant SSM-based electrophysiology, the authors argue that NHA2 mediates H+ gradient driven Na+ efflux, complementing similar arguments in the literature based on biochemical approaches.

Fifth, the structure in nanodiscs reveals a hitherto unappreciated role for PI lipids in structural organization, dimerization and potential transport function of NHA2. The similarity of TM-1 to the extension helix in BetP leads the authors to speculate on a potential role for NHA2 in volume sensing, which would be consistent with its proposed role as a marker or regulator of hypertension and in polycystic kidney disease.

Thank you!

Suggested improvements:
Experimental (major): Information on subcellular localization of NHA2 constructs is missing. The extensive mutagenic analysis using salt tolerance phenotype in yeast is appreciated and adds essential functional relevance to the emergent structural insights. However, loss of complementation in yeast could be due to mislocalization. For example, in the dimer to monomer mutagenesis experiments, did you check plasma membrane localization? This could be addressed by imaging GFP-tagged constructs, with the caveat that ER membranes can closely track plasma membranes in yeast cells. Note that ER localization gives a characteristic "double ring" appearance (around the nucleus AND cell periphery).

Yeast cells expressing GFP-HsNHA2 showed mainly plasma membrane localization, but also within intracellular compartments in the B31 strain (ena1–4Δnha1Δ) (https://www.pnas.org/content/pnas/104/47/18677.full.pdf). In the Ab11c strain (ena1-4Δnhx1Δnha1Δ) we find that hsNHA2-GFP and bison NHA2-GFP predominantly localize to vacuoles and possibly endosomes with perhaps some signal at the plasma membrane (Supplementary Figure 4). From previous papers, we realize that although complementation has been carried out in the ABc11 strain, as far as we are aware, the confocal localization images are instead shown from the B31 strain; we do not have this strain to compare with.

The difference between Ab11c and B31 strains is the deletion of Nhx1. As you know, Nhx1 is the vacuolar Na+/H+ exchanger, which can also complement salt-stress by transporting Na+ into vacuoles https://www.molbiolcell.org/doi/10.1091/mbc.e04-11-0999. We think that in the absence of Nhx1, NHA2 is predominantly trafficked to vacuoles instead of the plasma membrane. Both plasma membrane and vacuolar locations will enable the cell to cope with saltstress.

Comparing the localization of bison and human NHA2-GFP, we find no obvious difference in trafficking compared to either the non-functional ion-binding bison NHA2-GFP mutants (D277C-D278C, E214A) or to the non-functional oligomerization mutants (TM-1 and D330AQ331A). From the localization data it's clear that the oligomerization mutants are not stuck in the ER. As a comparison, we further include confocal images from the KDEL receptor, which is often used as an ER marker. While vacuoles can also be sites for protein degradation, we find that oligomerization mutants are well-folded and see no signal for "free" GFP as one might expect from degradation.

Text (minor):
Page 3, line 65; fig 1b and legend. The statement that electroneutral NHE members (CPA1) are solely coupled to Na+ gradients is incorrect. While this is true for the plasma membrane subtype (NHE1-5), the intracellular subtype (NHE6-9) is driven by H+ gradients generated by VATPase, well documented in endosomes. The structural basis for this preference, if any, is unclear to me and if the authors have any insight, I would love to hear it.

Thank you. Ahem, yes, from our work on NHE9 we know this and we are not sure how the sentence ended up like this in the final version …

It's a bit too early to speculate, but the cavities of NHE1 are very negatively charged in both outward and inward facing states (Nature Communications 12: 3474 (2021)). However, NHE9, like NHA2, might only negatively charged in the inward-facing conformation (based on our unpublished outward-facing model). As such, the organellar NHEs might have an in-built ionbinding asymmetry.

Figure 1c-d: TM-1 is labeled +1TM. This is confusing.

Thank you. The +1 was "meant" to point out that NHA2 has 1 extra helix than the 13-TM members, but you are right this is, of course, confusing since the first helix was labelled TM-1. We have now corrected the figure with TM -1 instead.

Line 345, page 11 (and elsewhere): Coupling of NHA2 to V-ATPase has been observed on the plasma membrane of MDCK cells. Similarly, NHA2 is highly expressed in osteoclasts that are well known for V-ATPase activity in ruffled membranes at the cell surface. Are the authors extrapolating this functional link to V-ATPase to lysosomes? I am not aware of any evidence to support a functional role of NHA2 in lysosomal membranes. Could the authors clarify?

We have clarified that since NHA2 localizes to lysosomes that its "likely" to be important for pH homeostasis here. Indeed, the other NHE6(9) members are regulated differently via their large C-terminal tails, and so NHA2 "might" have a more house-keeping role. There is also the interesting implication of volume sensing since lysosomes change their volume dramatically.

We provide further data (native MS and further thermal-shift assays) supporting the specificity of NHA2 for PI and PIP2 lipids. This makes sense as phosphoinositides, compared to any other lipid-class, are well-known for their regulation of ion-channel and transporter activity (J Physiol. 2010 Sep 1; 588(Pt 17): 3179–3185). Indeed, the closing on the cytoplasmic gap in NHA2 by PI lipids is consistent with its preferential distribution in the inner leaflet and its preferential location in intracellular organelles (J Cell Biol. 2020 Mar 2; 219(3): e202001185.)

[EDITORIAL NOTE: Figure redacted to remove third-party material. Shown was Figure 1 panel (f) from https://doi.org/10.1083/jcb.202001185]

"+" – the plus sign corresponds to the relative abundance of PI lipids (J Cell Biol. 2020 Mar 2; 219(3): e202001185)

But, the analysis is not as "simple" as this, and it is complicated by the fact that NHA2 can also

be found at the plasma membrane, which has a very different lipid composition. Moreover, based on the NHA2 structure, the most likely PIP2 site is located between a positively-charged residues on the extracellular side of NHA2, but as we all know the preferential location of PIP2 4 in on the inner leaflet of the plasma membrane. Its possible that NHA2 only needs a small fraction of PIP2 in the absence of PI as a structural lipid, and there is just enough PIP2 on the outer leaflet for this, see e.g. BBRC 527:4,1050-1056. We also know that PIP2 lipids are important for basolateral to apical sorting in epithelial cells, which is where NHA2 needs to traffic to in kidney cells to function (Proc Natl Acad Sci U S A. 2014 May 27; 111(21): 7689–7694).

In addition to above, we clarify that all the lipid-binding sites in NHA2 are located next to tryptophan residues. Tryptophan is well-known to be important for anchoring TM segments in the membrane and to drive rearrangements of TMs in response to a change in membrane thickness, i.e., the hydrophobic mismatch. The lipid bilayer thickness is very different between organelles, and the plasma membrane and even between the basolateral and apical bilayers (Proc Natl Acad Sci U S A. 2004 Mar 23; 101(12): 4083–4088).

Taken together, we propose that PI lipids are an important driver of the lipid-remodeling, but with an added dimension that the membrane bilayer thickness itself can likely further fine-tune the homodimerization and stability of NHA2. Nevertheless, it will clearly take future studies to piece together how these factors ultimately come together to regulate NHA2 function.

Page 3 line 80: “missing a 69 amino acid tail deletion”. Missing and deletion are redundant.

This has now been updated.

Page 4, line 114: what is meant by “proceeding” here?

We have now rewritten this sentence and the meaning should be now clear

Reviewer #2:
Remarks to the Author:
Summary Matsouka et al. (NSMB-A45211-T) presents single particle cryo-electron microscopy (cryoEM) structures of the bison Sodium/Hydrogen Exchanger, biNHA2, in detergent micelles and lipid nanodiscs, supported by ex vivo and in vitro analysis to test hypotheses related to its structure and function. The hitherto unknown structure of biNHA2 shows an unanticipated number of transmembrane (TM) helices (based on homologous proteins and predictions) and significant conformational differences between detergent and lipid nanodisc conditions. Notably, the authors see an additional helix in the N-terminus (TM -1) and conformations that are reminiscent of the 12-TM antiporter NapA. Using solid-supported

membrane (SSM)-based electrophysiology, the authors show that biNHA2 is an electroneutral transporter and functions as a homodimer.

The research presented in the manuscript is timely and relevant to the broader audience. NHA2 and its mammalian homologs are structurally underrepresented despite their physiological and pharmacological relevance. As such, the exact number of TM helices has also been a matter of debate in the field for several years. Finally, as the protein has few features other than TM helices and is a homodimer, it poses a particularly challenging and interesting target for single particle cryo-EM.

The experiments support the claims the authors make in large part and there is no doubt that a tremendous amount of work and well-designed experiments have gone into preparing the manuscript; however, the manuscript is difficult to follow throughout and comes off as rushed. I strongly recommend that the authors restructure the manuscript and unpack the data in such a 5 way that it is more approachable for the general readership of the journal. I recommend publishing after minor revisions according to my comments below.

Thank you for your supportive comments and patience with this manuscript.

You are right, there is a competing paper and we had to write this up quickly, which was challenging as there is a lot of data. We agree that the manuscript was in needed of extensive reworking. We have modified the text, included subheadings, and added new figures that should now all make the paper easier to follow.

Importantly, we introduce the nanodisc NHA2 structure earlier in the manuscript, and the following analysis regarding the ion-binding site and MD simulations are now all based on the nanodisc NHA2 structure. Note, we cannot introduce the NHA2 structure at the same time as the detergent structure as i) the lipids used for the nanodisc structure are important and this required the lipid analysis that was carried prior to trialing nanodiscs, ii) part of the lipid analysis includes the comparison to the monomeric mutant TM-1, and we therefore also must introduce the section on oligomerization first too.

Major issues

1) The manuscript lacks structure and would benefit from section headings to guide the reader through the paper. For instance, most of the first paragraph (L 45-67) caterers to a very narrow readership: it is too long and takes the reader too far away from NHA2 and its broader relevance, instead it dwells at its family context. This does not serve the scope nor the readership of the journal. Likewise, several figures in the main text, the Extended Data and Supplementary seem redundant and much of the story is lost within.

We have re-written the introduction to keep the focus on the NHA2 physiology whilst still highlighting its evolutionary relationship to the NHEs. I think this works better and thank you for the suggestion. We have also re-made several figures to reduce redundancy and make the conclusions easier to follow.

2) The manuscript contains only very few figures that showcases their single particle cryoEM data that the bulk of the manuscript is based upon. The main figures are completely devoid of any cryo-EM maps, which instead is contained in a few of the Extended data figures outlining the data processing pipeline. Only models based on their data are shown in the main figures. This leaves the impression that the authors do not want to put their original data that most of their conclusions are based upon on display. As the cryo-EM structures make up a substantial proportion of the work, the authors should dedicate at least one or two panels in figure 1 and move the phylogenetic tree of NHE1- 9 and the schematic of CPA1 members in the supplementary material.

Thank you for your suggestion. After working on the structure of NHA2 for close to 8 years you can imagine that we are proud of the cryo EM maps. We are more than happy to include them in the main figures and have done as you have suggested. Please note that all the Extended Data figures will be available to follow online like main figures. It's just the Supplementary data that needs to be downloaded separately.

3) As a homodimeric protein, the protein could have been processed with imposed symmetry (C2) but the authors decided not to, and processed it in C1. The authors should argue for their choice in context of the translocation mechanism of NHA2 and whether this is coupled between the two monomers. The functional state is a dimer, so how are the two monomers coupled? Even if this question remains elusive, it is part of the structure and molecular mechanism of NHA2 which the authors promise the reader to elaborate on in their introduction. It would be interesting to know if the resolution improved for the monomer when imposing C2 symmetry or when doing focused refinement on the monomer (e.g. through symmetry expansion)?

Thank you for your feedback. The coupling we were referring to was how the monomers are assembled together, rather than the question if the transport domains show cooperative activity or not. We don't know if NHA2 shows cooperative activity, but we do know that the bacterial Na+/H+ antiporters do not, and these are also homodimers.

Many elevator proteins, such as GltPh (glutamate transporter; homotrimers) and CitS (citrate transporter; homodimers) show no clear co-operative activity. So why are Na+/H+ exchangers and other elevator proteins oligomers? The current thinking is that the scaffold domain needs to support the large movements of the core transport domains; indeed, these transitions have been directly observed by high-speed AFM for GltPh. The scaffold domain will be better anchored in a membrane if it is forming an oligomer. NHA2 is a useful system to test this rationale as the oligomerization interface is atypically

small, and therefore easier to manipulate; the two monomers are only joined together just between TM -1 of one protomer and TM8 and the TM7-TM8 loop of the other (Fig. 1c and Fig. 3c of initial submission). Indeed, just 2-point mutations in the TM7-TM8 loop domain is enough to disrupt NHA2 oligomerization and functional activity ex vivo. Having established that NHA2 is required to be an oligomer for maximal transport activity, this requirement then opens the possibility to regulate transport activity via controlling oligomerization. In NHA2, we see how oligomerization strength in detergent is stabilized by PI lipids, and we propose this is a regulatory switch. This conclusion is also influenced by the fact that other Na+/H+ exchangers, such as NhaA in E. coli, have evolved a dimerization interface that is dependent on cardiolipin for both oligomerization and in vivo functional activity.

Regarding cryo EM processing, we tried imposing C2 symmetry, but maps quality became worse in both the detergent and nanodisc structures. In NHE9, map quality also become worse when C2 symmetry was applied (EMBO J (2020)39:4541-4559). In both these structures, the map density and resolution were better for the dimerization domain and poorer for the core domains. This makes sense as the core domains are more dynamic. We think that the core domains may adopt further local differences, which means there is too much underlying asymmetry for imposing C2 symmetry.

Yes, we indeed used focused classification and refinement for the NHA2 detergent monomer and this improved map resolution in some regions that aided model building of the detergent NHA2 homodimer. In hindsight, we should have also included this information and maps in the initial submission. We have now deposited the focused NHA2 maps (TM1-13 of one monomer and TM-1 from the other monomer) and included this information to the cryo EM workflow description.

Yes, we also tried symmetry expansion of the NHA2 monomer, but this failed to improve map quality, which might be because of the noisy detergent background.

4) The manuscript presents two structures of NHA2: one in detergent and one in lipid nanodiscs. However, Table 1 presents three structures each with their own EMDB and PDB entry (the formatting of the table is off though). The distinction between the two detergent maps (before and after subtraction) should be clearer in the main text (L97) and explained further in the methods section. In particular as this is not done for the nanodisc structure.

Good point. We have made it clearer in the main text that masked refinement was used to improve map resolution, but the map density for the N-terminal extension helix was lost. We have made it clearer that we could not build the N-terminal extension helix into the nanodisc maps, as prior to masked refinement, we could not clearly distinguish between protein and nanodisc density in this region.

5) It seems like data from the complementation assay is duplicated and included more than once, e.g. Supplementary Fig 3 panel a, b and c, and Extended Data Fig 2c. This should be made more concise with fewer plots.

We agree. Extended Data Fig. 2 now show all mutants for LiCl complementation and the Supplementary Fig. 2 now has the complementary growth curves for LiCl. Bison NHA2 and D277C-D278C data are shown for every growth curve as we always repeat these measurements in parallel with the tested mutants, i.e., its not duplicated data. For those mutants further assessed with NaCl both growth curves and graphs have been combined into a single figure in Supplementary Fig. 2.

6) The authors should substantiate their presentation of the MD simulations with functional data, e.g. in L144-147. Does functional data support these observations?

With the re-written manuscript, I think these claims are clearer. In brief, MD simulation showed few spontaneously Na+ ion-binding site events, which is not surprising since the outside cavity is not very negatively charged in contrast to the outward facing states of NHE1 or NapA. Using the same MD simulation setup, we detect significantly many more ion-binding events to NapA than NHA2.

The inside surface of NHA2 is negatively charged and indicates that it would prefer Na+ binding in the inward-facing conformation. NHA2-GFP reconstitutes into liposomes with a 70% insideout orientation, which means the cytoplasmic side will be predominantly exposed to Na+ as in the physiological situation. Given the robust SSM signals and the apparent KD for Na+ like NHE1 (that has a very negatively charged extracellular surface), its logical to assume that NHA2 must be able to robustly take up Na+ from the cytoplasmic side.

7) The conclusion that PI lipids are located at the dimerization interface and responsible for any of the observed conformational changes should be tested in nanodiscs that does not contain PI lipids. The thermostability screen is not enough to support this notion.

We collaborated with Michael Landreh (Karolinska Institutet, Sweden) and Carol Robinson (Oxford, UK) to analyse the detergent-purified bison NHA2 sample by native MS. We find that 8 NHA2 monomers have no tightly bound lipids. In contrast, NHA2 dimers co-purify with 3 clear lipid adducts. One of these lipids has a molecular mass of 829 Da, which is closest to the average mass of PI from yeast at 850 Da (see Table below). To confirm this analysis, we carried out native MS of the 98% pure PI lipids used for nanodiscs, which shows that the second most abundant PI lipid has a mass of 836 Da, i.e., within error of the native MS peak. The other lipid has a molecular mass of 1036 Da, which almost certainly makes this the lipid PIP2 (1042 Da) as there are few lipids of this size.

[EDITORIAL NOTE: Table redacted to remove third-party material. Shown was Figure 1 panel (f) from https://doi.org/10.1101/gr.234503, Supplemental file 3, Table 7]

Previously we used the thermostability screen (GFP-TS) to show that NhaA was specifically stabilized by cardiolipin as first picked up by native MS. With this context in mind, we screened for thermostabilizing lipids by adding them individually to NHA2. Out of the 3 crude lipid mixtures added (yeast, brain and liver), only yeast polar lipids are clearly thermostabilizing. Yeast polar lipids are made up of close to 30% PI, whereas the PI content in the other crude lipids is reported to be less than 5% (our own lipidomics analysis calculates a PI content is less than 2%). This is important, as it implies NHA2 can select for PI in the presence of many other, competing lipids.

We now include thermostability data with 98% pure PI lipids that shows NHA2 is thermostabilized to a much higher degree than PC in DDM or DDM only. Although the delta Tm shift is only 8C for PI, we think this is an impressive amount of thermostabilization for a non-charged lipid and indicates a direct lipid-protein interaction. Indeed, as seen by FSEC, the addition of PI to detergent purified NHA2 significantly improves stability with or without heating for 10 mins at 40C.

We find that PIP2 breaks down a fraction of NHA2 homodimers into monomers after incubation on ice. We don't know if this is PIP2 lipids binding to less compacted NHA2 homodimer in the detergent mixture, or an artefact due to the relatively high concentrations of PIP2 lipids used in the assay compared to their natural abundance; thermal-shift assays require the ligand to be at a concentration 10-fold higher than the KD. This trend becomes more obvious after heating at 40C with PIP2 present. While it's unclear if this observation has any physiological implication, it nonetheless points out that the negatively-charged lipid can disrupt the dimer interface in NHA2, which means it must be binding there. This makes sense as PI conversely stabilizes the homodimer of NHA2 during heating. Furthermore, the TM-1 mutant is indiscriminately thermostabilized by all negatively-charged lipids. Taken together, this data supports our decision to make NHA2 nanodiscs with 98% pure PI lipids rather than PC lipids. This is also logical since PI lipids are well-known for regulating ion channels and transporter function compared to any other lipids.

The cryo EM maps show that PI headgroup coordinated next to strictly-conserved tryptophan residues. Statistically, tryptophan is 9-times more likely to coordinate sugar than any other 9 residue (J. Am. Chem. Soc. 2015, 137, 48, 15152–15160). The next most likely residue is aspartate and we find that a strictly-conserved aspartate from the TM-1 to TM1 loop that could potentially interact; unfortunately, we could not model this side-chain position. Overall, the structural data supports that the density located at the interface is from PI lipids, which makes sense since this was the only abundant lipid used in the nanodisc preparation.

Nonetheless, we have added a caveat that it's unclear if the lipid remodeling is specific to PI lipids only. This is something we will be following up in the future, perhaps by making nanodiscs with PC lipids etcetera for cryo EM work, but this will take some time. We have also changed the title to the following.

FROM: "Structure and lipid-mediated remodeling mechanism of the Na+/H+ exchanger NHA2"
TO "Structure, mechanism and lipid-mediated remodeling of the Na+/H+ exchanger NHA2".

Lipid preferences of NHA2 Fig 3

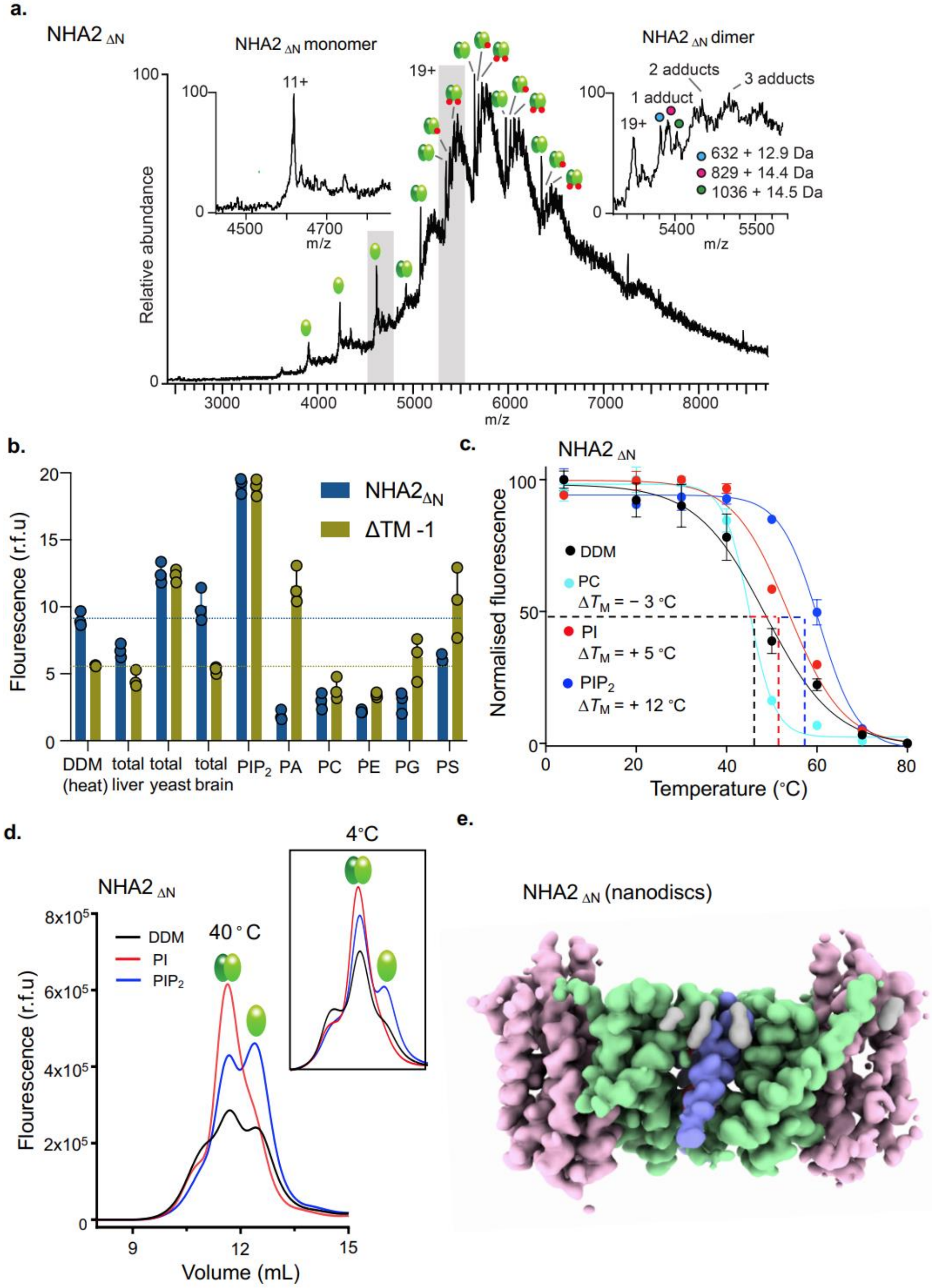

8) The authors should elaborate on the proposed Lithium sensitivity for NHA2 and its relevance to human health.

The increased Na+/Li+ exchange is a “phenotype” for hypertension and diabetes that is linked to NHA2, but the mechanistic role of lithium in human health is not understood much beyond the use for treatment for bipolar depression.

Respectively, as such, we have decided not to elaborate further on the text here, but we would also welcome input from Reviewer 1 if you think this is needed?

Minor issues
1) Several abbreviations are never introduced before use (many of these are even found in the abstract and then also later in the manuscript), making the manuscript difficult to read. Please revise and introduce these throughout.
• L26: SLC9B2, should be Solute Carrier Family 9 Member B2

Thank you. We have now spelled out SLC for its first-time use.

• L30: cryo EM, should be single particle cryogenic electron microscopy (cryo-EM)
Thank you, this has now been updated.

• L33: SSM, should be solid-supported membrane

Thank you, this has now been updated.

• L49: NHE, should be Na+/H+ exchanger

Yes, we can confirm we have checked that the first use of NHE has been spelled out in full (line 41).

• L50: SLC9A1-9, should be Solute Carrier Family 9 Member A1-9

Yes, thank you.

• L71: WNK4-NCC, should be with-no-lysine kinase 4- sodium-chloride cotransporter

We can confirm this has been updated.

• L189: lipid-protein-ratio (LPR) is introduced twice and seems redundant.

We can confirm that it's only been used once.

• L284: GFP-based thermal-shift assay (GFP-TS) is introduced twice and seems redundant.

We can confirm that it's only been used once

• L289: phosphoinositides (PI) is used before this line, but abbreviated here.

Thank you for spotting this. We have now updated.

• Supplementary and Extended data figures: bison NHA2 is referred to as biNHA2, but only referred to as such in the methods section, not in the main text. The authors should be more concise.

We have now clarified in the beginning that the *bison* NHA2 structural construct is referred to as NHA2[illegible]N and we no longer include bison in the main text.

2) L25: "… activity that is carried out in most, if not all cells." This statement should be substantiated or rephrased. Either they are in some cells or in all.

You are right, it's possible there are some cells that do not express Na+/H+ exchangers, but we have removed the sentence all together.

3) L36: "… from the well-established ion-binding aspartates." Well-established based on what? The authors should make this clearer.

We have re-written the abstract that had to be shortened for NSMB. We no longer refer to the ion-binding aspartates in the abstract.

4) L42: Assays done in cells (in culture) is termed ex vivo, not in vivo. The authors should revise the manuscript accordingly.

Thank you!

5) L27, 53, 68: The interpretation of "Na+(Li+)" seems specific to the field of exchangers/antiporters and needs an introduction for the broader readership.

We have now spelled out what is meant with Na+(Li+) in the beginning of the introduction.

6) L57: "… structures that operate by an "elevator" alternating-access mechanism" this statement should be substantiated by more than a single reference.

Good point. We now explain the "elevator " alternating-access mechanism in the introduction.

7) L59: Reference 12 is listed twice.

We could not see where reference 12 was listed twice. We have been through all the references and don't think we have missed this.

9) L81: The authors should reference the software that they based their truncation upon "… reduce predicted disorder"

OK.

9) L83: Supplementary Fig 2 and Extended Data Fig 1 are very similar and should be combined into a single overview to convey their messages.
Good point. We have now removed Supplementary Fig. 2 as its only there to show how high the sequence similarity was between human NHA1 and NHA2, but we can just as easily convey this information by quoting their sequence identity.

10) L98: Missing reference to cryoSPARC

Thank you for spotting this.

11) L102: The authors should refer the reader to a figure with a structural overview in addition to the methods section for context.

Thank you. This has now been updated.

12) L108-110: "The additional N-terminal helix TM –1 is domain-swapped in the dimer domain, and mediates homodimerization by interacting with TM8 and the TM8-TM9 loop from its neighbouring protomer (Fig. 1d)." The authors should make this clearer.

You are right. We tried differently labelling ideas, but in the end the most obvious is to colour each protomer with different colours. However, we wanted to label the different domains in the transporter too. We settled on including an insert in Fig. 1d with the different colours for the protomers, as we felt an additional figure was worth highlighting the unusual domain swapping.

13) L114-117: "… helix extension that is also highly positively-charged and present on the intracellular face". This reads odd. The authors are not observing a positively charged helix, but assigned this stretch to be a helix that is positively charged. The authors should revise.

Thank you. This sentence has been re-written.

14) L121: Fig. 1f does not exist.

This has now been corrected.

15) L126-127: "In NHA2, a lysine residue Lys459 replaces the strictly-conserved arginine residue found in all 13-TM-members13". The authors should reference more than a single work and delete "a lysine residue". Also, they should remind the reader of the relevance of the mentioned residues.

We have included some more references in addition to the large bioinformatic study we initially referenced. We think the proceeding sentences makes clear the relevance of the lysine residue.

"The characteristic feature of the "NhaA-fold" is the two discontinuous helices TM5a-b and TM12a-b in the 6-TM core ion-translocation domain, which contain unwound regions that cross-over each other near the centre of the membrane (Fig. 5b) 22,41. Proximal to these extended helix break-points are oppositely-charged residues that neutralize the half-helical ends of TM5a and TM12b dipoles (Fig. 5b)."

16) L137: Why is there a section break here?

The section has been re-written and so this break is no longer here.

17) L137-139: "… position (Glu333) is important for initially attracting Na+ ions to the ionbinding aspartates …" Authors should substantiate this statement with references.

This section has now been re-written and we no longer point out this detail explicitly.

18) L173: The authors should reference PROPKA which was presumably used for the pKa prediction mentioned here.

This statement comes from the work on arginine by Betrand's lab. We have included the reference and pasted abstract from the paper below:

Using complementary approaches of potentiometry and NMR spectroscopy, we have determined that the equilibrium acid dissociation constant (pKa value) of the arginine guanidinium group is 13.8 ± 0.1.

This is substantially higher than that of 12 often used in structure-based electrostatics calculations and cited in biochemistry textbooks. The revised intrinsic pKa value helps explains why arginine side chains in proteins are always predominantly charged, even at pH values as great as 10. The high pKa value also reinforces the observation that arginine side chains are invariably protonated under physiological conditions of near neutral pH. This occurs even when the guanidinium moiety is buried in a hydrophobic micro-environment, such as that inside a protein or a lipid membrane, thought to be incompatible with the presence of a charged group.

Protein Sci. 2015 May; 24(5): 752–761.

19) L179: "To robustly rule out that Asp277 cannot act as a proton carrier…" This is a very odd sentence, and the authors should consider to rephrase.

OK. We have rephrased the sentence to more simply state that "to confirm NHA2 is an electroneutral transporter …. i.e., valinomycin insensitivity is consistent with electroneutral activity, but is not proof of it.

20) L182: The authors should cite more than a single study to underline the significance of the SSM-based technique.

Good point. More references from Klaus's work have been added.

21) L270: The authors should remove "compelling".

Yes, this has now been re-written.

22) L561-613: The methods section explaining the cryo-EM processing should be rewritten and made more concise. "Relion 3.0 or 3.1" is stated an unnecessary number of times.

This section has now been edited more thoroughly.

a. L565-570: This section is written as if it applies to both data sets but is contained within the section on the detergent NHA2.

Thank you for spotting this error. It should have been written "NHA2 dataset" and not "NHA2 datasets

b. L576-578: This sentence seems like it was originally two sentences.

These sentences have now been re-written.

23) Readability would be greatly improved with schematic figures outlining the core concepts of SSM-based electrophysiology and the complementation assay.

We have now included a schematic figure for the yeast complementation assay (Supplementary Fig. 2a), and in the main text explained the core concept of the SSM-based electrophysiology method further.

24) It would be nice with an overview of the mutants used in the ex vivo assays mapped onto the structure for context.

We think that the individual figures highlight where all the residues are, better than an ensemble figure. But to better orient the reader, we have made a header on the ex vivo functional assays (Supplementary Fig. 2c-d).

25) Fig. 1: TM -1 is labelled “+1 TM helix” and should be corrected.

This has now been changed.
26) Extended Data Fig 2a. The authors should explain why the OD600 is the same for biNHA2ΔN is the same as D277C-D278C at 100 mM LiCl.

Under our culturing conditions, bison NHA2 can no longer complement salt-stress at 100 mM LiCl. The Abc11 strain itself can also no longer complement salt-stress. These results are consistent with previous results using the strain pasted below, but with slightly different culturing regime. In any case, 10 and 25 mM LiCl gives a clear phenotypic difference for comparing NHA2 mutants.

[EDITORIAL NOTE: Figure redacted to remove third-party material. Shown was Figure 3A from https://doi.org/10.1073/pnas.0707120104]

27) The authors should add a space between units and the numbers they belong to throughout the methods section.

OK!

28) mL should be with capital L. The authors should correct this.

OK!

29) Extended Data Fig 3 and 10. The authors should disclose the orientation distribution of their data.

We can confirm this information has now been added

30) Extended Data Fig 5a. The authors should consider including this overview in one of the main figures.

The topology figure will be just as accessible online as a main figure and so we have kept it here.

31) Supplementary Fig. 1a. “strain” is miss spelled.

OK!

Reviewer #3:
Remarks to the Author:
The manuscript by Matsuoka and colleagues presents the cryo EM structures of NHA2 in detergent and nanodisc. SSM-based electrophysiology experiments illustrate that the NHA2 is an electroneutral transporter. The tryptophan-arginine-glutamate triad is determined important for the ion selectivity. An additional N-terminal helix undergoes substantial conformational change in the two structures reported in this work. The authors speculate that this conformational change results from phosphatidylinositol lipid binding, although I am not sure about this conclusion due to some concerns on the cryo EM maps and models in the related regions (see below). The authors also performed MD stimulations to further support their findings; however, I am not an MD stimulation expert so I will not comment on the quality of those parts.

Major concerns:

1. Cryo-EM analysis This work presents cryo-EM maps of NHA2 in detergent and nanodisc, with reported resolution at 3.0 and 3.5 Å, respectively. The map quality of the NHA2-nanodisc sample is reasonable and the map features are consistent with its reported 3.5 Å resolution. However, the NHA2- detergent map seems poorly resolved and even worse than the NHA2-nanodisc map. Most of the side chain densities are missing in the map. The NHA2-detergent map looks more like a 4- 4.5 Å map, and its resolution may have been over-estimated. Please check if the mask for refinement is too tight or the dataset contains duplicated particles.

Thank you for this feedback.

We have included the mask used in refinement. We used a “soft” mask which generated from "relion_mask_create --extend_inimask 8 --width_soft_edge 8". We have registered the mask file on PDB and EMDB. For duplicated particles, we checked. The number of duplicated particles is 237 (0.088% in total) and it should not affect the FSC estimation.

As seen in Extended Data Fig. 4, many of the side-chains could be resolved in the detergent NHA2 maps. Is it possible that you looked at the cryo EM maps in the submission for the detergent_extension_helix structure, which was worse as these maps were prior to masked refinement? Nonetheless, it is an important point, and we have made much clearer now that one of the two protomers is better aligned and therefore the map density for one of the protomers was better. Furthermore, we carried out focused classification and refinement of the best protomer, which further improved map quality to guide side-chain positioning (TM1-13 from one monomer and TM -1 from the other monomer). We have now deposited these maps to the PDB and shown the side-by-side map density in Extended Data Fig. 4 (presented below).

We followed the FSC calculations in RELION by-the-book. We re-estimated the resolution in PHOENIX, but the estimates were similar (Acta Cryst. D74, 814-840 (2018)). The FSC calculation overestimates the quality of the NHA2 detergent maps and probably underestimates the quality for the nanodisc maps. We could add this example to the endless discussion on FSC interpretation and pitfalls on CCP-EM. After talking to Marta Carroni at our cryo EM facility about this, we think the most straightforward solution is to highlight the model resolution differences (FSC= 0.5) between the two structures. We have also enlarged the figures for the cryo EM maps between the nanodisc and detergent NHA2 proteins to make this clearer.

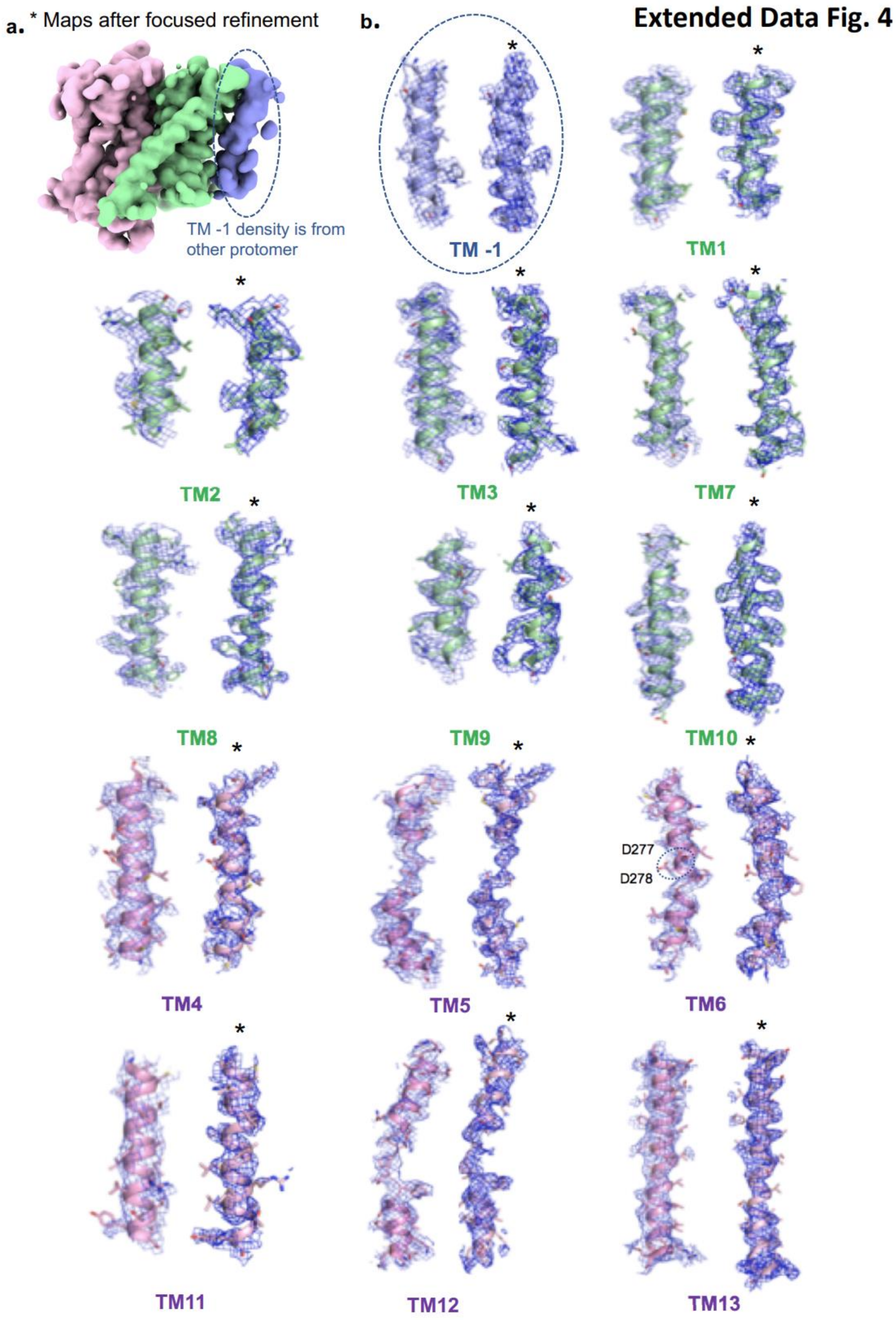
a. * Maps after focused refinement
TM -1 density is from other protomer
b.
Extended Data Fig. 4
TM -1
TM1
TM2
TM3
TM7
TM8
TM9
TM10
TM4
TM5
TM6
D277
D278
TM11
TM12
TM13

NHA2 detergent FSC in PHOENIX (FSC = 0.143; 2.94 Å)

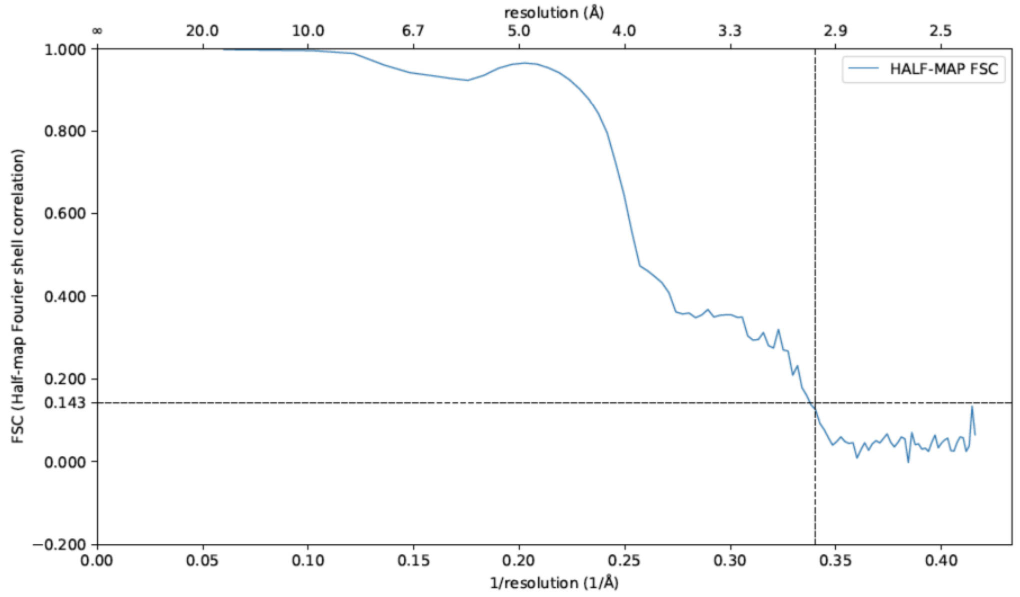

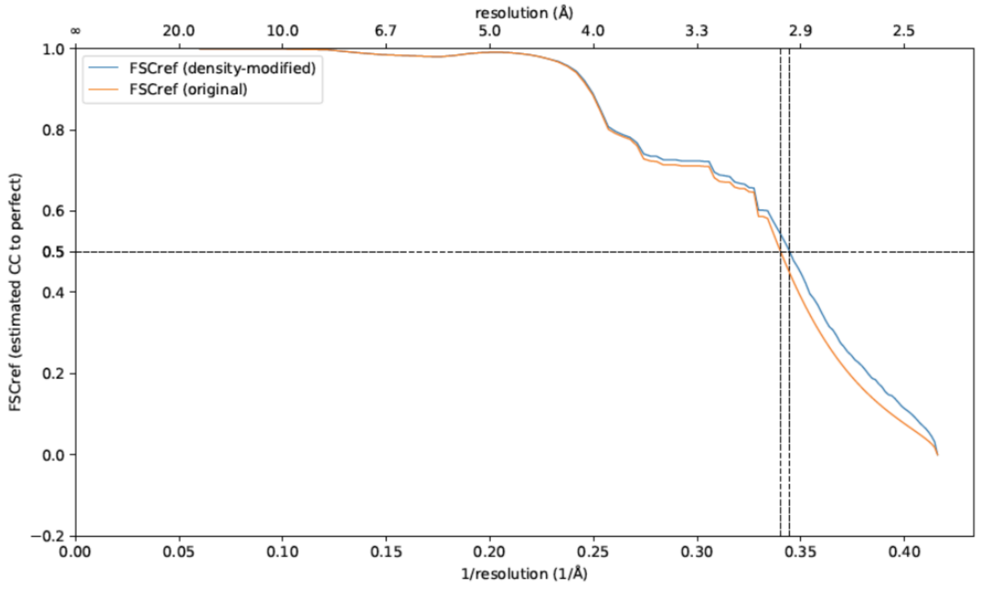

NHA2 nanodisc FSC in PHOENIX (FSC = 0.143; 3.61Å)

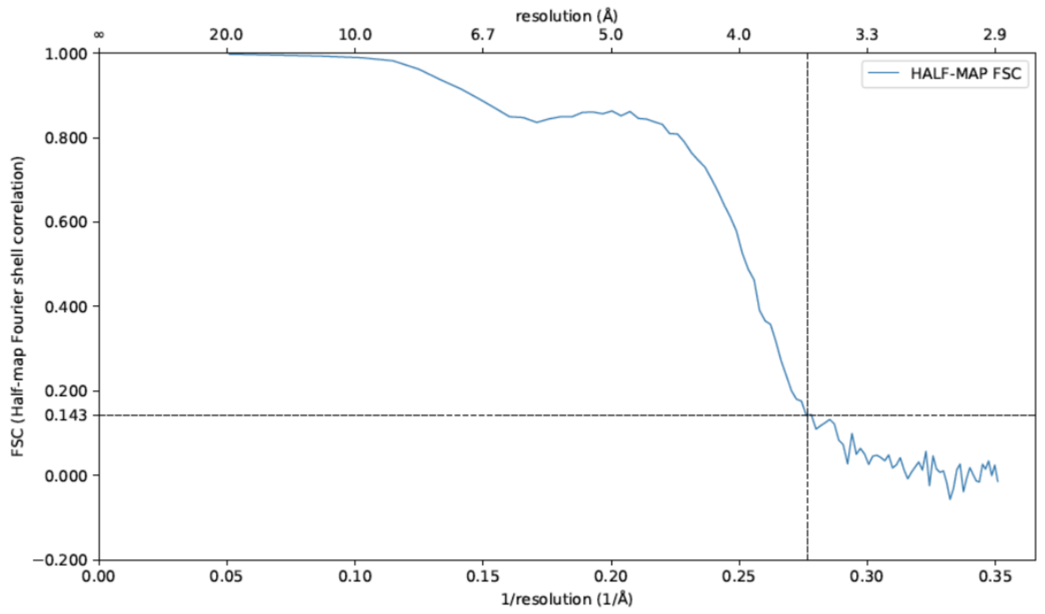

It's also very puzzling that the methods state that the model building was performed based on the lower quality of NHA2-detergent map, instead of the NHA2-nanodisc map. It appears to be more reasonable to use NHA2-nanodisc map, which contains more side chain features.

The paper was written in the order of our experimental efforts and we built the detergent NHA2 structure close to 2 years ago. Although some side-chains could not be resolved in the detergent NHA2 structure, almost all residues had been assigned correctly. Nonetheless, you are right, one of the lysine residues (Lys459) in the detergent NHA2 structure was repositioned based on the nanodisc structure and this information should have been included the previous submission.

We have now rearranged the order the manuscript to introduce the nanodisc NHA2 structure, before we describe details of the ion-binding site. The MD simulations are all based on the NHA2 nanodisc structure.

2, Phosphoinositide lipid The authors employed a GFP-based thermal shift assay to study thermal stability of the NHA2N and NHA2TM-1, in presence of various lipid molecules. In figure 3d, there is only one control (control DDM). Is this a heated or unheated control? It would be helpful to provide both controls. Please also comment that TM value determined by this method represent stability of dimerized NHA2N or monomeric NHA

Thank you. We have clarified that the DDM sample is the heated control. We include the Tm values for both the DDM/CHS purified bison NHA2 (47C) (Figure 3b) and the NHA2 TM -1 (57C) (Supplementary Figure 1c).

. Based on figure 3d, the PIP2 can improve thermal stability of NHA2TM-1 sample to a degree similar to or greater than that of NHA2N. Since the NHA2TM-1 exists as a monomer, the improvement of the thermal stability should result from the PIP2 binds to the monomeric NHA2TM-1 instead of the dimer-dimer interface. However, the authors interpreted this experiment as that the PIP2 binds to and stabilizes the dimerization interface. On the othe 21 phosphatidylinositol was not determined bound with NHA2 protomer in the NHA2-nanodisc map. Please comment on this discrepancy.

That's a good question. We have updated the text and include more data to explain this.

Please see answer to Referee 2 regarding PI lipids.

PIP2, instead of the PI, was confirmed to improve the stability of NHA2 according to the GFPbased thermal shift assay; however, the authors incorporated the PI rather PIP2 into nanodisc. Can the PI also improve the stability of the protein in GFP-based thermal shift assay?

Yes, we now show the thermostability with 98% pure PI lipids. With the concentrations of PIP2 lipids required for nanodiscs preparation we think these will de-stabilise the homodimer, which we see in FSEC traces. From a feasibility point of view, PIP2 is also very expensive.

The NHA2-nanodisc map seems to be better resolved than the NHA2-detergent structure, yet the density in dimerization interface does not seem to be sufficient to clearly define the identity of the PI head group. Please provide the density map for these PI head groups.

We apologise that the presented cryo EM maps did not make this clear and we have remade ALL these figures. We see clear cryo EM map density next to tryptophan residues that is too large for a lipid tail. Given that the nanodiscs were made with 98% pure PI lipids and given tryptophan likes to cooordinate sugar, we think can confidently model the sugar headgroup into these maps. As shown below, the signal for the two out of the four PI lipids (one shown below) is stronger than the neighboring tryptophan W171, which would not be consistent if this density was the result of a non-polar and dynamic fatty-acid tail.

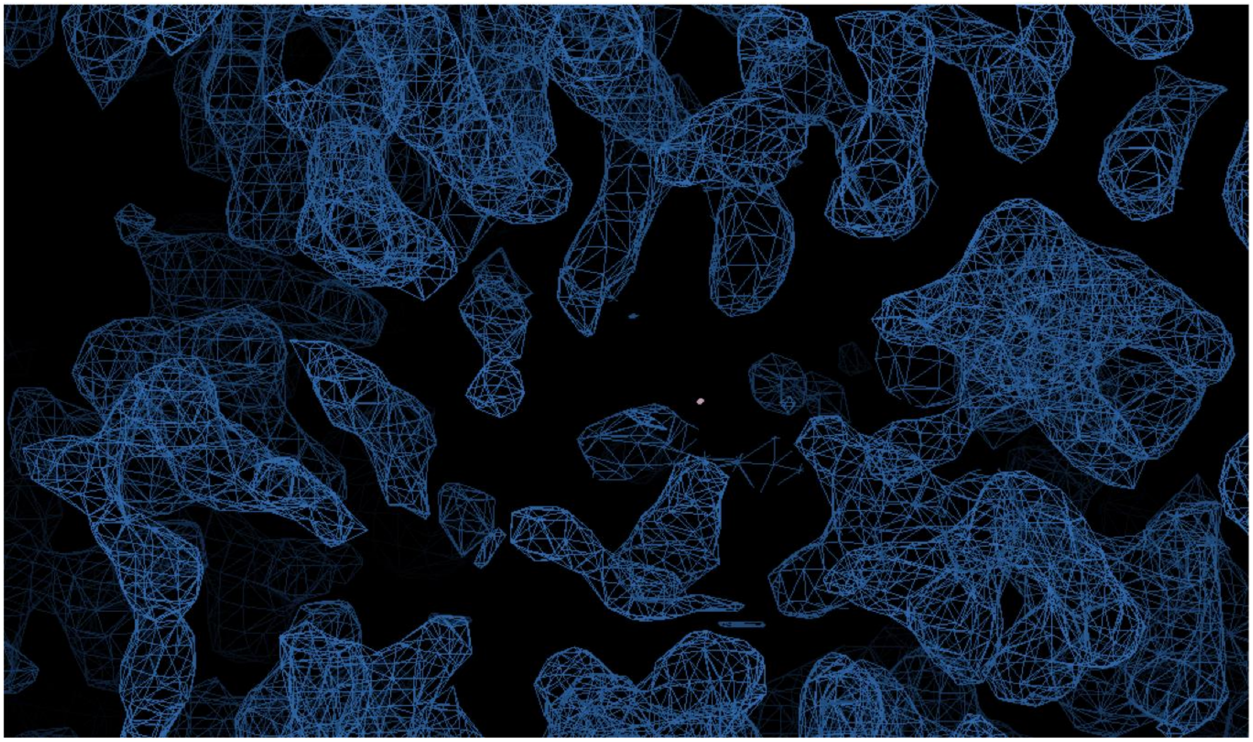

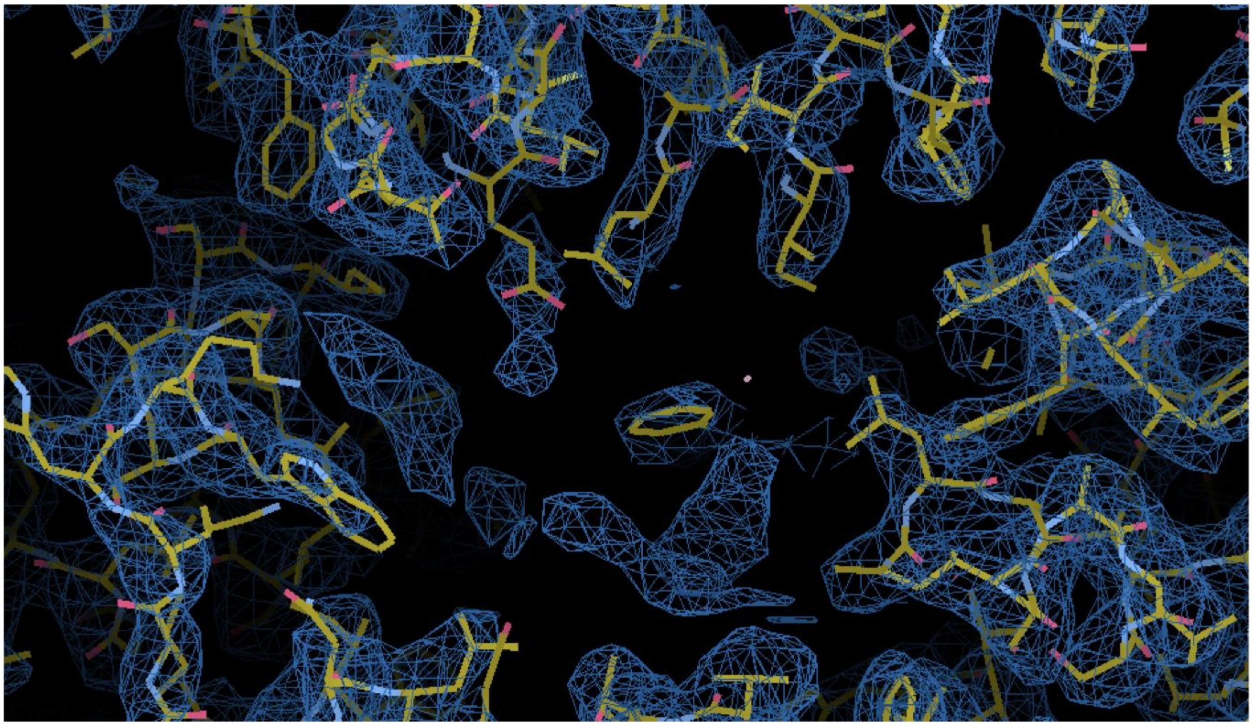

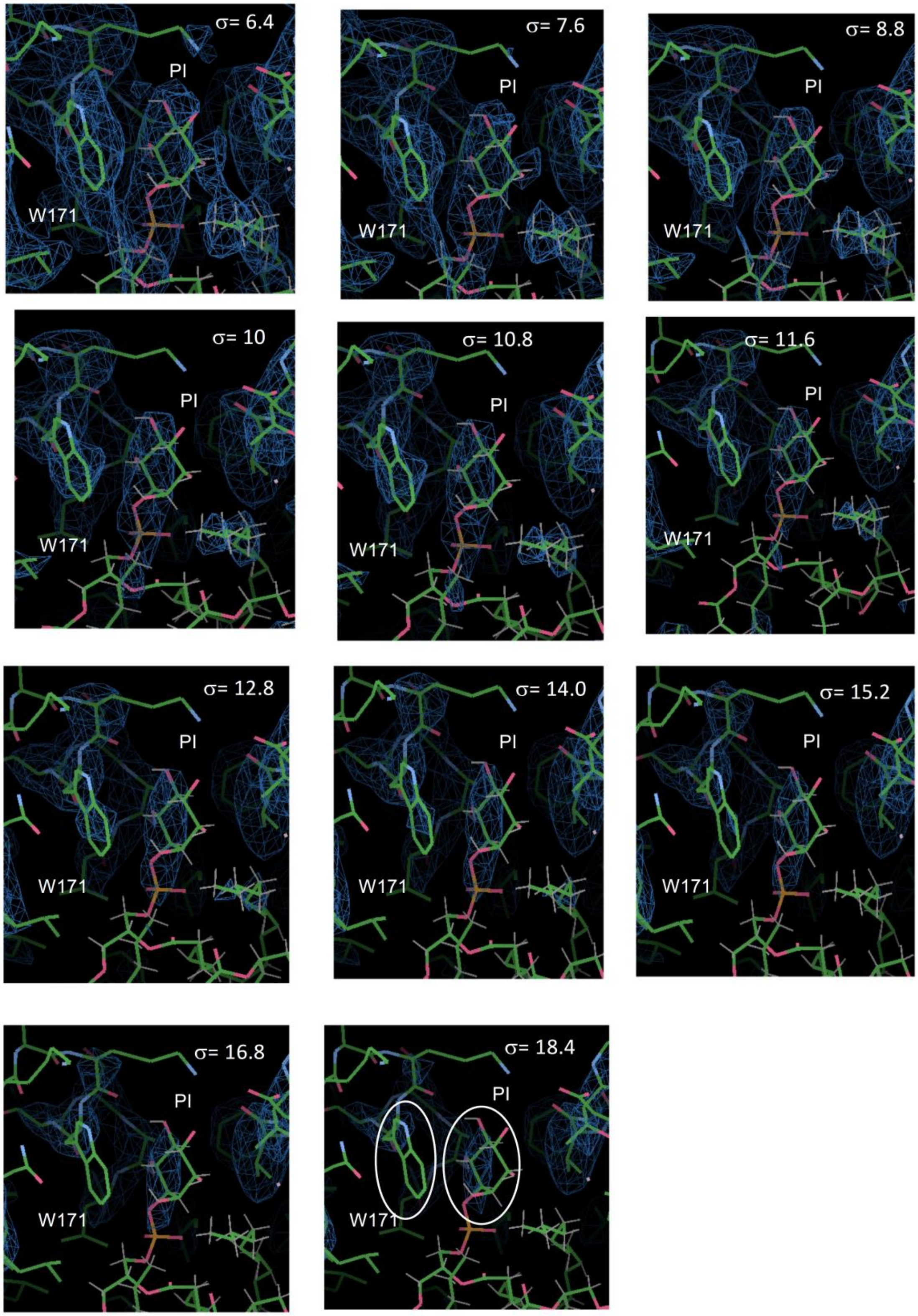
σ= 6.4
PI
W171
σ= 7.6
PI
W171
σ= 8.8
PI
W171
σ= 10
PI
W171
σ= 10.8
PI
W171
σ= 11.6
PI
W171
σ= 12.8
PI
W171
σ= 14.0
PI
W171
σ= 15.2
PI
W171
σ= 16.8
PI
W171
σ= 18.4
PI
W171

Moreover, the polar PI head groups, including an inositol ring and a phosphate group, are completely buried in a hydrophobic cavity formed by two protomers, which would be energetically unfavored.

It is well-known that tryptophan residues are enriched at the air-water interface and are important for anchoring TM segments in a membrane bilayer. We have re-made all the figures and made it much clearer in the text that all the lipid sites are next to tryptophan residues and are at located at the membrane interface. The phosphate groups represent the phosphodiester phosphate (not a free negatively-charged phosphate) and we have made this clearer by connecting the fatty acid tails for two of the PI lipids. We have left the 2 other PI lipids unconnected, as we don't feel the density is good enough to connect them.

Furthermore, the PI and its phosphorylated forms are primarily located in the inner leaflet of the PM. However, as shown in figure 4b, the authors placed two PI head groups in the dimerization interface at both intracellular and extracellular sides. How sure can the authors be that these lipid densities at the dimer interface belong to PI? It requires more comments to rationalize the positions of these head groups of PI that seemingly contradict with common knowledge.

As we have mentioned, nanodiscs were made from 98% pure PI and the size, shape and location is consistent with the headgroup of a sugar lipid. Whilst PIP2 is a signaling lipid typically found in the inner membrane of the plasma membrane, recent data indicates this is not always the case and PIP2 can also be found in the outer leaflet of the plasma membrane (https://www.sciencedirect.com/science/article/pii/S0006291X20309487).

PI has a very different membrane distribution to PIP2 and is not present in the inner leaflet of the plasma membrane, but is more enriched in intracellular organelles. The distribution between inner and outer leaflets within these organells is also unclear. This review highlights that while lipids like PI have a favored inner leaflet distributiom, there is increasing evidence that PI (like PS) can switch to the outer leaflet to act as a signal https://www.frontiersin.org/articles/10.3389/fcell.2016.00155/full

Nonetheless, in regards to the closing of the cytoplasmic gap in NHA2, the binding of PI to the inside would be consistent with the likely preferred inner leaflet of intracellular organelles. The PI lipids on extracellular side, however, are better coordinated and we have clarified this would be the most likely site for a PIP2 lipid due to the presence of positively charged residues that could coordinate phosphates. While we totally agree that this is against the "common knowledge" i) as stated, PIP2 can be present in the outer leaflet in the plasma and ii) we also find PIP2 binds to an extracellular loop domain in the endosomal Na+/H+ exchanger NHE9.

3, Solid-supported membrane (SSM)-based electrophysiology recordings Do the authors have any sense about the orientation of the NHA2 in liposomes? An estimation would be helpful in interpreting results from the liposome assay.

This is a good point. Based on TEV cleavage efficiency of NHA2-GFP incorporated into liposomes we estimate a 70% inside-out orientation. The biased orientation would explain the apparent KD estimates despite the poorly-charged negative surface on the outside.

The KD value for a transporter-ligand pair depends on the conformation of the transporter, inward- or outward-facing. In general, different conformation results in distinct KD. Therefore, what is reported in the manuscript should be an apparent KD.

OK! This has now been updated.

This work emphasizes the significance of the PI/PIP2. In the SSM based functional assay, the PI/PIP2 is not included, yet the transporter remains functional. How does one understand the functional roles of the putative PI in the dimer interface? And could the authors validate the putative remodeling roles of PI/PIP2 using the SSM based functional assay?

For SSM measurements, NHA2 was reconstituted into yeast polar lipids, which contain both PI and PIP2 lipids. To provide the direct link to functional activity is not easy. We would need to incorporate NHA2 into synthetic liposomes (POPC) that have been made with and without PI and PIP2 and compare SSM signals. Of course, even if we see increased signals, we would not know for sure that this just is not just because NHA2 is more stable in these liposomes. On the other hand, the i) specific thermostabilization we see with PI ii) the co-purification detected wit native MS and iii) the compacted NHA2 in the nanodisc structure stabilizing the homodimer and iv) that the homodimer is required for maximal NHA2 transport activity, is fairly strong evidence that PI is linked to NHA2 activity. Indeed, PI lipids are well known regulators of transporter and channel function.

We agree that a full appreciation and understanding of the role lipids play in NHA2 will require more data, but this could take several years to reach a firm conclusion (with our work on GLUT transporters it has taken 4-5 years to reach a conclusion of what lipids are required for optimal GLUT functional activity and we are still not ready to publish this).

4, The proposed tryptophan-arginine-glutamate triad is buried in the core domain and far away from the putative ion binding site. However, the experiments suggest that they are important for the ion selectivity. Please provide brief discussion how this perturbation may happen.

We have included a sentence of how this may happen. The tryptophan residue is like the π−bulge seen in the breakpoint helix of Na+/H+ antiporter-like subunits of complex I. In complex I, the tryptophan can form π−cation interactions with lysine. We have added a sentence that we think the tryptophan might form π−cation interactions with lysine in the Na+-bound state, but an ion-bound structure is needed to piece together the coupling.

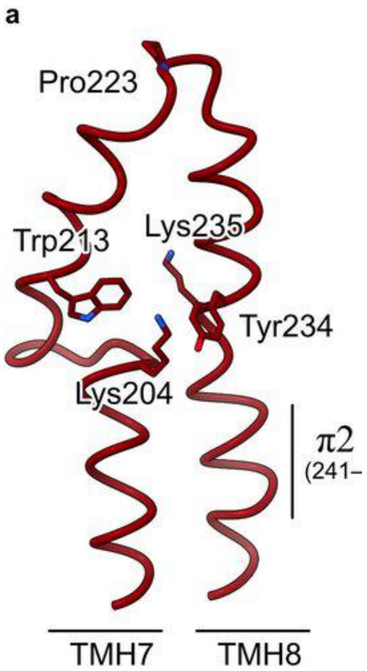

Nature Structural & Molecular Biology 27(10):1-9

Minor points:

1, line119, “Indeed, the NHA2 DN structure adopts a similar outward-facing conformation to NapA even if the homodimer is assembled very differently (Fig. 1f).” Fig. 1f is missing.

Thank you, this has now been updated.

2, line 285, “Whilst the NHA2 TM-1 was found to be stabilised by all negatively-charged lipids, the NHA2 TM-1 construct was selectively...”. Should NHA2 TM-1 be NHA2N

Thank you, this has now been updated.

3, In figure 4b, a side view would be helpful to fully show the location of these PI head groups relatively to the plasma membrane.

Agreed we have added this to Supplementary Figure 8c.

4, In figure 4d, I speculate that the NHA2 detergent model contains an N terminal extended helix, which is absent in the NHA2 nanodisc structure shown in the right panel. If so, it may be unfair to compare conformational change of two structures of difference lengths; otherwise, the authors should mention this difference.

We have clarified in the main text that it is the same length protein (construct) in both detergent and nanodisc structures, but prior to masked refinement we could not distinguish between extension and nanodisc map density to build this helix. Nevertheless. we think it's possible that this movement of the extension helix could also be contributing to the rearrangement and this is something we want to explore in the future.

Is this additional helix at the N-terminus amphipathic? What's the function of this helix the author would speculate? Has this helix been resolved in the nanodisc dataset? Please briefly discuss this.

Yes, there appears to be several positive charged residues on the helix to embed into membrane interface. Given the connection to TM-1 and the mobility of TM-1 we have speculated that this extension helix may contribute to NHA2 lipid-remodelling in response to volume sensing,

Line 1104, legend of figure 4e should be for figure 4d.

Thank you, this has now been re-written.

5, The threshold of FSC between model and map is usually use 0.5 cutoff, not 0.143. Please correct related figures and table.

Thank you, this has now been re-written.

Decision Letter, first revision:

1st Nov 2021

Dear David,

Thank you for submitting your revised manuscript "Structure, mechanism and lipid-mediated remodelling of the mammalian Na+/H+ exchanger NHA2" (NSMB-A45211A). It has now been seen by the original referees and their comments are below. I would like to note that we also cross-consulted reviewer #2 on the comment by reviewer #3. The reviewers find that the paper has improved in revision, and we agree with reviewer #2 that the concerns about the quality of the NHA2 detergent map have been addressed adequately. Therefore we'll be happy in principle to publish the paper in Nature Structural & Molecular Biology, pending minor revisions to satisfy the referees' final requests and to comply with our editorial and formatting guidelines.

We are now performing detailed checks on your paper and will send you a checklist detailing our editorial and formatting requirements in about a week. Please do not upload the final materials and

make any revisions until you receive this additional information from us.

To facilitate our work at this stage, we would appreciate if you could send us the main text as a word file. Please make sure to copy the NSMB account (cc'ed above).

Thank you again for your interest in Nature Structural & Molecular Biology Please do not hesitate to contact me if you have any questions.

Kind regards,
Florian

Florian Ullrich, Ph.D.
Associate Editor
Nature Structural & Molecular Biology
ORCID 0000-0002-1153-2040

Reviewer #1 (Remarks to the Author):

The authors have appropriately addressed the concerns from the previous review. A few minor typos/edits may be needed. For example, Abstract, line 1: NHA2 (gene name SLC9B2) correlates with..

Line 93 and others: strain name is correctly written as AB11c

Line 142 omit the phrase ex vivo, does not apply/make sense. Enough to say Li-salt stress with yeast growth, etc; transport-inactive would be a better way instead of dead.

(For the authors' information only: overexpression using a very strong promoter like GAL is good for protein production and purification but results in protein non-specifically ending up everywhere. I wouldn't bet on vacuolar localization from those images. A weak promoter is best for functional complementation with membrane proteins. The broad distribution of NHA2 across multiple organelles is more likely an issue with the promoter than the background strain.)

Reviewer #2 (Remarks to the Author):

I have now read the revised manuscript “Structure, mechanism and lipid-mediated remodelling of the mammalian Na+/H+ exchanger NHA2” by Matsouka et al. (NSMB-A45211-T) as well as the comments to

my concerns and those raised by the other two reviewers: I think that the authors have addressed our concerns, and it is my opinion that the manuscript is now much more appropriate for publication in Nature Structural & Molecular Biology.

I have a few minor comments that the authors should note:

Line 218-220: “Arg176 to alanine” should be Arg176Ala; “alanine mutant of His169” should be His169Ala to follow convention other places in the manuscript.

Throughout the manuscript: Missing hyphen in cryo EM. Please use cryo-EM to follow convention.

Reviewer #3 (Remarks to the Author):

The authors have answered most of my questions.
The only thing I am still concerned about is the map quality of NHA2 in detergent is not as good as they claimed (3 Å).It is more like a 4.0 Å map based on the density feature. The author confirmed that the particle set has few duplicates and the FSC curve also looks normal to me. So, it is really up to the editors.

Reviewer #2 (cross-consulted on comment by reviewer #3):

While I agree with Reviewer #3 that the map of NHA2 in detergent seems to have a much better reported resolution (3 Å) than it actually has in terms of details upon visual inspection, the authors have included all the necessary details on how they were able to get to this resolution: The authors confirm that the particle set has few duplicates and the [gold-standard] FSC curve looks normal.

The topic of estimated resolution of single-particle cryo-EM maps is a much debated one, however, the authors are doing it according to the currently accepted standards in the field. I don't think a potentially overfitted map should prevent the study from being published, particularly if all reviewers agree that the level of detail contained in the map is enough to substantiate their conclusions - particularly upon comparison with the nanodisc map which is of higher resolution (also upon inspection).

The ideal scenario would be that the authors deposit their entire particle stacks (from early on in their processing pipeline) to the EMDB to allow other researchers to process their data and see if they can reproduce the reported results.

Author Rebuttal, first revision:

Reviewer #1
(Remarks to the Author):

The authors have appropriately addressed the concerns from the previous review. A few minor typos/edits may be needed. For example, Abstract, line 1: NHA2 (gene name SLC9B2) correlates with..

Line 93 and others: strain name is correctly written as AB11c

Thank you. This has been updated.

Line 142 omit the phrase ex vivo, does not apply/make sense. Enough to say Li-salt stress with yeast growth, etc; transport-inactive would be a better way instead of dead.

Thank you. This has been updated

(For the authors' information only: overexpression using a very strong promoter like GAL is good for protein production and purification but results in protein non-specifically ending up everywhere. I wouldn't bet on vacuolar localization from those images. A weak promoter is best for functional complementation with membrane proteins. The broad distribution of NHA2 across multiple organelles is more likely an issue with the promoter than the background strain.)

Thank you, I agree that could also have contributed to the broader distribution.

Reviewer #2 (Remarks to the Author):

I have now read the revised manuscript "Structure, mechanism and lipid-mediated remodelling of the mammalian Na+/H+ exchanger NHA2" by Matsouka et al. (NSMB-A45211-T) as well as the comments to my concerns and those raised by the other two reviewers: I think that the authors have addressed our concerns, and it is my opinion that the manuscript is now much more appropriate for publication in Nature Structural & Molecular Biology.

I have a few minor comments that the authors should note:

Line 218-220: "Arg176 to alanine" should be Arg176Ala; "alanine mutant of His169" should be His169Ala to follow convention other places in the manuscript.

Thank you, this has now been updated.

Throughout the manuscript: Missing hyphen in cryo EM. Please use cryo-EM to follow convention.

Thank you, this has now been updated.

Reviewer #3 (Remarks to the Author):

The authors have answered most of my questions. The only thing I am still concerned about is the map quality of NHA2 in detergent is not as good as they claimed (3 Å).It is more like a 4.0 Å map based on the density feature. The author confirmed that the particle set has few duplicates and the FSC curve also looks normal to me. So, it is really up to the editors.

We agree, but we do not see it as a question of us “claiming” a resolution, but what the FSC curve calculates for our data.

Reviewer #2 (cross-consulted on comment by reviewer #3):

While I agree with Reviewer #3 that the map of NHA2 in detergent seems to have a much better reported resolution (3 Å) than it actually has in terms of details upon visual inspection, the authors have included all the necessary details on how they were able to get to this resolution: The authors confirm that the particle set has few duplicates and the [gold-standard] FSC curve looks normal.

The topic of estimated resolution of single-particle cryo-EM maps is a much debated one, however, the authors are doing it according to the currently accepted standards in the field. I don't think a potentially overfitted map should prevent the study from being published, particularly if all reviewers agree that the level of detail contained in the map is enough to substantiate their conclusions - particularly upon comparison with the nanodisc map which is of higher resolution (also upon inspection).

Thank you for the pragmatic response to this query.

The ideal scenario would be that the authors deposit their entire particle stacks (from early on in their processing pipeline) to the EMDB to allow other researchers to process their data and see if they can reproduce the reported results.

Yes, this is certainly something we will look into doing.

Final Decision Letter:

natureresearch

14th Dec 2021

Dear David,

We are now happy to accept your revised paper "Structure, mechanism and lipid-mediated remodelling of the mammalian Na+/H+ exchanger NHA2" for publication as a Article in Nature Structural & Molecular Biology.

Acceptance is conditional on the manuscript's not being published elsewhere and on there being no announcement of this work to the newspapers, magazines, radio or television until the publication date in Nature Structural & Molecular Biology.

Over the next few weeks, your paper will be copyedited to ensure that it conforms to Nature Structural & Molecular Biology style. Once your paper is typeset, you will receive an email with a link to choose the appropriate publishing options for your paper and our Author Services team will be in touch regarding any additional information that may be required.

After the grant of rights is completed, you will receive a link to your electronic proof via email with a request to make any corrections within 48 hours. If, when you receive your proof, you cannot meet this deadline, please inform us at rjsproduction@springernature.com immediately.

You will not receive your proofs until the publishing agreement has been received through our system.

Due to the importance of these deadlines, we ask that you please let us know now whether you will be difficult to contact over the next month. If this is the case, we ask you provide us with the contact information (email, phone and fax) of someone who will be able to check the proofs on your behalf, and who will be available to address any last-minute problems.

To assist our authors in disseminating their research to the broader community, our SharedIt initiative provides all co-authors with the ability to generate a unique shareable link that will allow anyone (with or without a subscription) to read the published article. Recipients of the link with a subscription will also be able to download and print the PDF.

As soon as your article is published, you can generate your shareable link by entering the DOI of your article here: <a href="http://authors.springernature.com/share">http://authors.springernature.com/share<a>. Corresponding authors will also receive an automated email with the shareable link

Note the policy of the journal on data deposition:

http://www.nature.com/authors/policies/availability.html.

Your paper will be published online soon after we receive proof corrections and will appear in print in the next available issue. You can find out your date of online publication by contacting the production team shortly after sending your proof corrections. Content is published online weekly on Mondays and Thursdays, and the embargo is set at 16:00 London time (GMT)/11:00 am US Eastern time (EST) on the day of publication. Now is the time to inform your Public Relations or Press Office about your paper, as they might be interested in promoting its publication. This will allow them time to prepare an accurate and satisfactory press release. Include your manuscript tracking number (NSMB-A45211B) and our journal name, which they will need when they contact our press office.

About one week before your paper is published online, we shall be distributing a press release to news organizations worldwide, which may very well include details of your work. We are happy for your institution or funding agency to prepare its own press release, but it must mention the embargo date and Nature Structural & Molecular Biology. If you or your Press Office have any enquiries in the meantime, please contact press@nature.com.

You can now use a single sign-on for all your accounts, view the status of all your manuscript submissions and reviews, access usage statistics for your published articles and download a record of your refereeing activity for the Nature journals.

If you have not already done so, we strongly recommend that you upload the step-by-step protocols used in this manuscript to the Protocol Exchange. Protocol Exchange is an open online resource that allows researchers to share their detailed experimental know-how. All uploaded protocols are made freely available, assigned DOIs for ease of citation and fully searchable through nature.com. Protocols can be linked to any publications in which they are used and will be linked to from your article. You can also establish a dedicated page to collect all your lab Protocols. By uploading your Protocols to Protocol Exchange, you are enabling researchers to more readily reproduce or adapt the methodology you use, as well as increasing the visibility of your protocols and papers. Upload your Protocols at www.nature.com/protocolexchange/. Further information can be found at www.nature.com/protocolexchange/about.

An online order form for reprints of your paper is available at https://www.nature.com/reprints/author-reprints.html. Please let your coauthors and your institutions' public affairs office know that they are also welcome to order reprints by this method.

Please note that <i>Nature Structural & Molecular Biology</i> is a Transformative Journal (TJ). Authors

may publish their research with us through the traditional subscription access route or make their paper immediately open access through payment of an article-processing charge (APC). Authors will not be required to make a final decision about access to their article until it has been accepted. Find out more about Transformative Journals

<B>Authors may need to take specific actions to achieve compliance with funder and institutional open access mandates. For submissions from January 2021, if your research is supported by a funder that requires immediate open access (e.g. according to Plan S principles) then you should select the gold OA route, and we will direct you to the compliant route where possible. For authors selecting the subscription publication route our standard licensing terms will need to be accepted, including our self-archiving policies. Those standard licensing terms will supersede any other terms that the author or any third party may assert apply to any version of the manuscript.

In approximately 10 business days you will receive an email with a link to choose the appropriate publishing options for your paper and our Author Services team will be in touch regarding any additional information that may be required.

You will not receive your proofs until the publishing agreement has been received through our system.

If you have any questions about our publishing options, costs, Open Access requirements, or our legal forms, please contact ASJournals@springernature.com

Kind regards,
Florian

Florian Ullrich, Ph.D.
Associate Editor
Nature Structural & Molecular Biology
ORCID 0000-0002-1153-2040
